# Splicing and editing of ionotropic glutamate receptors: a comprehensive analysis based on human RNA-Seq data

**DOI:** 10.1007/s00018-021-03865-z

**Published:** 2021-06-08

**Authors:** Robin Herbrechter, Nadine Hube, Raoul Buchholz, Andreas Reiner

**Affiliations:** Department of Biology and Biotechnology, Ruhr University Bochum, Universitätsstrasse 150, 44801 Bochum, Germany

**Keywords:** Next-generation sequencing (NGS), Splicing error, Nonsense-mediated decay (NMD), Single-nucleotide polymorphism (SNP), C-to-U editing, Primate-specific

## Abstract

Ionotropic glutamate receptors (iGluRs) play key roles for signaling in the central nervous system. Alternative splicing and RNA editing are well-known mechanisms to increase iGluR diversity and to provide context-dependent regulation. Earlier work on isoform identification has focused on the analysis of cloned transcripts, mostly from rodents. We here set out to obtain a systematic overview of iGluR splicing and editing in human brain based on RNA-Seq data. Using data from two large-scale transcriptome studies, we established a workflow for the de novo identification and quantification of alternative splice and editing events. We detected all canonical iGluR splice junctions, assessed the abundance of alternative events described in the literature, and identified new splice events in AMPA, kainate, delta, and NMDA receptor subunits. Notable events include an abundant transcript encoding the GluA4 amino-terminal domain, GluA4-ATD, a novel C-terminal GluD1 (delta receptor 1) isoform, GluD1-b, and potentially new GluK4 and GluN2C isoforms. C-terminal GluN1 splicing may be controlled by inclusion of a cassette exon, which shows preference for one of the two acceptor sites in the last exon. Moreover, we identified alternative untranslated regions (UTRs) and species-specific differences in splicing. In contrast, editing in exonic iGluR regions appears to be mostly limited to ten previously described sites, two of which result in silent amino acid changes. Coupling of proximal editing/editing and editing/splice events occurs to variable degree. Overall, this analysis provides the first inventory of alternative splicing and editing in human brain iGluRs and provides the impetus for further transcriptome-based and functional investigations.

**Supplementary Information:**

The online version contains supplementary material available at 10.1007/s00018-021-03865-z.

## Introduction

The nervous system shows a remarkable degree of differentiation, despite being built from a limited set of molecular and cellular entities. Splicing and RNA editing are important mechanisms to increase protein diversity and to adjust gene expression in a context-dependent manner. Splicing and editing are particularly widespread in the central nervous system (CNS) [1], where they contribute to differentiation [2, 3], synaptic organization [4], and the tuning of voltage-gated channels and receptors [5–7]. One important receptor family is the family of ionotropic glutamate receptors (iGluRs).

iGluRs are tetrameric, neurotransmitter-gated ion channels, which relay excitatory signals and control synaptic plasticity in the CNS [8, 9]. In mammals, 18 different iGluR subunits have been described [10, 11], which are grouped into four subfamilies: AMPA receptors (subunits GluA1-4) [12, 13], kainate receptors (subunits GluK1-5) [14, 15], delta receptors (GluD1 and GluD2) [16], and NMDA receptors [17, 18]. NMDA receptors are obligatory heteromers of two GluN1 and two GluN2 (GluN2A-D) or GluN3 (GluN3A/B) subunits. Heteromer formation also appears to prevail within the AMPA and kainate receptor subfamilies, which allows for the integration of different functionalities within single receptor complexes [19–21].

With cloning of the first iGluR transcripts from RNA libraries (reviewed in [10]), editing and splicing were recognized as important mechanisms to increase the functional diversity of iGluRs. Prominent examples include the mutually exclusive splicing of AMPA receptor flip/flop exons [22], which affects channel gating [23], and adenosine-to-inosine (A-to-I) RNA editing in the GluA2 pore region (also in GluK1 and GluK2) [24], which causes a glutamine-to-arginine (Q/R) substitution that abolishes the Ca^2+^ permeability of receptors carrying these subunits.

Most reported splice events in iGluR transcripts affect functional receptor domains. iGluR subunits share a homologous domain structure and consist of an extracellular amino-terminal domain (ATD), an extracellular ligand-binding domain (LBD), the channel-forming transmembrane domains (TMDs), and intracellular C-terminal domains (CTDs) [13, 25]. Glutamate binding at the LBDs closes the bi-lobed, clamshell-like LBDs, which induces the opening of the central ion pore. In AMPA and kainate receptors, glutamate binding also causes subsequent desensitization (temporary receptor inactivation), which can be attributed to rearrangements of the LBD dimer interfaces. AMPA receptor flip/flop splicing, for instance, affects gating by modifying this LBD dimer interface and the linker region, which connects the LBDs to the TMD [20, 26]. Most described splice events, however, affect the CTDs, which mediate interactions with other synaptic proteins and which are extensively controlled by post-translational modifications [8, 9, 11, 27]. Alteration of the CTDs has also important consequences for receptor trafficking and turnover (e.g., [28–30]). Apart from changes to the protein sequence, splicing may also affect the 5′- and 3′-UTRs, which have regulatory functions.

RNA splicing and editing are regulated by cell- and tissue-specific developmental programs [31–34]. This is particularly true for the brain, where splicing is intricately linked to neurogenesis and development [2, 3, 35], and where transcripts are known to differ between cell types and brain regions [1, 36–38]. The differential expression of iGluRs has been studied in great detail [39], along with some well-documented changes in iGluR splicing and editing. For instance, AMPA receptor flip isoforms are already expressed before birth, whereas flop variants become expressed postnatally [40]. GluA2 appears to become fully Q/R-edited during early neurogenesis [41, 42], whereas editing of the GluA2 R/G and other iGluR sites increases in later development [43, 44]. Lack of GluA2 Q/R editing is lethal [45–47] and even reduced editing has profound consequences [48]. Also activity-dependent changes in splicing and editing have been reported for GluA2 [49, 50]. Furthermore, many diseases have been linked to alterations in splicing (including splicing of iGluRs), which may influence the progression of these pathologies (see, e.g., [34, 51–55]).

Despite the widespread occurrence of iGluR splicing and editing, a systematic analysis is lacking, so far. The known iGluR isoforms were mostly identified by cloning of rodent transcripts, but information on their abundance often remains sparse and several reported isoforms appear to play only minor roles. Moreover, most research has focused on splicing events that appear to maintain the structural integrity of iGluRs. Databases such as GENCODE list numerous other transcripts, namely transcripts with alternative untranslated regions (UTRs), transcripts that may be subject to nonsense-mediated decay (NMD) [56]*,* and/or transcripts that may simply reflect erroneous splicing events. However, without consistent abundance information and annotations, it remains difficult to assess the role of individual isoforms. Quite importantly, it also remains unclear, how findings from rat and mice extrapolate to humans, since splicing and editing are highly variable between species (see, e.g., [57–60]), even within primates [61, 62].

**Fig. 1:**
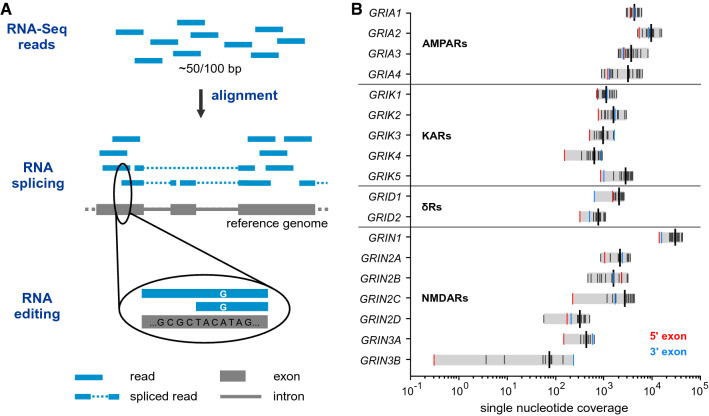
RNA-Seq data analysis and abundance of human iGluR transcripts. **A** Alignment of individual cDNA reads to a reference genome provides direct information on splice junctions and single-nucleotide mismatches. **B** Single-nucleotide coverage of the canonical iGluR exons over all 35 analyzed datasets (Table S1–3). Short bars indicate the coverage of individual exons; *red* and *blue* bars indicate 5′- and 3′-exons, respectively. Longer bars show the corresponding mean

In the last decade, next-generation sequencing (NGS) emerged as a powerful method to analyze splicing and editing on a transcriptome-wide level: Exon-spanning reads provide direct information for identifying splice junctions, and the number of mapped reads can be used to infer information on the junction abundance and the coverage of the corresponding exons (Fig. 1A). Similarly, RNA editing can be inferred from nucleotide mismatches between RNA-Seq reads and genomic sequences [63]. The detection of alternative splice events is now part of many automated pipelines ([64, 65]; see also Ensembl and GTEx databases), but even for small gene families, manual annotation and analysis are required for obtaining a meaningful overview.

Here, we set out to perform a systematic and comprehensive analysis of iGluR splicing and editing in humans. Using datasets from different brain regions that had been acquired in two large-scale RNA-Seq studies [66, 67], we obtained consistent abundance information for novel splice events as well as for splice events that were described for rat, mouse, or human or had been annotated automatically. We found that some reported events occur rather rarely and identified differences between splicing in rodent and primate species. Moreover, our exhaustive de novo identification of splice junctions revealed several new splice events, which occur with medium-to-high frequencies, pointing to the existence of several, hitherto unknown isoforms, as for instance confirmed for an alternative GluD1 isoform, GluD1-b, and a GluA4-ATD variant. Finally, we investigated RNA editing of human iGluR transcripts and analyzed the interdependence of close-by splicing and editing events.

## Materials and methods

We here give a brief method summary; full descriptions of the workflow (Fig. S1), bioinformatics tools, and individual parameters are given as Supplementary Information (SI) Methods.

### RNA-Seq data and canonical isoforms

RNA-Seq datasets from human brain tissues (compare SI Table S1) were prefetched from the Sequence Read Archive (SRA, NIH) using SRA Toolkit 2.8.2. These datasets originate from RNAs that were extracted by poly(A) selection (593.7 Gb, [66]) or ribosomal RNA depletion (99.8 Gb, [67]) and subjected to paired-end sequencing (Wu et al. [66]: 100 nt read length, Illumina HiSeq 2000; Labonté, et al. [67]: 50 nt read length, Illumina HiSeq 2500). For referencing purposes, canonical transcripts were defined (Table S2), which typically encompass the most frequent splice junctions.

### Alignments, quality control, and initial analysis

TopHat 2.1.0 [68] was used to align the reads to the human genome assembly hg38 GRCh38.p10 (Genome Reference Consortium) or to a reduced user-defined reference genome (udrg), which is based on the hg38 and encompasses the 18 iGluR genes with ± 1 Mbp flanking regions (see SI Methods and Fig. S1). Sorting and indexing of aligned reads were performed with SAMtools 0.1.19 [69]. Data quality was assessed with respect to overall read-mapping rates, junction information, and read quality (see Fig. S2) using RSeQC [70] and custom-written MATLAB scripts. The SAMtools mpileup function was used to analyze the nucleotide coverage (see SI Methods).

### Analysis of iGluR splice junctions

First, we obtained all known iGluR transcripts from the Ensembl 94 annotation [71] and extracted the corresponding splice junctions to an in-house isoform database. All junction-spanning reads identified during the alignments were then compared against the known splice donor and acceptor sites using a custom-written MATLAB pipeline. We identified and counted reads that match to known iGluR junctions (being part of Ensembl transcripts) and reads that indicate primary new junctions, i.e., those that encompass new donor–acceptor combinations or one new splice site (Fig. S4A). New splice sites served for the subsequent identification of secondary new junctions, i.e., junctions that encompass two new splice sites (cf. SI Methods). All identified junctions are listed in Table S4.

### Normalization

**Fig. 2:**
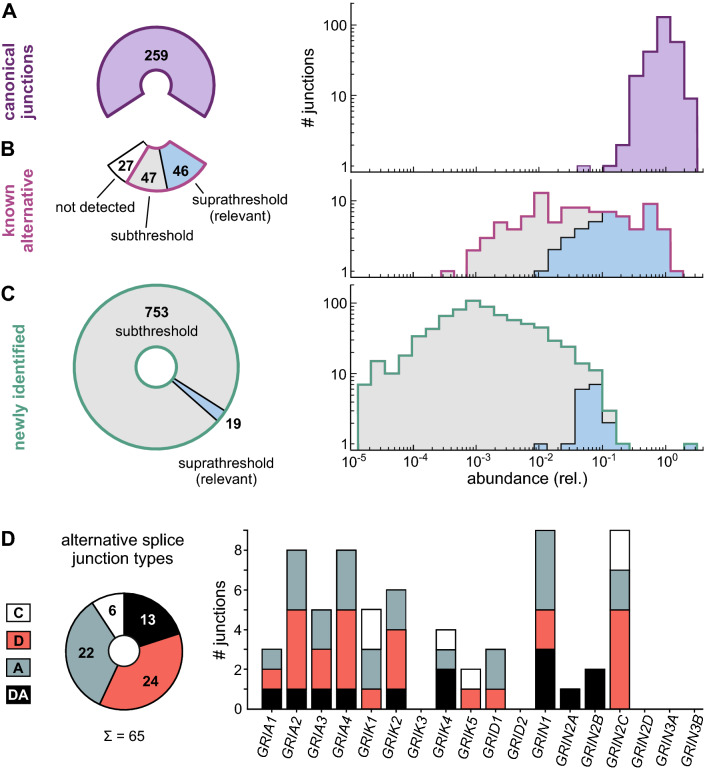
Analysis of canonical, known, and newly identified iGluR splice junctions. **A** All 259 known canonical junctions were identified. Normalization to the mean junction abundance of the respective subunit shows that canonical junctions belonging to a gene occur with rather similar abundance (see also Fig. S7A). **B** Most known alternative splice junctions from previously annotated transcripts were identified (93/120), but only 46 were classified as relevant based on our abundance criteria (*blue*). Normalization was performed with respect to the corresponding canonical junctions, see Methods. **C** Another 772 junctions were newly identified using a de novo identification approach, but based on their relative abundances, only 19 were classified as relevant (*blue*). **D** The relevant alternative junctions correspond to different events and iGluRs: C, alternative combination of canonical donor and acceptor sites; D, alternative donor site; A, alternative acceptor site; DA, alternative acceptor and donor sites. For splice site analysis see Fig. S6

Canonical splice junction counts were normalized to the mean of all canonical junctions belonging to that gene (Fig. 2A and Fig. S5A). Where possible, alternative junction counts were normalized to the canonical junction/s with identical splice site/s. Alternative junctions with two new splice sites were normalized to the closest canonical junction. The junction abundance was evaluated on the basis of individual datasets (local abundance) with sufficient coverage (see SI Methods) as well as globally, using the sum over all 35 analyzed data sets (Table S4). More abundant (‘relevant’) splice junctions (for criteria see main text) were evaluated manually, see Fig. 2 and Tables 1–4. We further tested for sex-specific differences using the data by Labonté et al. [67], but observed no statistically significant differences in the junction counts and local abundance (cf. SI Methods and Table S5). Specific MegaBLAST queries on RNA-Seq datasets from chimpanzee, macaque, rat, and mouse (Fig. S12–S20, S22 and Table S6) are described as SI Methods.

**Table 1:**
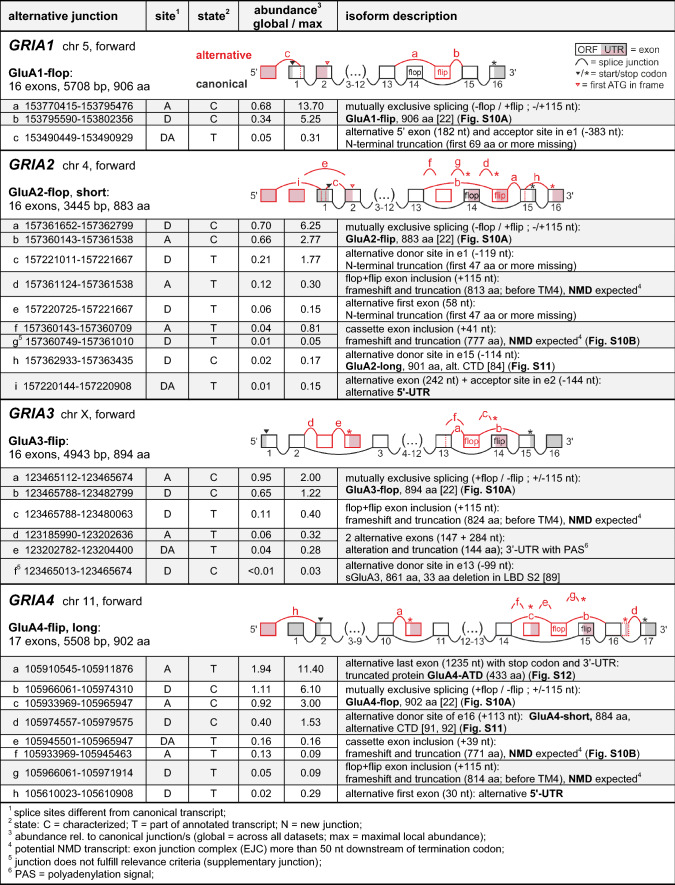
Alternative AMPA receptor splicing

**Table 2:**
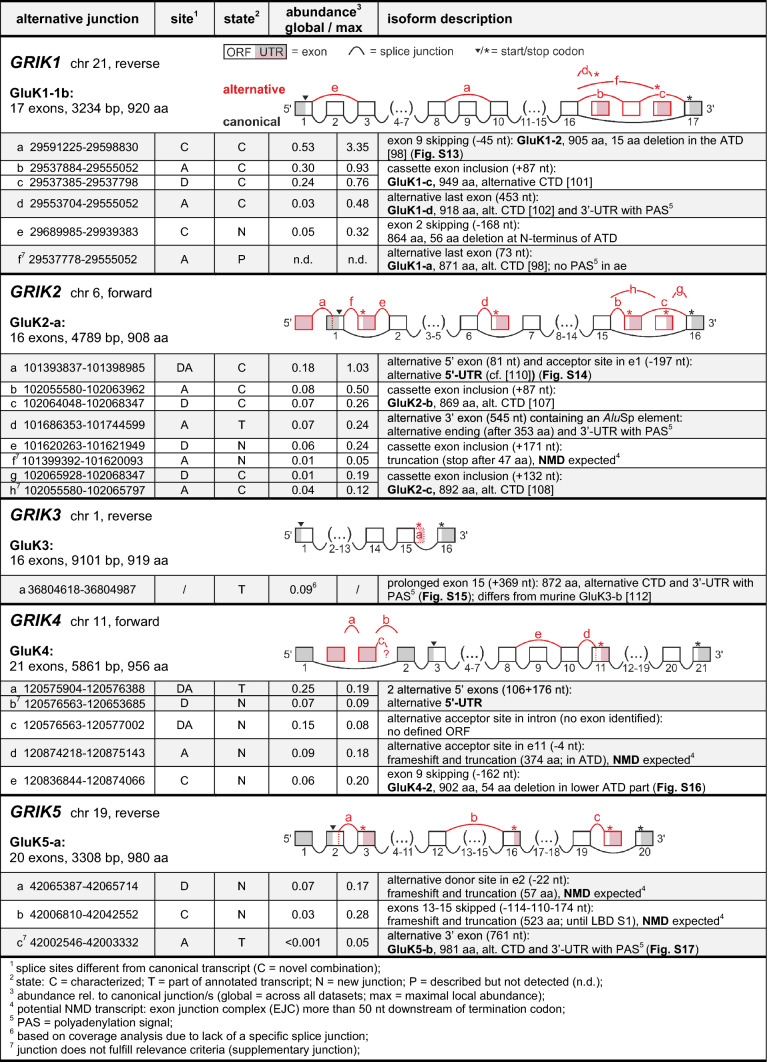
Alternative kainate receptor splicing

**Table 3:**
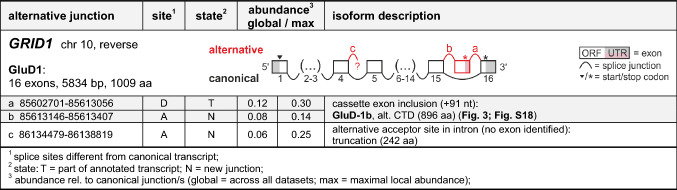
Alternative delta receptor splicing

**Table 4:**
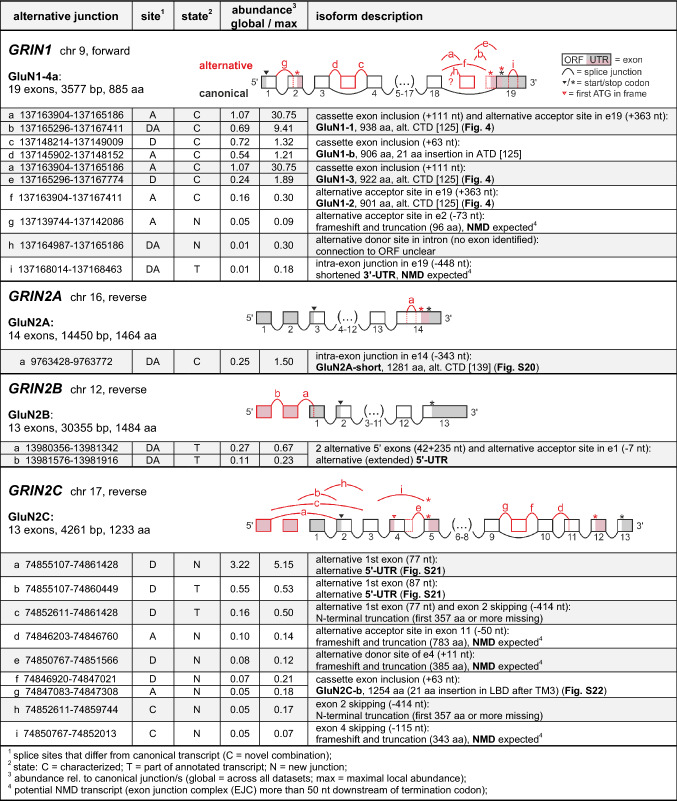
Alternative NMDA receptor splicing

### Analysis of RNA editing events

De novo identification of potential iGluR editing sites was based on variant calling using SAMtools mpileup (Fig. S24). Only frequent mismatches were considered and base exchanges had to be consistent with A-to-I or C-to-U editing (Table S7, Fig. 5A and SI Methods). The remaining mismatch positions were compared to known SNPs listed in the dbSNP (NIH) taking their reported abundance into account (Fig. S25). Figure 5B shows unequivocal editing positions in coding regions (see SI Methods and Table S7).

### Analysis of splicing and editing relationships

The combination of splicing events at the GluN1 C-terminus (Fig. 4) was analyzed using Pearson correlation analyses. Possible relations between proximal editing sites as well as between editing and close-by splice events (Fig. 5C,D) were analyzed using MegaBLAST [72] searches. Here the datasets were queried with specific sequences (40 nt) that encompassed the corresponding RNA editing or splicing events (Table S8).

### RT-PCR analysis of selected splice events

**Fig. 3:**
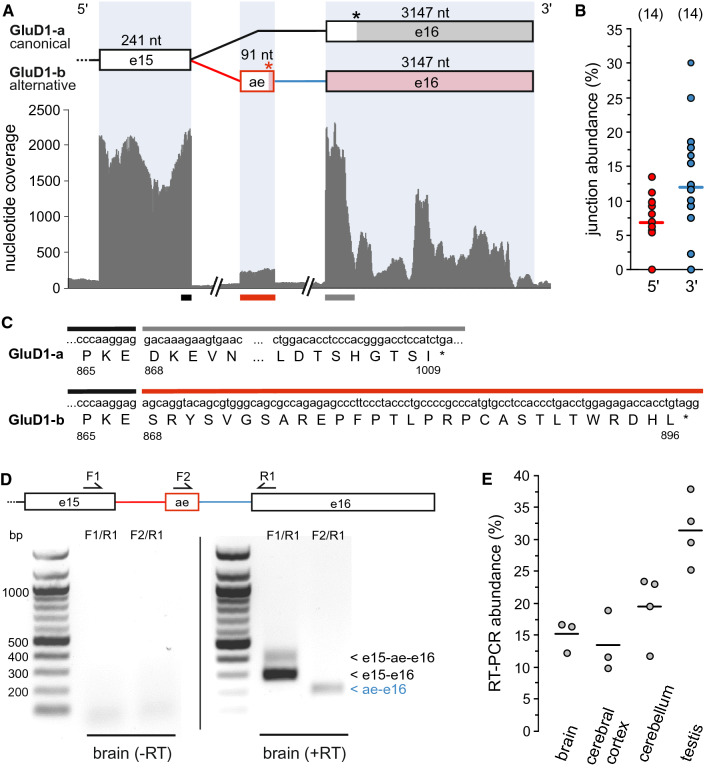
De novo identified GluD1-b splicing isoform. **A** Our analysis revealed the existence of splice junctions, which indicate the incorporation of a 91 nt alternative exon (ae) between canonical exons 15 and 16. The coverage track shows a clearly defined exon (stop codons indicated by *asterisks*). **B** 5′- and 3′-junction abundance relative to the canonical junction (median indicated as *bars*; number of datasets with sufficient coverage given in *parenthesis*). **C** Incorporation of the alternative exon results in a hitherto undescribed isoform, GluD1-b, (896 aa), which contains an alternative C-terminus. **D** RT-PCR detection in human brain RNA. Exon-specific primers (F1/R1) show the amount of transcripts with (376 bp) and without (285 bp) alternative exon. Primers F2/R1 confirm the identity of the alternative exon, which was further confirmed by sequencing. Bands are absent in negative controls without reverse transcriptase (-RT). **E** Relative amounts of alternative F1/R1 RT-PCR product in RNAs from different human tissues. For further information, see Fig. S18

The presence/absence of selected exons was verified in independent human RNA samples purchased from BioChain using RT-PCR (Fig. 3D,E and Figs. S12D, S16C,D and S18D,E). cDNAs were prepared using the Maxima H Minus First Strand cDNA Synthesis Kit (Thermo Scientific) and non-saturating PCRs were performed using exon-spanning primer pairs. Semi-quantitative analysis of gel images was made with ImageJ 1.51f. For details see SI Methods.

## Results

### iGluR transcripts in RNA-Seq data from human brain tissues

For our study, we chose 35 different publicly available RNA-Seq datasets from two unrelated large-scale transcriptome studies [66, 67]. In total, the datasets comprised 8.18∙10^9^ reads of 50 or 100 nt length from different brain regions and individuals (693.5 Gbases; see Table S1 and Fig. S1). Alignment to the human reference genome hg38 showed reasonable overall data quality (see SI Methods, Fig. S2). Only a low percentage of reads mapped to intronic sequences, as expected for reads that mostly originate from processed transcripts. Subsequent analyses were performed with a user-defined reference genome, which only encompassed the iGluR genes and adjacent chromosomal regions (± 1 Mbp; see SI Methods).

To compare the transcript abundance of the different iGluRs, we determined the mean single-nucleotide coverages of their canonical exons across all datasets (Fig. 1 and Tables S2, S3). The highest coverage was obtained for exons belonging to the NMDA receptor subunit GluN1 (*GRIN1*), which were covered on average with 30,080 reads per nucleotide position. The median iGluR average coverage was 1,848 reads per nucleotide position. *GRIN2D* and *GRIN3A* exons showed an average single-nucleotide coverage < 500; *GRIN3B* was hardly detectable (Fig. 1B).

As expected, transcript expression differed between different datasets and preparation methods (Fig. S3). Still, it should be noted that individual datasets cannot provide reliable information for comparing iGluR expression in different brain regions. Nevertheless, with the exception of *GRIN3B*, the aggregated data should allow for reliable detection and quantification of splice junctions as well as nucleotide mismatches caused by RNA editing.

### Detection and analysis of splice junctions

Our aim was to identify and quantify iGluR splicing using direct experimental information. We thus focused our analysis on sequencing reads that mapped to splice junctions: These reads align at two distant gene regions, thereby revealing the corresponding splice donor and acceptor sites (Fig. 1A and Fig. S4).

From our alignment, we extracted 1,747,402 junction-spanning reads, which contained 1124 unique iGluR junctions (Table S4). A large fraction of these junctions were sampled rarely (55% in ≤ 10 reads; Fig. S5), but all shared the characteristics of major spliceosome U2 donor and acceptor splice sites (Fig. S6). We continued by classifying each junction as either (i) belonging to a canonical (reference) isoform, (ii) being an alternative junction, which has already been part of a human transcript reported in the Ensembl database (GENCODE; [71], or (iii) being a newly identified junction (Fig. 2A–C). Details on the workflow and de novo identification of splice junctions are reported in the Methods section.

For referencing purposes, we defined canonical iGluR isoforms, which typically represent the most frequent splice events (all are human Ensembl transcripts; Table S2). We detected all iGluR junctions belonging to the canonical isoforms (259/259), most of them with high coverage (Fig. S5, S7). The canonical junctions of *GRIN1*, which is the highest expressing iGluR gene, were covered with 17,541–78,603 reads. Even for low expressing iGluRs, such as *GRIN2D* and *GRIN3A*, we detected 60–967 reads per junction. Only the junctions of *GRIN3B*, which shows extremely low transcript levels (Fig. 1B), were covered poorly, i.e., with only a few reads. Within individual iGluR genes, the canonical junction abundance was rather uniform, as seen by a narrow distribution after normalization to the respective mean abundances (standard deviation 0.42; Fig. 2A and Fig. S7).

Besides the canonical junctions, we also detected 93 known alternative iGluR splice junctions out of 120 alternative junctions that were present in the human transcripts in the Ensembl database (Fig. 2B). To estimate the relative abundance of alternative junctions, we normalized their read counts to the corresponding canonical junctions. In cases, where this was not possible, we used the closest canonical junction for normalization (see SI Methods). Normalization shows that a large Fraction of the known alternative junctions occurs rather rarely, as 58% (54/93 junctions) had a relative abundance ≤ 0.05 (Fig. 2B). We thus limited our subsequent analysis to more abundant events: We classified junctions as likely relevant, if they were covered with ≥ 35 reads, and if they had either an overall (global) abundance of ≥ 0.05 or were clearly enriched in individual datasets (local abundance ≥ 0.15). These criteria were met by 46 of the 120 known alternative iGluR junctions (38%) (Fig. 2B), i.e., based on these criteria, more than half of the reported splice events may only play a minor role in adult human brain.

In addition to 352 known splice junctions, we detected 772 novel splice junctions (Fig. 2C). Of those, 728 encompass known donor and/or acceptor sites (primary novel junctions), and 44 contain both, a new donor and a new acceptor site (secondary novel junctions; Fig. S4A). However, most newly identified splice junctions had negligible abundance: Only 2.5% (19/772 junctions) met our relevance criteria and were analyzed further. The large number of low-abundance junctions likely reflects erroneous splice events, i.e., noise [73]. In any case, it seems unlikely that we missed splicing events within our datasets that would have met our relevance criteria, since the number of unique canonical and relevant iGluR junctions saturated early (Fig. S8).

We next analyzed how the relevant alternative junctions differed from the canonical transcript junctions (Fig. 2D). In 37% (24/65 junctions), an alternative donor site (D) was present, in 34% (22/65) an alternative acceptor site (A), and in 20% (13/65) both an alternative donor and acceptor site (DA), which argues against particular detection biases. The remaining junctions, 9% (6/65), showed an alternative combination of canonical donor and acceptor sites (C), i.e., an exon skipping event. Applying our relevance criteria, we detected alternative splicing of all human iGluR genes, except for *GRIK3*, *GRID2*, *GRIN2D, GRIN3A,* and *GRIN3B* (Fig. 2D). Most relevant junctions were linked to either known or novel exons (Tables 1–4; Fig. S4B); only for 3/65 junctions we were not able to trace the junction to another exon, i.e., they appeared to recede in intronic regions. In the following sections, we summarize the relevant splicing events observed in AMPA, kainate, delta, and NMDA receptors and compare them to literature data.

### Alternative splicing of AMPA receptor subunits

The relevant splice junctions belonging to the *GRIA1-4* genes are shown in Table 1; for rare events, see Table S4. Information on the canonical reference isoforms is given in Table S2.

The AMPA receptor subunit GluA1 was the first cloned iGluR [74], which was followed by the identification of the subunits GluA2, GluA3, and GluA4 [10, 75]. At the same time, it was recognized that all four GluA subunits are expressed as flop and flip isoforms, due to the inclusion of mutually exclusive exons of 115 nt length [22]. The corresponding flop and flip segments (38 aa) differ in 8–10 amino acid (aa) residues and are located at the end of the LBD S2 segment, where they contribute to the LBD dimer interface [26, 76] and the S2-TM4 linker region [20]. The flop/flip isoform choice can have pronounced effects on desensitization [20, 23, 77], assembly and trafficking [78–80], as well as regulation by allosteric modulators, anions, and TARPs [26, 76, 81]. As expected, we identified junction-spanning reads for inclusion of the flop or flip exons in all four AMPA receptors (Table 1). The ratios of flop to flip junctions varied across datasets (Figs. S9, S10A), which is consistent with reported expression preferences in different brain regions and cell types [22, 23, 37, 40, 82, 83]. However, overall, flop and flip transcripts were detected at similar abundance (Table 1). Flop/flip splicing is thus one of the most frequent alternative splice events in iGluRs (Fig. S9), which underlines its physiological importance. The regulatory mechanisms of flop/flip splicing remain unknown, but activity-dependent changes were observed after neuronal silencing with TTX [49] and in a mouse model of Rett syndrome [53].

Next to the junctions that indicate proper splicing of the flop or flip cassettes, we also found junctions, which link the flop and flip exons (*GRIA2* (d) 12%, *GRIA3* (c) 11%, *GRIA4* (g) 5%, Table 1; for *GRIA1* 0.7%, Table S4). Inclusion of both exons results in a frameshift introducing an early stop codon just before TM helix 4 (GluA1 806 aa; GluA2 813 aa; GluA3 824 aa; GluA4 814 aa) and points to erroneous and/or incomplete splicing. These transcripts should be degraded by nonsense-mediated decay (NMD), since the premature stop codon is followed by several downstream splice junctions > 50 bp away [56]. In contrast, the numbers of junctions pointing to simultaneous removal of both the flop and flip exon were rather low for all subunits (global abundance < 1%; Table S4). These events again result in a frameshift, early truncation, and likely NMD. For *GRIA2* and *GRIA4*, we detected additional junctions into and out of another cassette exon, which is located right before the flop exon (*GRIA2* (f, g) and *GRIA4* (e, f)*,* Table 1). Also these transcripts code for truncated subunits and should be subject to NMD. Interestingly, these cassette exons are not conserved between *GRIA2* and *GRIA4*, but across species (Fig. S10B).

For *GRIA1*, only one other splice junction met our relevance criteria, namely splicing from an alternative 5’-UTR exon to an alternative acceptor site in canonical exon 1 (*GRIA1* (c), Table 1). However, this transcript would result in an N-terminally truncated subunit, as it lacks the original start codon, signal peptide, and a part of the ATD.

Also splicing in the AMPA receptor C-terminal regions is partly conserved between different subunits, which is exemplified by the GluA2-long isoform (901 aa) [84]. This isoform results from an alternative splice donor site in the penultimate exon, which prolongs the reading frame to a stop codon in the last canonical exon. The C-terminus of GluA2-long lacks the C-terminal type II PDZ binding motif [85] and is homologous to the C-terminus of the canonical GluA1 and the GluA4-long isoform (Fig. S11). We detected the GluA2-long junction in 18/35 human datasets, but with low frequency compared to the corresponding canonical junction (global abundance 2%; Table 1). Also in rat, this isoform was reported to occur at < 10% abundance [84].

Several studies addressed variations in the 5′- and 3′-UTRs of *GRIA2*, which include a polymorphic GU-repeat domain in humans [84, 86] and different polyadenylation sites in the 3′-UTRs [87], which contain regulatory microRNA binding regions (see [88]). In addition, we found alternative splicing in the 5′-UTR (Table 1). However, two of these events, *GRIA2* (c) and (e), would result in N-terminally truncated receptors without signal peptides; the third one occurs rather rarely (*GRIA2* (i)).

GluA3 is subject to flip/flop splicing as described above (Table 1). A GluA3-long isoform does not exist, since the corresponding alternative 5’-donor site is missing in *GRIA3* (Fig. S11; [84]). Apart from this, we detected junctions to two alternative exons, which, however, would result in a drastically shortened and altered ORF encoding 144 aa (*GRIA3* (d, e)). Another GluA3 variant with a dominant-negative phenotype has been described to occur in the rat cochlea [89, 90]. In humans, this isoform would result from an alternative donor site in exon 13 (*GRIA3* (f)), but in brain datasets, the corresponding junction was only present at low levels (< 1%; Table S4).

For GluA4, in addition to flip/flop splicing, alternative splicing is known to produce two C-terminal isoforms, GluA4-long (902 aa) and GluA4-short (884 aa; also named GluA4-c) [91, 92]. The GluA4-short C-terminus is homologous to the C-termini of GluA2-short (the canonical GluA2 isoform) and GluA3 (Fig. S11). In rodents, the GluA4-long isoform may prevail, since GluA4-short transcripts were mainly observed in the cerebellum [91]. In humans, however, we detected the corresponding GluA4-short junction (*GRIA4* (d), Table 1) frequently and almost ubiquitously in 31 of 35 datasets occurring with a global abundance of ~ 40% (Fig. S9), which is in line with previous RT-PCR data [92]. Despite the abundance and loss of a PDZ binding motif in GluA4-long (Fig. S11C; [85, 93]), the physiological role of GluA4 C-terminal splicing has not been investigated so far.

Quite surprisingly, also no literature information can be found for an alternative *GRIA4* splice junction that we detected at high abundance (194%) in all neuronal human datasets (*GRIA4* (a), Table 1 and Fig. S9), but also in RNA-Seq data from other primate and murine species (Fig. S12). This junction connects canonical exon 10 to an alternative exon, which introduces an early stop codon after 433 aa followed by an alternative 3′-UTR and a polyadenylation signal (PAS) (Table 1 and Fig. S12). The resulting protein, which we termed GluA4-ATD (49 kD), would encompass the signal peptide and ATD of the full-length receptor followed by an additional 10 aa tail with a partly unique sequence (Fig. S12C). Using RT-PCR analysis with exon-specific primers, we independently confirmed the presence of the transcript in RNA from different human brain regions (Fig. S12D). Furthermore, mass spectrometric data suggest that this unusual isoform is expressed on the protein level, as we found a specific peptide matching the alternative GluA4-ATD (Fig. S12E) in a human brain proteome search [94, 95]. Future studies will have to address the expression of the GluA4-ATD isoform and possible physiological functions. In this context, it may be interesting to note, that the GluA4 ATD is involved in pentraxin interactions [96, 97]. Less frequent *GRIA4* splicing events include the usage of an alternative 5′-UTR exon (*GRIA4* (h)), and inclusion of a 39 nt cassette exon before the flop exon (*GRIA4* (e, f)), see flip/flop splicing; Fig. S10B).

### Alternative splicing of kainate receptor subunits

Several splicing events have been described for kainate receptors [15], most of them for the GluK1 subunit. When GluK1 was first cloned from rat [98], two isoforms were identified, GluK1-1 and GluK1-2. These isoforms differ by the inclusion of a cassette exon that codes for a 15 aa insertion close to the end of the ATD (Table 2; Fig. S13AB). Although this insertion is not seen in any other iGluR, it appears to prevail in GluK1, as we detected the junctions corresponding to GluK1-1 more frequently than the more typical GluK1-2 junction (*GRIK1* (a)), which occurred at 53% abundance compared to GluK1-1. In ‘fetal brain’, however, the GluK1-2 isoform was threefold more abundant (Table S4). Queries of datasets from other species suggest similar overall abundances (Fig. S13C). Interestingly, the physiological and functional implications of GluK1-1/2 splicing remain unknown. The affected ATD/LBD-linker region has modulatory functions in other iGluRs (NMDA receptors; [99]), but experimental data are only available for the GluK1-2 isoform, with the exception of a study that reported a different sensitivity of GluK1-1 towards NS3763, a non-competitive inhibitor [100].

Moreover, four C-terminal GluK1 variants, GluK1-a–d, have been described [98, 101, 102]. In all analyzed human datasets (except ‘dura mater’), the GluK1-b isoform [98] junction was the most abundant junction. We thus defined GluK1-1b as canonical isoform [29, 103]. Also the two junctions reporting on the GluK1-c isoform occurred frequently in humans (24% and 30% abundance, respectively; *GRIK1* (b,c)) Table 2) and other species (Fig. S13D). GluK1-c is known for poor trafficking in heterologous systems, but may play an important role in controlling presynaptic inhibition at immature synapses [104]. The junction encoding the GluK1-d isoform (*GRIK1* (d)) was only enriched in a single dataset (‘subiculum’), and we did not detect a single junction-spanning read for GluK1-a (*GRIK1* (f)), which is the shortest reported GluK1 isoform [101]. Specific queries showed that this isoform is also weakly expressed in mouse and rat brain (< 2% abundance; Table S6; Fig. S13D), which is in agreement with earlier studies that detected GluK1-a in the spinal cord and brain stem, but not the mouse forebrain [29, 104, 105]. Notably, the annotated GluK1-a 3′-UTR in human, mouse, and rat lack a polyadenylation signal. Nevertheless, most functional data reported in the literature were obtained with the GluK1-a isoform, as it shows favorable trafficking in heterologous expression systems. Besides known splicing variants we also detected skipping of canonical exon 2 (*GRIK1* (e)), which preserves the signal peptide but deletes 56 aa in the N-terminal part of the ATD (864 aa), but overall this event occurs rather rarely.

For GluK2, two alternative isoforms, GluK2-b and GluK2-c, are known, which differ in their CTDs compared to the canonical GluK2-a isoform, due to the inclusion of different cassette exons before the last canonical exon [106–108]. In human datasets, the GluK2-a junction prevailed (Table 2), while we detected the two GluK2-b specific junctions (*GRIK2* (b,c)) with ~ 8% abundance (see also Fig. S9). GluK2-a is known for particularly effective membrane trafficking [29], whereas heteromer formation with GluK2-b is thought to allow for additional intracellular interactions, e.g., with Ca^2+^ signaling associated proteins [109]. We detected also detected reads belonging to the GluK2-c isoform junctions (*GRIK2* (g,h); Table 2), but at even lower levels (Fig. S9). This is in line with previous reports that GluK2-c may be more common in human non-neuronal tissues [108]. Similar abundances of these C-terminal isoforms are also seen in other species (Fig. S14C).

Apart from the known GluK2 isoforms, we identified a frequently occurring junction that points to an alternative human 5′-UTR (*GRIK2* (a), maximal abundance 1.03; Fig. S14A). The corresponding splice event has been detected before [110], but the 5′-exon appears to be shorter than reported. Apart from splicing, a polymorphic TAA region is present in the 3′-UTR [111]. Furthermore, we detected two splice events, which occur at low-to-moderate abundance, but encode truncated subunits. One is splicing to an early termination exon, which appears to contain an *Alu* element (*Alu*Sp) (*GRIK2* (d), Table 2). In the other case, the inclusion of a cassette exon (*GRIK2* (e,f)) introduces an early stop codon, which should mark the transcript for NMD.

For GluK3, one alternative splicing isoform has been described, GluK3-b. It results from a prolonged penultimate exon found in rat [112] and carries an alternative CTD that reduces surface trafficking [113]. However, in human RNA-Seq data, we found no reads covering this alternative junction and inspection of genomic sequences shows that the corresponding splice donor site is absent in primates (Fig. S15). The human nucleotide coverage remains slightly increased beyond the canonical splice site, which may indicate a 369 nt elongation of canonical exon 15, which would then end with an alternative polyadenylation signal (*GRIK3* (a)). However, the resulting C-terminal sequences appear to be poorly conserved between primates (Fig. S15), which suggests that this event does not constitute a major alternative GluK3 isoform. We also did not identify any other *GRIK3* junctions with relevant global or local abundance (for rare events see Table S4).

To our knowledge, no splicing isoforms have been described for the ‘high-affinity’ kainate receptor subunit GluK4 (formerly KA1; [114]). We detected *GRIK4* reads somewhat less frequently than reads of the other kainate receptor subunits, which is consistent with its limited expression in adult murine and human brain [114, 115]. However, we identified splice junctions that indicate that some of the transcripts may carry two alternative exons upstream of canonical exon 2, which would result in an alternative 5′-UTR (*GRIK4* (a,b), Table 2). Another notable event is skipping of exon 9, which causes a 54 aa deletion in the GluK4 ATD (Table 2 and Fig. S16). The corresponding junction (*GRIK4* (e)) occurs with moderate abundance of up to 20% in human datasets (6% global abundance), which was also confirmed by RT-PCR on human RNA samples (Fig. S16CD). Visual inspection shows that the structural integrity of the ATDs may be maintained by the sequence deletion in this potential GluK4-2 isoform (902 aa) (Fig. S16E). We detected the same exon skipping event also in other species, albeit at lower abundance (Fig. S16). Still, experimental work seems warranted to confirm the expression of this potential isoform and its functional consequences.

For GluK5, the second ‘high-affinity’ kainate receptor subunit (formerly KA2), an alternative GluK5-b isoform (981 aa), has been mentioned [8]. However, no experimental data were reported for this isoform, which would result from splicing to an alternative last exon that encodes an alternative C-terminus and 3′-UTR (Fig. S17). Our analysis shows a very low coverage of the corresponding exon and splice junction (*GRIK5* (c) junction abundance < 1%), with a slight enrichment in the ‘cerebellum’ dataset (junction abundance 5%; Fig. S9). The physiological significance of this event remains questionable, also because the GluK5-b exon is poorly conserved and rarely detected in chimpanzee and macaque (Fig. S17). In rat and mouse, a homologous exon is absent. Besides, we identified two novel junctions with low-to-moderate abundance (*GRIK5* (a) and (b), Table 2), which do not yield full-length GluK5 subunits but may result in NMD; for rare events, see Table S4.

### Alternative splicing of delta receptor subunits

The delta receptors GluD1 (GluRδ1) and GluD2 (GluRδ2) were cloned based on sequence homology to other iGluRs [116, 117]. They are expressed throughout the brain, and, although they do not appear to function as glutamate-gated ion channels per se, they play important roles in synapse maturation and plasticity [16, 118]. No delta receptor isoforms have been reported, so far. However, we identified two *GRID1* junctions (*GRID1* (a,b)), which indicate the insertion of a 91 nt cassette exon before the last canonical exon, exon 16 (Table 3 and Fig. 3A). Both, the single-nucleotide coverage and the relative junction abundances (Fig. 3B) suggest that this cassette exon may be included in ~ 10% of the transcripts. More, the splice event appears to be conserved across species, as we detected both alternative junctions at similar abundance in chimpanzee, macaque, rat, and mouse datasets (Fig. S18).

Importantly, this alternative splice event may lead to a functional but hitherto undescribed GluD1 isoform, which we termed GluD1-b. The cassette exon provides an alternative CTD sequence, which starts ~ 15 aa after TM helix 4 and ends with an early stop codon after 896 aa, in contrast to the canonical GluD1-a isoform with 1009 aa (Fig. 3C). The 3′-UTR and the polyadenylation signal are still provided by canonical exon 16.

To further validate the presence of GluD1 transcript isoforms, we performed reverse transcriptase (RT)-PCRs on RNA samples from human brain (Fig. 3D). Amplification with primers that bind to the alternative exon ‘ae’ and exon 16 (F2/R1) yielded a PCR product of the expected size, and sequencing confirmed the anticipated splice event (Fig. S18). A second primer pair, which binds to canonical exons 15 and 16 (F1/R1), yielded two PCR products, as expected for partial inclusion of the alternative GluD1-b exon. Semi-quantitative analysis showed inclusion in ~ 15% of the brain transcripts (Fig. 3E), which is in good agreement with our estimate based on RNA-Seq data. We also confirmed the presence of GluD1-b in RNA from human cerebral cortex, cerebellum, and testis (Fig. 3E and Fig. S18). We believe that this rather frequent and conserved isoform warrants further investigation, also because a recent study highlighted the role of the GluD1 C-terminus for trafficking in neurons [119]. Apart from this, we detected one other *GRID1* junction (*GRID1* (c)), with low-to-moderate frequency, which reached from canonical exon 4 into the subsequent mega intron (222,579 bp). In this case, however, we were not able to identify exon-like features (Table 3).

For *GRID2*, we found lower transcript levels than for *GRID1*, apart from strong expression in the ‘cerebellum’ dataset (Fig. S3). This is in line with previous reports that showed some GluD2 expression throughout the mouse brain [118, 120] but prominent expression in cerebellar Purkinje cells [16, 117, 121]. We detected all canonical *GRID2* junctions but no alternative junctions at clearly relevant levels (for rare events, see Table S4).

### Alternative splicing of NMDA receptor subunits

Within the NMDA receptor subfamily [17, 122], alternative splicing has been foremost reported for the GluN1 subunit. GluN1 is abundantly and ubiquitously expressed in the CNS, and, being an obligatory subunit of all NMDA receptors, it is the only essential iGluR subunit.

Three different GluN1 splice events are known that combine to eight isoforms (e.g., [123–125]). The first event is the inclusion of a cassette exon between canonical exons 3 and 4 in humans (termed exon 5 in rodents)*,* which encodes a 21 aa insertion in the ATD (denoted as GluN1-b isoforms; Table 4). Our analysis shows that this cassette exon is frequently used, but in most datasets, the canonical GluN1-a junction was somewhat more abundant (Fig. S9). Insertion of the GluN1-b segment, which is located at the ATD–ATD and ATD–LBD dimer interface, modulates glutamate affinity, Zn^2+^ and proton sensitivity, and deactivation kinetics depending on the partnering GluN2 subunit (e.g., [124, 126–129]). In the mouse cortex, GluN1-b isoforms may be primarily present in interneurons [37]. Recent knockout studies directly show that this splice event is important for regulating synapse maturation as well as long-term potentiation in mice [130, 131]. Moreover, the GluN1-a/GluN1-b splice ratio has been shown to be affected by psychiatric diseases [51, 54].

**Fig. 4:**
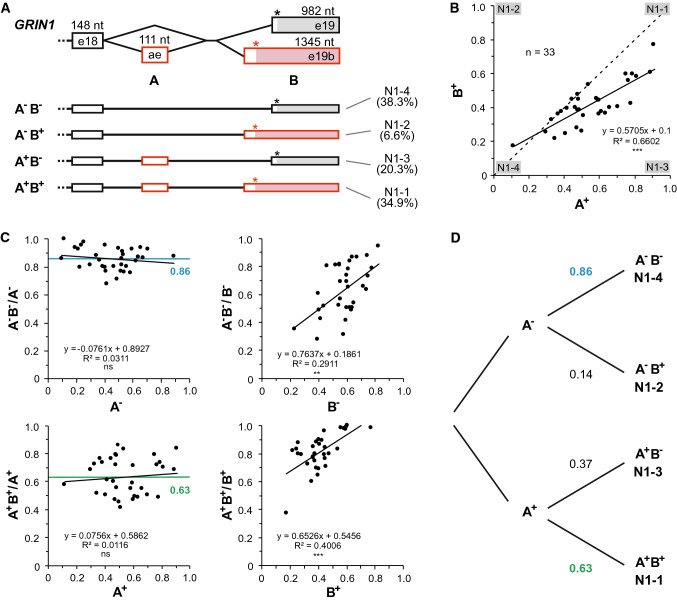
Alternative splicing in the C-terminal region of GluN1. **A** We detected all four C-terminal isoforms, which originate from incorporation of an alternative exon (ae) and the presence of two different acceptor sites in exon 19 [124]. **B** A comparison of splicing in different datasets (*n* = 33) shows a positive correlation between incorporation of the alternative exon (*A*^+^) and usage of the alternative acceptor site (*B*^+^). For other species, see Fig. S19. **C** A correlation analysis indicates that the absence or presence of the alternative exon (*A*^−^/*A*^+^) predicts the choice of the acceptor site (*B*^−^/*B*^+^; *left*), but not vice versa (*right*). **D** Resulting probability diagram for splicing to the alternative acceptor site

The second event is the inclusion of a cassette exon between canonical exons 18 and 19, which adds a 37 aa segment in the CTD (GluN1-1 and GluN1-3 isoforms; Fig. 4A). The third event is splicing to an alternative acceptor site, which causes a 5′ extension of exon 19. This shifts the reading frame and results in different C-termini of GluN1-1 and GluN1-2 compared to GluN1-3 and GluN1-4, respectively. We detected junction-spanning reads for all four C-terminal splice combinations (GluN1-1 to GluN1-4) to significant extent (Table 4 and Fig. 4A). Notably, in some datasets, the GluN1-1 isoform (combination of cassette exon inclusion and splicing to the alternative acceptor site) is highly favored compared to the canonical GluN1-4 isoform (up to 31-fold; Fig. S9). The GluN1-2 isoform was the least abundant isoform and junction-specific queries showed that this isoform is also underrepresented in chimpanzee and macaque datasets (Fig. S19). In rat and mouse, GluN1-3 appears to be the least abundant isoform (Fig. S19; [125, 132]).

Given that the GluN1 CTD composition controls trafficking and protein interactions, for instance with PSD-95 and calmodulin (e.g., [8, 17, 28, 133–136]), we further analyzed the occurrence of different splice combinations within individual datasets. The C-terminal splice events are clearly correlated: Datasets with high levels of cassette exon inclusion (*A*^+^) also show high levels of alternative acceptor usage (*B*^+^) (Fig. 4B).

Since individual reads can provide direct information on how the two splice events are combined, we performed a more detailed correlation analysis by asking, whether the presence or absence of the cassette exon (*A*^+^ or *A*^−^) can be used to predict how much splicing to either acceptor site (*B*^+^ or *B*^−^) occurs, or, vice versa, whether the presence of either acceptor site may be predictive for cassette exon inclusion (Fig. 4C). We find that in the absence of the cassette exon (*A*^−^), the canonical acceptor site (*B*^−^) is strongly favored (86:14) over the alternative acceptor site. In the presence of the cassette exon (*A*^+^), the alternative acceptor (*B*^+^) is somewhat favored over the canonical site (63:37). Quite to the contrary, the type of acceptor site (*B*^+^ or *B*^−^) cannot be used to predict the inclusion of the alternative exon. Mechanistically, this could indicate that the absence or presence of the cassette exon controls the choice between the two splice acceptor sites. A strong correlation between cassette exon inclusion (*A*^+^) and alternative acceptor usage (*B*^+^) is also seen in other primate and rodent species (Fig. S19B). Further research seems warranted, also to address potential activity-dependent [28] and cell type-specific effects. Apart from these well-described isoforms, we only detected three other GluN1 splice events at low levels (Table 4). An early truncation (*GRIN1* (g)) and a 3'-UTR exitron (intra-exonic splice junction *GRIN1* (i); −448 nt) appear to encode for NMD transcripts, whereas the third junction (*GRIN1* (h)) originates from an intronic region.

GluN2A was cloned from rat and mouse [137, 138], where no splicing isoforms are known. However, we detected an abundant exitron (*GRIN2A* (a)), which removes 343 nt from the last human canonical exon. The resulting frameshift alters and shortens the CTD to yield a GluN2A isoform with 1281 aa (Table 4 and Fig. S20). The same isoform, named GluN2A-short, was recently reported by Warming et al*.* to be expressed in human brain and to form functional receptors upon coexpression with GluN1 [139]. Our junction analysis shows that the GluN2A-short isoform is abundantly expressed in human brain (global abundance 25%), in some datasets even on par with the canonical isoform (Fig. S9). We detected the GluN2A-short splice junction with similar frequencies in chimpanzee and macaque datasets (Fig. S20B), but the corresponding splice sites are absent in rat and mouse, which supports the suggestion that this is a primate-specific isoform [139]. Shortening of the GluN2A CTD should have important functional consequences, as it results in a loss of several interaction motifs, including CaMKII and PSD-95 binding sites [27, 122]. We did not identify any other relevant splice junctions belonging to *GRIN2A*.

To our knowledge, no alternative splicing events have been reported for GluN2B other than variations in the mouse 5′-UTR [140]. Similarly, we did not identify any significant junctions that would cause changes to the coding sequence of human GluN2B, but we detected two moderately abundant junctions, which define a chain of two additional 5′-UTR exons (*GRIN2B* (a,b), Table 4). Despite high coverage, we did not detect any new junctions in the long 3′-UTR (*GRIN2B* > 22 kb; *GRIN2A* 9.8 kb).

GluN2C is known for its high expression in the cerebellum (see [138, 141, 142]) and we observed the highest transcript levels in the ‘cerebellum’ dataset (Fig. S3). The most frequent splice events in our analysis point to the existence of alternative 5′-UTR exons, which can replace canonical exon 1 (*GRIN2C* (a) and (b), Table 4 and Fig. S21). The newly identified junction *GRIN2C* (a) was 3.22-times more abundant than the canonical 5′-exon junction and prevailed in all datasets with sufficient coverage (Fig. S9). This transcript may thus be considered the primary UTR isoform, which is also supported by the coverage track (Fig. S21A). The second alternative 5′-exon junction was somewhat less abundant than the canonical junction (*GRIN2C* (b), Table 4), and a 5′ elongation of exon 2 has been reported, as well (Fig. S21B). In contrast, the *GRIN2C* 5′-UTR variations described in mouse [143], and the two alternative translation starts reported in rat [141], have no correspondence in humans.

Another notable, newly identified *GRIN2C* event is the insertion of a 63 nt cassette exon between canonical exons 9 and 10, which would result in a 21 aa insertion in the LBD S2 segment (*GRIN2C* (f,g), Table 4 and Fig. S22). The junctions defining this potential GluN2C-b isoform (1254 aa) were present in several human datasets at low-to-moderate levels (up to 21% abundance). However, in chimpanzee and macaque, we found the corresponding junctions to minor extent (Fig. S22B), and in rat and mouse, no homologous sequences exist. Apart from these isoforms, several other junctions appear to result in non-functional variants (*GRIN2C* (c), (d), (e), (h), and (i)) which are mostly subject to NMD (Table 4). We did not detect reads pointing to an alternative donor site reported for rat GluN2C [144]; also previously reported splice events for human cerebellar GluN2C [145] were absent or not detected at significant levels (GluN2C-3 had a global abundance of 4%; Table S4). However, a large number of rare events were detected for GluN2C (Table S4).

The GluN2D subunit is known to be expressed at lower levels than the other GluN2 subunits [39, 141, 146]. The read numbers for *GRIN2D* were rather low (Fig. 1 and Fig. S7) and we did not detect any clearly relevant alternative splice events (for rare events, see Table S4). An exitron (−82 nt) has been reported in the last exon of rat [141], but no splice site consensus sequences are present in rodents or primates (Fig. S23A).

GluN3A and GluN3B subunits confer special signaling properties to NMDA receptors [17, 18]. For *GRIN3A,* we identified all canonical junctions at reasonable levels (Fig. S7; [147, 148]). However, we did not identify any reads that would correspond to the GluN3A-long isoform that has been found in rat and mouse but not in humans [149–151]. This isoform is characterized by 20 aa insertion in the CTD, which results from an alternative splice acceptor site that causes a 5′ extension of the last exon. A more detailed analysis showed that the corresponding splice acceptor site is missing in humans and that the corresponding region is not conserved between primates and rodents (Fig. S23B).

Despite very low expression in the CNS (Fig. 1B), we detected all canonical *GRIN3B* junctions (Fig. S7; [152–155]). However, given the low transcript coverage, we cannot comment on alternative splicing (cf. Table S4). Several alternative isoforms have been isolated from rat developing white matter [156] and a polymorphic poly(Q) stretch appears to be present in human exon 9 [155].

### De novo identification of RNA editing events in iGluR transcripts

**Fig. 5:**
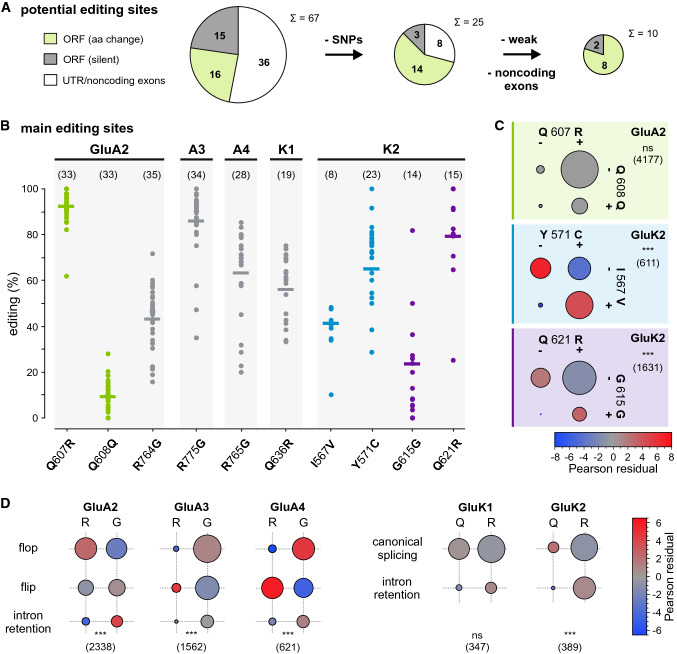
RNA editing in iGluRs and correlation with splicing. **A** Detection of single-nucleotide mismatches that report on potential A-to-I or C-to-U editing events (for details see Methods and Fig. S24). **B** Abundance of the ten most frequent events in different datasets (mean indicated by bars; (*n*) number of shown datasets (≥ 40 reads); see also Fig. S26). **C** Co-editing of sites within single-read distance. The circle sizes represent the read numbers obtained with editing-specific queries (see Table S8). Statistical testing shows independence for GluA2 Q607R/Q608Q editing, but interdependence for GluK2 Y571C/I567V and Q621R/G615G editing. **D** Relation between RNA editing and adjacent splice site usage. Statistical testing was performed with Pearson’s chi-squared test of independence (***p ≤ 0.0005; (*n*) number of total reads)

RNA-Seq data also contain direct information on single-nucleotide variations (Fig. 1A). Taking advantage of the high sequencing depth of the analyzed data and its origin from different human donors, we set up a strategy to identify potentially unknown editing sites (see Supplementary Methods; Figs. S1 and S24). For this, we considered frequent nucleotide mismatches that were present with ≥ 5% abundance in ≥ 30% of the datasets; still, 1220 mismatch positions were detected in exonic iGluR regions alone. We excluded poorly covered sites (see Supplementary Methods and Fig. S24) and obtained 67 mismatches, which could result from A-to-I or C-to-U editing in exonic regions (Fig. 5A and Table S7). Subsequent comparison to DNA-based data (dbSNP; NIH) revealed that 42 of these mismatches probably originate from widespread single-nucleotide polymorphisms (SNPs; Fig. S25). Some of the other mismatches showed different substitutions and/or were localized in non-coding regions (see Supplementary Methods). Eventually, our de novo identification resulted in ten mismatches in iGluR coding regions that could be unequivocally attributed to RNA editing (Fig. 5B), all of which had been described before.

We identified all eight major A-to-I iGluR editing events that are known to cause amino acid exchanges (Fig. 5B; Table S7; [8, 47]). Their editing frequencies are in good agreement with PCR-based quantifications (Fig. S26; [157, 158]). We found GluA2 Q/R editing to be the most abundant event (mean abundance 92.7%) followed by editing of the GluA3 R/G site (mean 86.3%). The least abundant, but still substantial editing events, result in silent amino acid exchanges: In GluA2, we found Q608Q editing, which is located + 4 nt of the Q607R site (mean 8.7%), and in GluK2, we found G615G editing −17 nt of the Q621R site (mean 23.5%). Due to their proximity to highly edited sites, these events may be considered secondary A-to-I editing events, which are controlled by the same editing site complementary sequence (ECS) [159, 160]. Other secondary events have been reported [158], but were detected with frequencies < 5%, such as GluA2-4 L/L editing −1 nt of the R/G site, as well as GluK1 G630G and GluK2 M620V editing (Table S7C). We also detected substantial editing of the intronic GluA2 editing hotspots + 60 and + 262 nt of the Q/R site [46], but, unlike in rodents no editing of adenosines at position + 263 and + 264.

In addition, we identified one exonic iGluR mismatch that could be caused by C-to-U editing, GluK1-d A870V (Fig. S25BC). However, this position is located in an alternative exon with poor coverage, the same substitution is encoded by a rare SNP, and the extent of C-to-U editing in the brain remains unclear [161, 162].

### Correlations between RNA editing events and editing and splicing

We continued by investigating co-editing of sites that occur within read length. Using specific sequence queries (Table S8), we found that in GluA2, where Q608Q editing occurs less frequently (overall 15.4% frequency) than Q607R editing (overall 95.9%), the four combinations still show relative abundances, as they would be expected for independent editing of the two sites (Pearson’s chi-squared test of independence, *p* > 0.05; Fig. 5C). For GluK2 Y571C/I567V, a different behavior is seen, since reads only edited to I567V are strongly underrepresented (overall 1.2%) compared to the other three combinations, which occur with similar frequencies (25–44%), i.e., editing at these sites does not occur independently (*p* ≤ 0.0005). The same is true for GluK2 Q621R/G615G, where reads only edited at the G615G site are strongly underrepresented (overall < 1%) and reads edited at only the Q621R site are the largest class (67%).

Different mechanistic interpretations could explain this behavior. Apparently independent editing, like in GluA2, would be observed, if adenosine deaminase binding is not rate limiting, or, if different ADAR activities (ADAR1/ADAR2) are involved. In contrast, a clear correlation, as seen for GluK2, where G615G editing increases with Q621R editing, would be expected, if the presence or recruitment of deaminase is limiting, but deamination of the two sites proceeds with different efficiency. However, also more complex regulatory mechanisms may be at play, since also editing of more distant sites [158] and within single cells [38] has been reported.

We also analyzed the relation between editing and close-by splicing sites, such as editing of the R/G sites in GluA2, GluA3, and GluA4, which are located −2 nt of the splice donor sites that mediate splicing to either the flop or flip exon [43]. Using sequence-specific queries, we investigated the interdependency of R/G editing and flip/flop splicing or intron retention (Fig. 5D; Table S8). Different patterns are seen for the three AMPAR subtypes: In GluA2, all R/G-flop/flip combinations are present at similar levels (overall 16.8–33.6%; |*r*|≤ 2.4). In GluA3, transcripts are mostly edited to G, but the R-flop combination remains particularly underrepresented (|*r*|≤ 4.7). In GluA4, which shows the strongest interdependence of editing and splicing, R-flop is again underrepresented, and G-flop is clearly overrepresented (|*r*|≤ 6.2). Similar observations have been reported in other human studies [157, 158]. However, it remains unclear, whether there is a mechanistic link between R/G editing and flip/flop splicing [50, 158, 163, 164], or whether these effects arise from cell type-specific effects, e.g., enrichment of certain splicing factors and deaminases [43]. From a functional perspective, it is interesting to note that in the case of GluA2, the effects of R/G editing on the desensitization kinetics are more pronounced in the flop isoform than in the flip isoform [43, 80, 165].

In GluK1 and GluK2, the Q/R editing sites are close to the 3′ exon boundary (−6 nt), which allowed us to test, whether editing is coupled to splicing or intron retention (Fig. 5D; Table S8). In GluK1, Q/R editing (overall 64.6%) and intron retention (overall 12.4%) appear to be rather independent, as confirmed by Pearson’s chi-squared test (*p* ≥ 0.05). In GluK2, Q/R editing (overall 90.2%) and intron retention (overall 37.8%) are more frequent, but unedited reads in combination with intron retention are clearly underrepresented (1.0%; |*r*|≤ 2.7) compared to the other combinations (8.7–53.5%). The higher fraction of edited transcripts still containing the subsequent intron could be explained by the extended presence of the intronic ECS, which may result in more complete editing. This effect has recently been also found in a systematic analysis of mouse brain pre-mRNAs, including *GRIA2*, *GRIA3*, *GRIK1,* and *GRIK2* [166]. In the case of GluA2 Q/R editing, an even stronger control mechanism seems to exist. There, splicing can only proceed after editing, which may be controlled by the involvement of intronic editing hotspots [46, 164, 167, 168]. This ensures the low Ca^2+^ permeability of most adult AMPA receptors, which is critical for proper nervous system function [45, 47, 48, 169].

## Discussion

iGluR splicing and editing are important mechanisms for fine-tuning synaptic function. In the case of iGluRs, most isoforms were identified by transcript cloning and most work has focused on rodents, although primate-specific splicing is well documented. In the last decade, RNA-Seq emerged as a powerful approach to analyze posttranscriptional modifications, such as alternative splicing and RNA editing, in a more global manner.

Here, we generated a first inventory of iGluR splicing in human brain by analyzing the vast amount of information provided by two large-scale RNA-Seq projects. We opted for a de novo identification approach and focused our analysis on junction-spanning reads (Fig. 1A), which provide the most direct experimental evidence for splice events. We then analyzed this information in the context of canonical iGluR transcripts and used abundance criteria to identify potentially relevant isoforms (Tables 1–4). Given the excellent coverage of the canonical transcripts (with the exception of *GRIN3B*; Fig. 1B and Fig. S7), we are able to provide relative abundance estimates for all individual splice events without having to rely on statistical most-likelihood approaches [65], which also enabled us to assess less frequent events. Compared to database information that is derived by automated pipelines, we provide manual evaluation, consistent annotations, and consistent abundance information that is based on a defined set of data. For the analysis of individual events, we integrated coverage track information, cross-species comparisons, and other available experimental data.

Besides the canonical iGluR junctions, we detected 865 alternative iGluR junctions (Table S4A), most of them occurring at low abundance (Fig. 2 and Fig. S5). The majority of these low-abundance events may result from erroneous splicing. Whole-genome RNA-Seq studies suggest an average error rate of ~ 1% per splice event and a huge number of potential splice sites as well as transcriptional noise [73, 170]. We thus only considered splice events as potentially relevant, if they were covered with ≥ 35 reads, and, if they had either an overall (global) abundance of ≥ 0.05 or were clearly enriched in individual datasets (maximum local abundance ≥ 0.15) compared to the canonical splice event. Still, not all events meeting these criteria may play a physiological role in human brain; vice versa, we cannot exclude that rare junctions may play a role in specific cell types, developmental stages, or disease states.

Based on the high coverage of the canonical junctions and saturation criteria (Fig. S8), we assume that we have identified all major splicing events within the adult human brain datasets. Our approach was not adapted for the detection of microexons and intron retention, which requires more specialized analysis techniques and datasets, respectively [166, 171–173].

Besides confirming existing data on most described splicing isoforms, our study provides relevant new insight: First, we obtained consistent abundance estimates for all splice events. We found that some previously reported isoforms occur at overall negligible amounts in human brain (e.g., GluK1-a and GluK5-b), whereas some other isoforms that have received little attention so far may occur at rather high abundance (e.g., GluA4-short/long and GluK1-1). For GluN1 C-terminal splicing, which has been well characterized and which plays important physiological roles (e.g., [11, 28, 125, 132]), we found a correlation between the two C-terminal splice events, which results in lower abundances of the GluN1-2 and GluN1-3 isoforms compared to the GluN1-4 and GluN1-1 isoforms, respectively (Fig. 4).

Second, we found a number of examples, where splicing is not conserved across species. For instance, the GluK3-b or GluN3A-long isoforms [112, 149, 150] are present in rodents but not primates (Figs. S15 and S23B). In contrast, the identified GluN2A-short (see also [139]) and GluN2C-b isoforms appear to be primate-specific (Figs. S20 and S22).

Third, we identified 19 new splice junctions, which we classified as relevant, in addition to 46 known alternative splice junctions reported in Ensembl fulfilling the same criteria. The relevant alternative junctions encode 52 transcript variations (Tables 1–4), 19 of which cannot be found in Ensembl.

Around half of the alternative transcripts (25/52) that fulfilled our abundance criteria encompass truncated open reading frames; 14 of these transcripts satisfy the criteria for NMD [56]. Abundant but non-coding transcripts may originate from cryptic splice sites and/or may have regulatory functions [1]. NMD plays an important role in eliminating these transcripts and may for instance ensure proper splicing of mutually exclusive exons [174–176]. This could also be the case for AMPA receptor flip/flop splicing, where we detected some erroneous exon combinations at levels up to 12% (Table 1 and Table S4), although these transcripts should be removed by NMD. Notably, no other regulatory mechanisms, such as branch point positioning [177], have been described to ensure mutually exclusive AMPA receptor flip/flop splicing.

Importantly, we discuss a number of events that appear to encode hitherto undescribed iGluR isoforms. This includes a highly expressed transcript that apparently encodes a GluA4-ATD, which may be secreted due to the presence of a signal peptide. We further confirmed this transcript by RT-PCR and, by searching available human proteome data, found evidence for expression of this unusual protein in human brain (Fig. S12). Furthermore, based on our de novo identification, we identified the first isoform of the delta receptor GluD1, GluD1-b, which carries an alternative CTD (Fig. 3). Verification by RT-PCR shows that the corresponding exon is present in ~ 15% of the transcripts (Fig. S18). Both, the GluA4-ATD and the GluD1-b isoform, are conserved across species. Novel, but less frequent, isoforms may also exist for GluK4 (GluK4-2, Fig. S16) and GluN2C (GluN2C-b, Fig. S22). Further studies are required to characterize these new potential isoforms, in particular on the protein level and with respect to physiological function. In other cases, we identified alternative UTRs (Tables 1–4; GluK2 Fig. S14A), most notably in GluN2C, where one newly identified 5′-exon (*GRIN2C* (a)) prevails over the previously described 5′-exon (Fig. S21).

We also investigated iGluR editing using human RNA-seq data (Fig. 5A,B). Our de novo identification approach captured all known major iGluR editing events, but we did not identify any novel, abundant A-to-I or C-to-U editing sites in exonic iGluR regions. The identification of novel low-frequency editing events was hampered by the widespread occurrence of SNPs, which in future studies might be circumvented by using matched genomic sequences from the same individuals. Similarly, the Investigation of editing in intronic regions and hyperediting (for instance in *Alu* elements) requires more specialized datasets and methods, but seems valuable as these events account for the vast majority of editing in humans [60, 178] and may have regulatory functions [179]. Interestingly, Pinto et al*.* found that only 59 editing sites are conserved across mammals, 20 of which belong to iGluR genes [60]. Another recent study suggests that the only physiologically essential editing event in mouse is GluA2 Q/R editing [180]. The role of C-to-U editing in the human brain remains elusive despite moderate editing of glycine receptor GlyRα3 [161, 162].

Finally, we used single-read information to analyze the co-occurrence of close-by editing and editing/splicing events, and in many cases, these events do not appear to occur independently (Fig. 5C,D). However, the interpretation of these results remains difficult and even for similar events, such as the relation between R/G editing and flip/flop-splicing, different results were obtained for GluA2, GluA3, and GluA4, which is in line with earlier reports [157, 158]. Besides mechanistic explanations (e.g., [50, 158, 164]), cell type-specific variations may account for this effect [38, 43]. Sequencing of long reads from single cells may provide the means to better understand the complex interplay between splicing and editing.

## Conclusions

Our study gives a first comprehensive overview of iGluR splicing and editing in the human brain and provides the impetus for further research. Future studies may focus on the iGluR splicing landscape in different brain regions, changes during development and disease, or its modulation by neuronal activity. Particularly large differences in iGluR expression and splicing are expected to be seen between different cell types, as for instance indicated by recent work on different neuronal populations from mouse brain (e.g., [37, 181]). In humans, similar information may become accessible by single-cell transcriptome analysis in combination with marker genes [182, 183]. Much further work will be necessary to address the functional consequences of splicing, not only on the level of the proteins, but also with respect to CNS function. Our study, which summarizes known and newly identified splice events, provides a framework for these investigations.

## Availability of data and code

We analyzed publicly available datasets (Sequence Read Archive (SRA, NIH); for accession numbers, see Table S1 and Table S6). All data obtained in this study are reported in Table S2-8. Nucleotide sequence data (new reported transcript assemblies) are available in the Third Party Annotation Section of the DDBJ/ENA/GenBank databases (NIH), see Table S9. Custom computer code used in this study can be made available upon request.

## Supplementary Information

Below is the link to the electronic supplementary material.

Supplementary file1 (PDF 15009 KB)

Supplementary file2 (XLS 123 KB)

Supplementary file3 (XLSX 575 KB)

Supplementary file4 (XLS 344 KB)

Supplementary file5 (XLSX 55 KB)

Supplementary file6 (XLSX 378 KB)

Supplementary file7 (XLSX 25 KB)
