# Supplementary material for: Splicing and editing of ionotropic glutamate receptors: a comprehensive analysis based on human RNA-Seq data
Source: Cell Mol Life Sci. 2021 Jun 8;78(14):5605–30. doi: 10.1007/s00018-021-03865-z (PMC8257547; doi:10.1007/s00018-021-03865-z)
Supplement: Supplementary file 1 — Supplementary file1 (PDF 15009 KB) [file 18_2021_3865_MOESM1_ESM.pdf]

Supplementary Information

Splicing and editing of ionotropic glutamate receptors: a comprehensive analysis based on human RNA-Seq data

Robin Herbrechter, Nadine Hube, Raoul Buchholz and Andreas Reiner *

Department of Biology and Biotechnology, Ruhr University Bochum,

***Corresponding author:**
Andreas Reiner
andreas.reiner@rub.de

This file includes:

**Supplementary Figures Overview**	p. 2
**Supplementary Figures S1-26**	p. 3
**Supplementary Tables Overview**	p. 33
**Supplementary Tables S1-2, S9**	p. 34
**Supplementary Methods**	p. 37
**Supplementary References**	p. 43

Supplementary Figures Overview

Fig. S1 Workflow: Splicing and editing analysis of human RNA-Seq data p. 3

Fig. S2 Quality control of analyzed datasets p. 4

Fig. S3 iGluR abundance across different datasets p. 5

Fig. S4 *De novo* identification of splice junctions and classification of splice events p. 6

Fig. S5 Read coverage of different iGluR junction types p. 7

Fig. S6 Analysis of splice donor and acceptor sites p. 8

Fig. S7 Abundance of canonical iGluR splice junctions p. 9

Fig. S8 Detection of iGluR junctions during dataset analysis p. 10

Fig. S9 Abundance of alternative splice junctions in individual datasets p. 11

Fig. S10 AMPA receptor flip/flop splicing and additional cassette exons in *GRIA2* and *GRIA4* p. 12

Fig. S11 AMPA receptor C-terminal splicing p. 13

Fig. S12 GluA4-ATD isoform p. 15

Fig. S13 Alternative GluK1 isoforms p. 17

Fig. S14 *GRIK2* alternative 5’-UTR and C-terminal splicing p. 18

Fig. S15 *GRIK3* alternative splicing at the C-terminus p. 19

Fig. S16 Potential GluK4-2 isoform p. 20

Fig. S17 Potential GluK5-b isoform p. 22

Fig. S18 Additional characterization of the GluD1-b isoform p. 23

Fig. S19 GluN1 splicing in the C-terminal region p. 25

Fig. S20 Primate-specific GluN2A-short isoform p. 26

Fig. S21 *GRIN2C* alternative 5’-UTRs and alternative start codon in humans p. 27

Fig. S22 Potential primate-specific GluN2C-b isoform p. 28

Fig. S23 Non-conserved splice events in GluN2D and GluN3A p. 29

Fig. S24 *De novo* identification of potential RNA editing sites p. 30

Fig. S25 Comparison of frequent nucleotide mismatches to known SNPs p. 31

Fig. S26 Frequency of the main iGluR editing events in coding regions p. 32

Supplementary Figures S1-S26

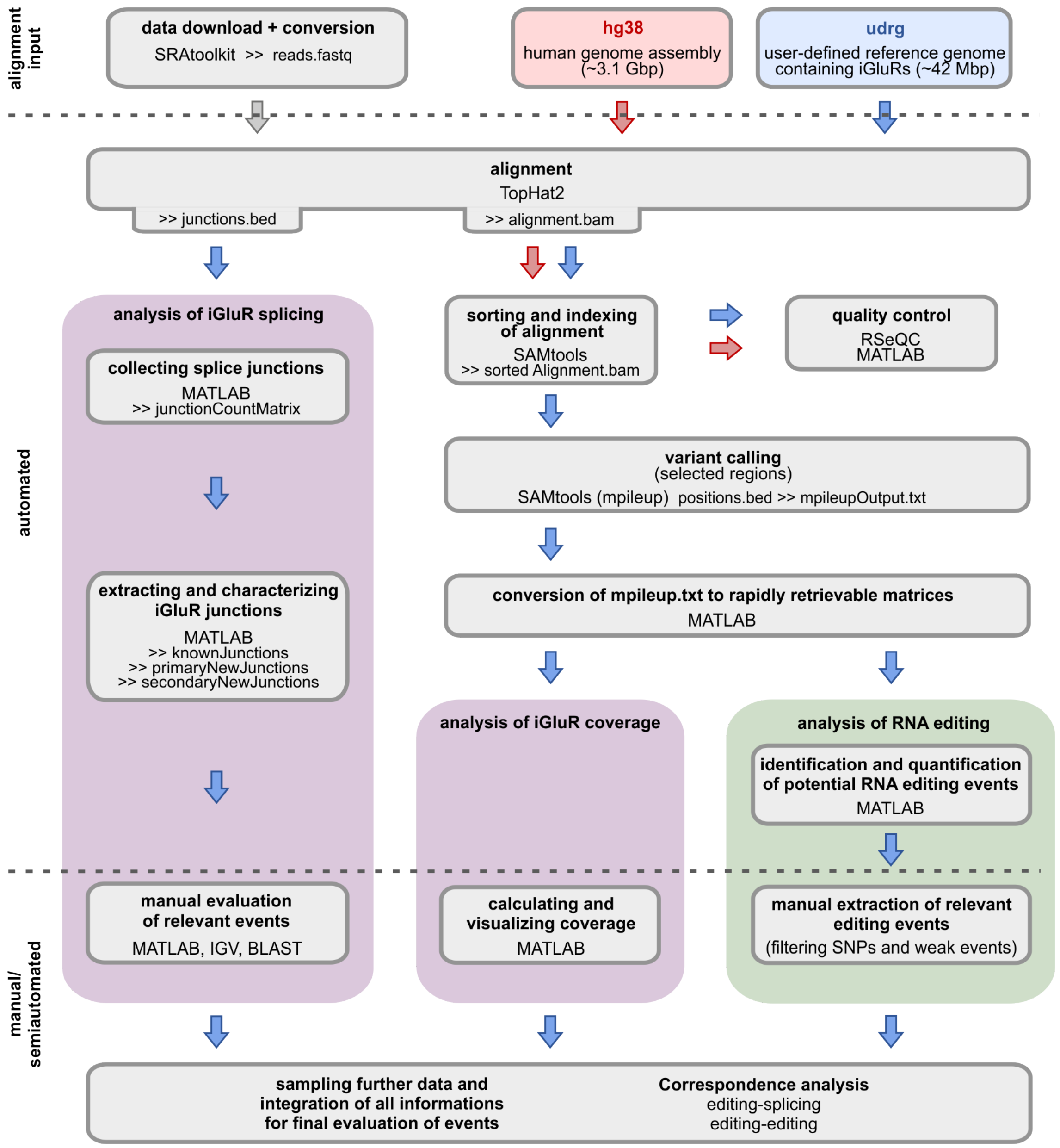

Figure S1. Workflow: Splicing and editing analysis of human RNA-Seq data. The investigated RNA-Seq reads were aligned to the human genome assembly hg38 (red) and to a user-defined reference genome (udrg; blue) using TopHat2. Analysis of alternative splicing focused on the detection and classification of junction-spanning reads (left). The overall alignments were used for quality control and variant calling, which later served to analyze transcript coverages (center) and to identify single nucleotide mismatches that may correspond to editing events (right). For details on the individual steps see **Supplementary Methods**.

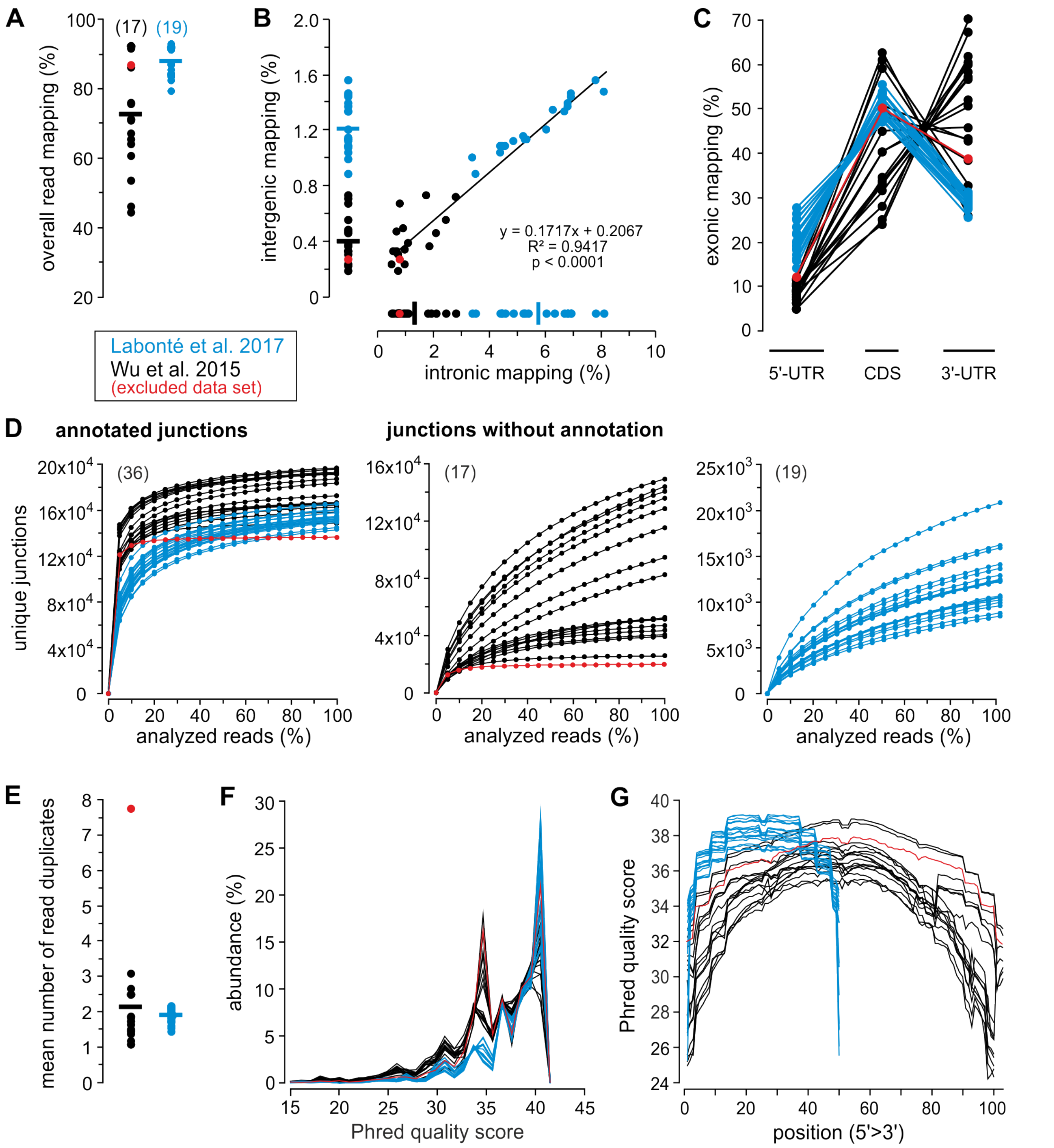

Figure S2. Quality control of analyzed datasets. In total we analyzed 36 RNA-Seq datasets (see **Table S1**) from two human transcriptome studies (Labonté *et al.*, 2017; Wu *et al.*, 2015). The two studies differ in sample preparation and read length (for details see **Supplementary Methods**). Data from the study of Wu *et al.* are shown in black, data from the study of Labonté *et al.* in blue. One dataset was excluded due to over-amplification (red). **(A)** Overall read mapping rates of individual datasets after alignment to the hg38 reference genome. **(B)** Read mapping to intergenic and intronic regions. **(C)** Read mapping to exonic regions. **(D)** Detection of junction-spanning reads upon alignment to the hg38 reference genome. All datasets comprised 13.7-19.6·10^4 annotated junctions, which were detected rather early during analysis (left panel), and variable numbers of junctions without annotations (right panels **(E)** Mean number of read duplicates, which align to the canonical iGluR exons within the udrg. **(F)** Distribution of Phred quality scores of the reads aligning to the canonical iGluR exons (see **Supplementary Methods**). **(G)** Phred quality scores across read positions. Bars in panel (A, B) and (E) indicate means.

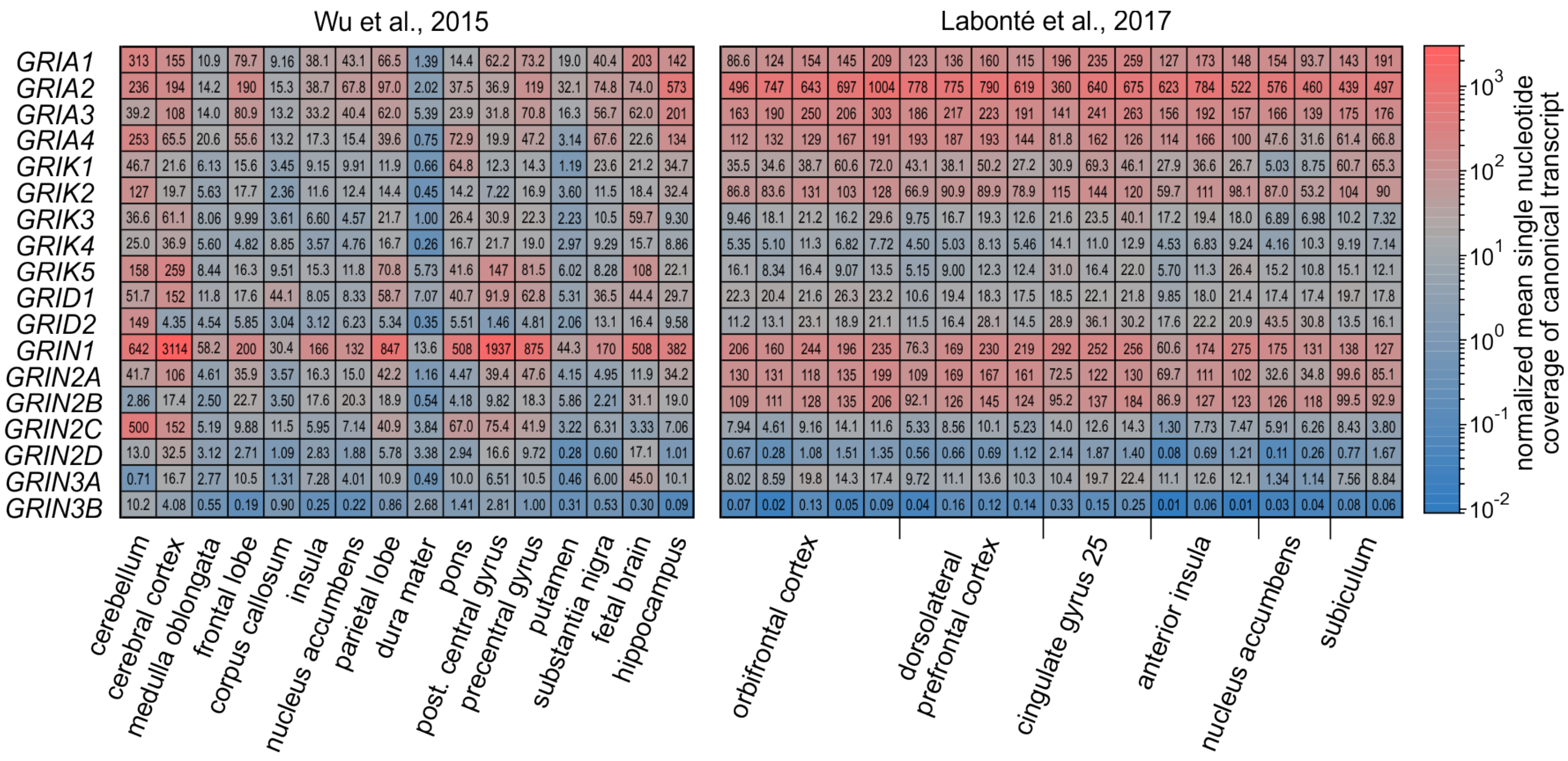

Figure S3. iGluR abundance across different datasets. Heat map showing the mean single nucleotide coverage of the canonical transcripts (**Table S2**) after normalization for dataset size (**Table S3**). The single nucleotide coverage was obtained from the udrg alignments using SAMtools mpileup function. The dataset size was measured by the number of bases that aligned to hg38 (see **Supplementary Methods**).

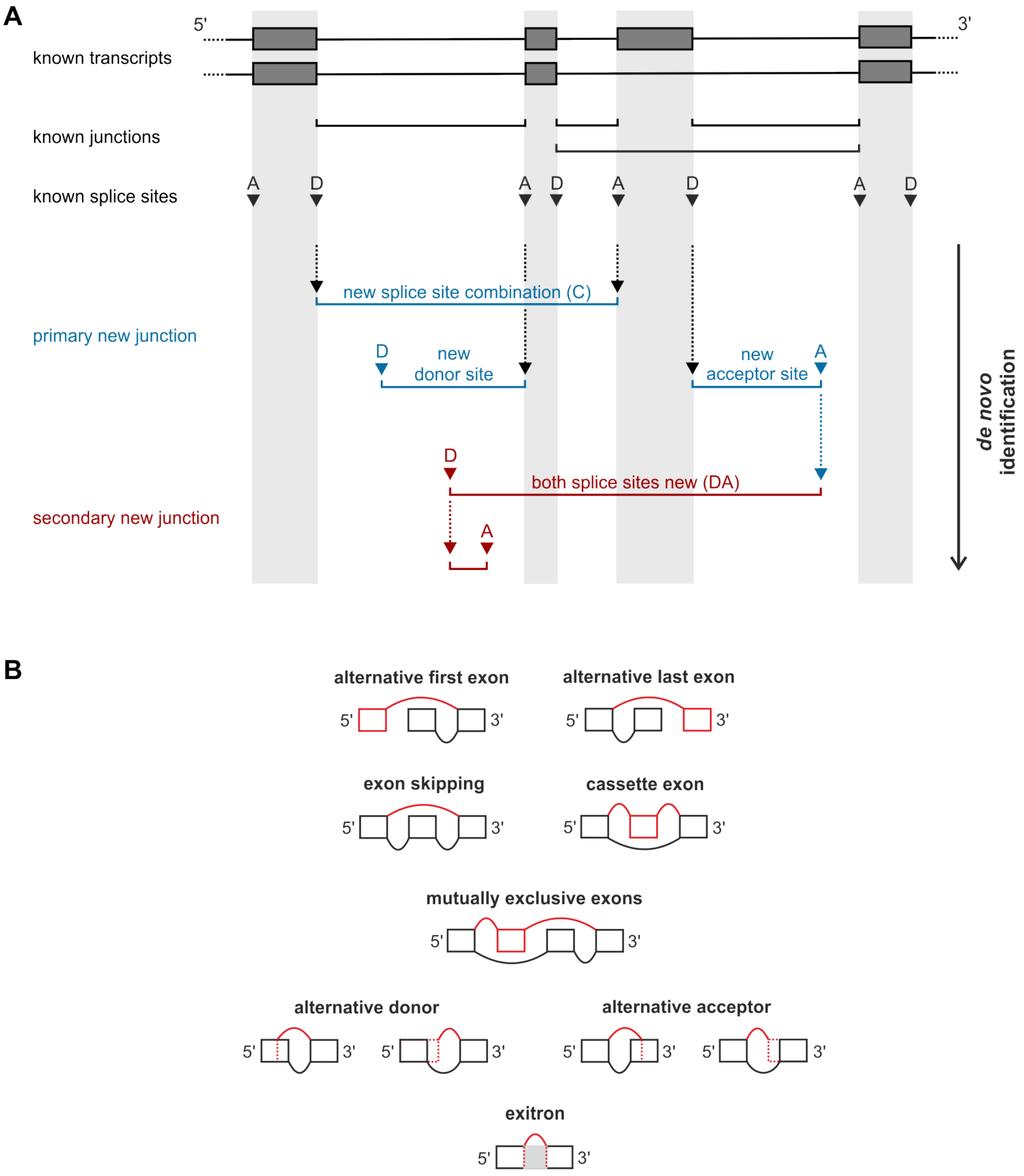

Figure S4. *De novo* identification of splice junctions and classification of alternative splice events. (A) Previously deposited iGluR transcripts (grey) were used to extract all known iGluR splice junctions and splice sites, which served as starting points for the identification of new iGluR splice junctions. Primary new junctions encompass at least one known splice site and are either a new combination, 'C', of two known splice sites, or they possess either a new splice donor site, 'D', or, a new acceptor site, 'A'. In subsequent steps, newly identified splice sites served as anchor points for the detection of secondary new junctions (red), i.e. junctions, which have no splice site in common with previously known transcripts. **(B)** Classification of alternative splice events (red). Exons are shown as boxes, splice junctions as lines.

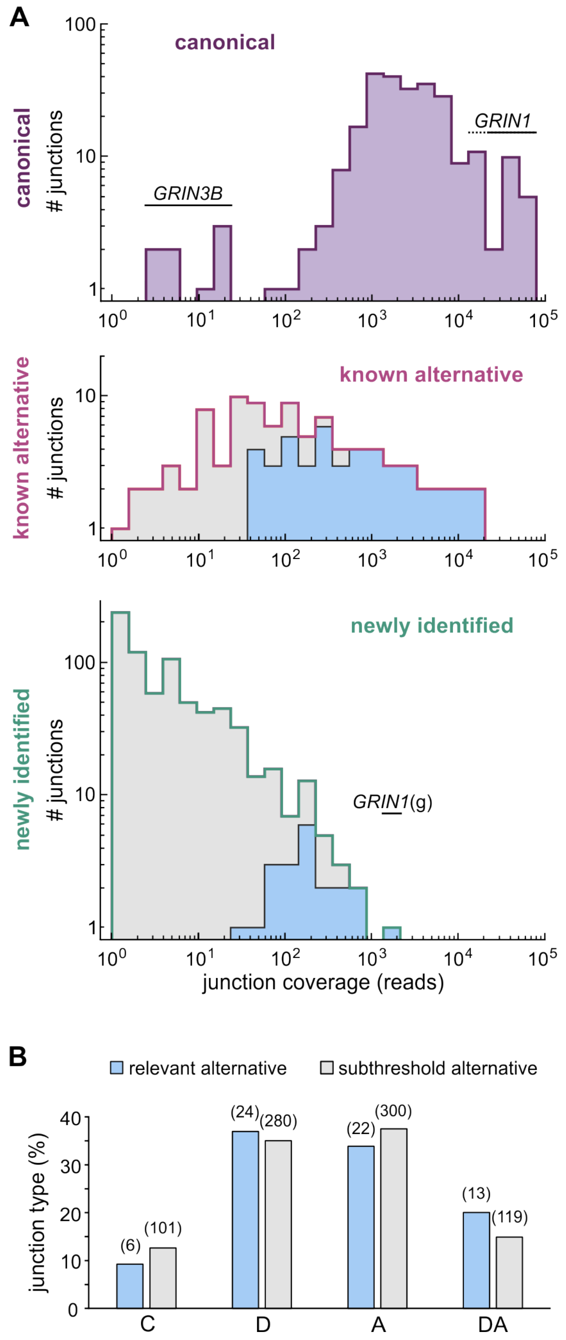

Figure S5. Read coverage of different iGluR junction types. (A) The histograms show distributions of total junction coverages for canonical (top), known alternative (center), and newly identified splice junctions (bottom). Junctions classified as relevant are highlighted in violet (canonical) and blue (alternative). For normalized data compare **Fig. 2**. **(B)** Relevant alternative junctions (blue) and low abundance junctions (grey) show a similar distribution of junction types ('C', alternative combination of canonical donor and acceptor sites; 'D', alternative donor site; 'A', alternative acceptor site; 'DA', alternative acceptor and donor sites).

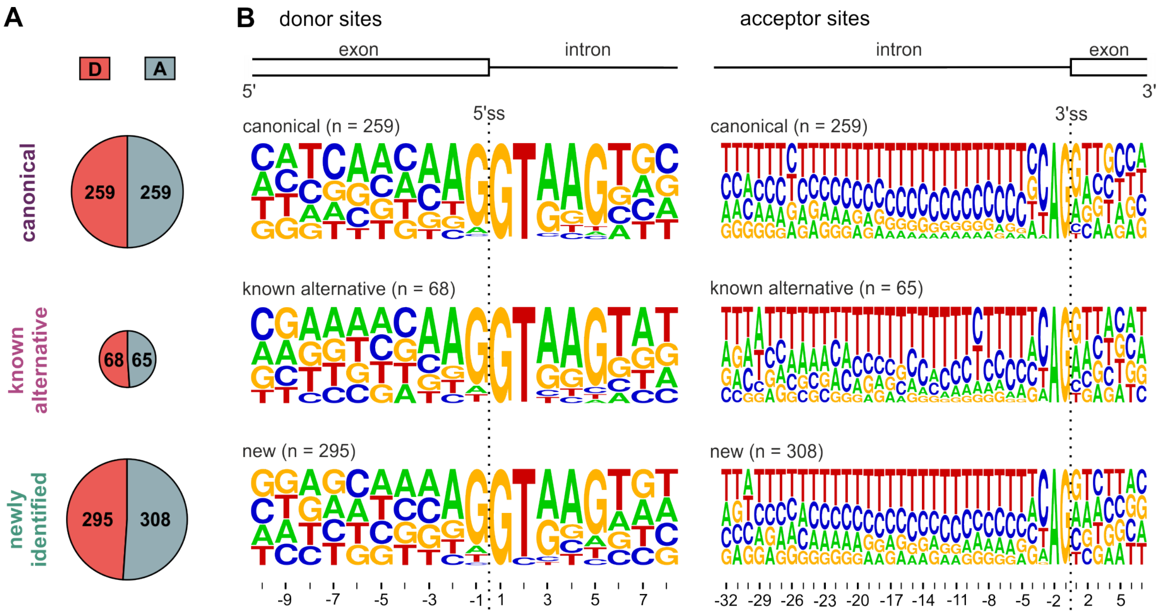

Figure S6. Analysis of splice donor and acceptor sites. (A) Number of donor, ‘D’, and acceptor, ‘A’, splice sites within the canonical, known alternative, and newly identified iGluR splice junctions. **(B)** All identified donor and acceptor splice sites show typical features of major spliceosome (U2) intron recognition sites (GT-AG, to a minor degree GC-AG) and a polypyrimidine tract at the acceptor site. This indicates that the identified reads report on *bona fide* splice junctions. Sequence logos were generated using WebLogo 2.8.2 (Crooks *et al.*, 2004).

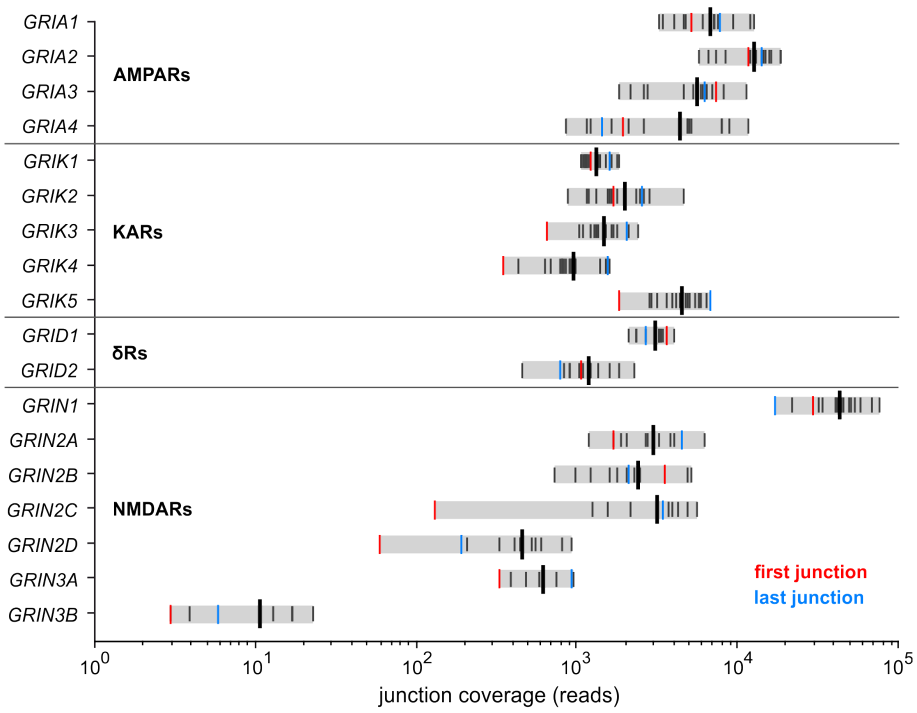

Figure S7. Abundance of canonical iGluR splice junctions. The total number of junction-spanning reads for all canonical iGluR junctions in the analyzed 35 datasets is shown (short bars). The first junction of the canonical transcript is shown in red (5'), the last junction in blue (3'). Longer bars show the corresponding mean.

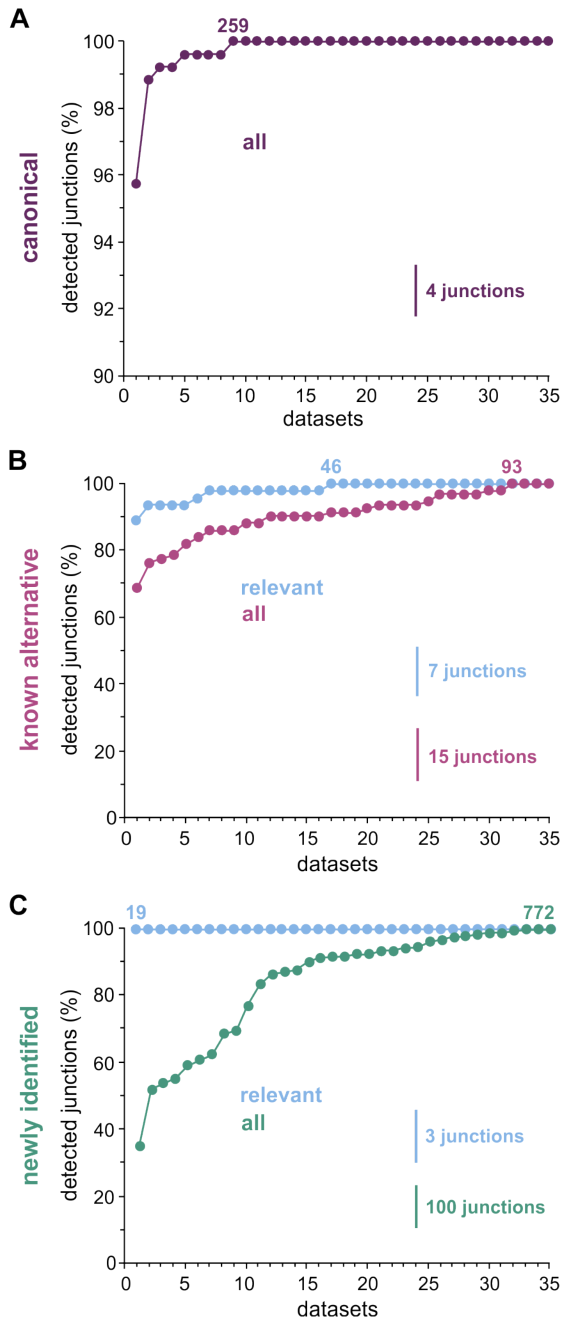

Figure S8. Detection of iGluR junctions during dataset analysis. (A) All 259 canonical iGluR junctions had been detected at least once after analyzing the first 9 datasets. **(B)** After analyzing 32 datasets, 93 of 120 known alternative junctions had been detected (purple). The 46 known alternative junctions that met our relevance criteria (blue) had been already detected at least once after analyzing half of the datasets. **(C)** In total, we identified 772 new junctions (green). The 19 new junctions that met our relevance criteria (blue) were already present in the first analyzed dataset. Datasets ordered as in **Table S1** (without brain dataset).

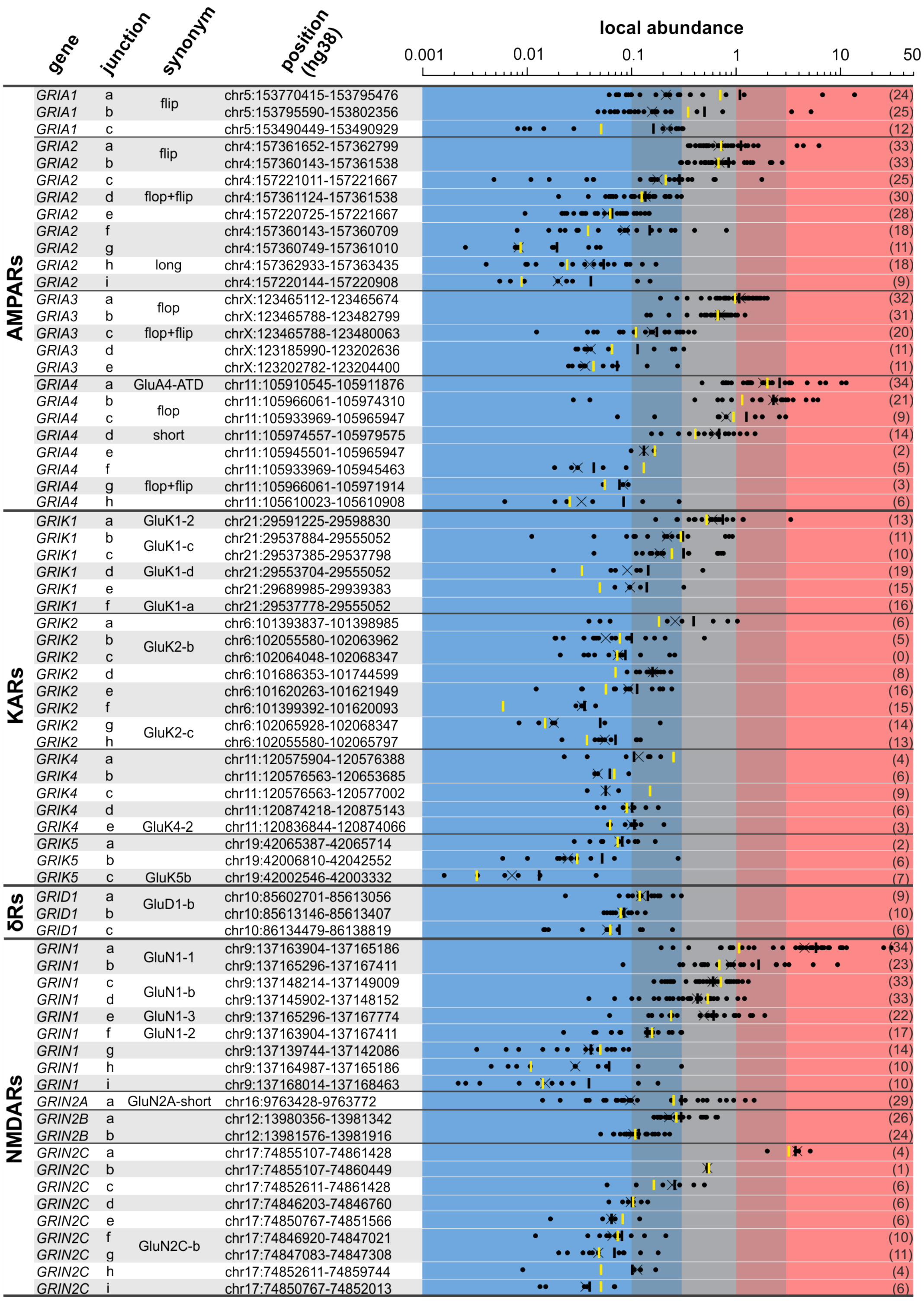

Figure S9. Abundance of alternative splice junctions in individual datasets. Data points show the relative abundance of the indicated junctions after normalization to the canonical junction(s) within individual datasets (local abundance; calculated for datasets with ≥ 40 junction-spanning reads, number of datasets given in parentheses). Black bars indicate the corresponding mean, crosses the median local abundance. For comparison, the global abundance is shown by yellow bars (**Table 1-4**). For details see **Supplementary Methods** and **Table S4**.

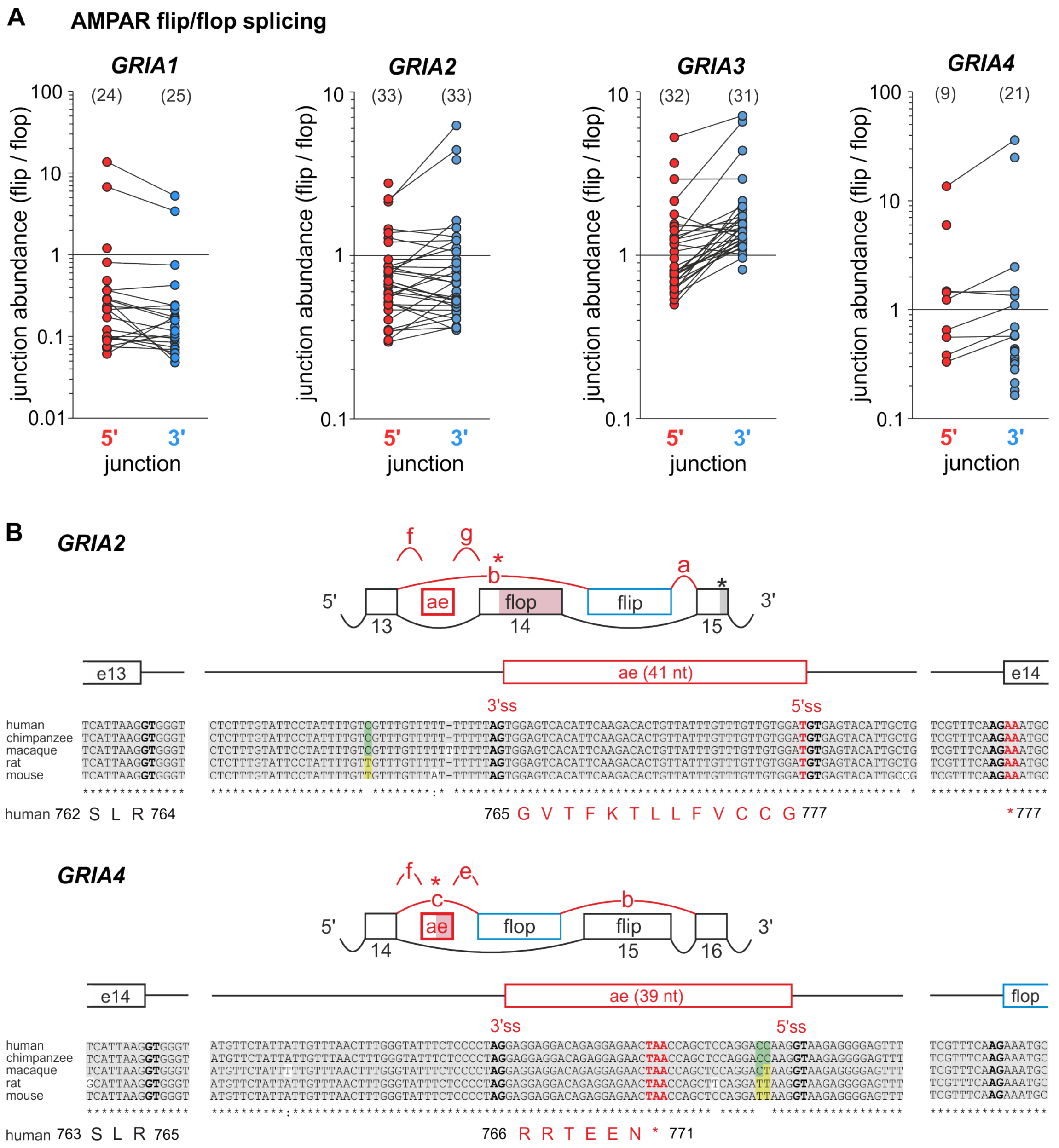

Figure S10. AMPA receptor flip/flop splicing and additional cassette exons in *GRIA2* and *GRIA4*. (A) Abundance of the 5’ (red) and 3’ (blue) junctions of the flip isoform normalized to the corresponding junctions of the flop isoform in single datasets. The number of analyzed datasets is given in parentheses (datasets with ≥ 40 junction-spanning reads). **(B)** Inclusion of cassette exons in *GRIA2* and *GRIA4*, which occurs with low (*GRIA2* (f-g)) to medium frequency (*GRIA4* (f-e)), see **Table 1**. The *GRIA2* exon (41 nt) introduces a frameshift and premature stop codon in the flop exon, which should mark the transcript for NMD. The *GRIA4* exon (39 nt) encodes a stop codon, which should also cause NMD. Both exons appear to be conserved between species and could have regulatory functions. For alignments see **Supplementary Methods**.

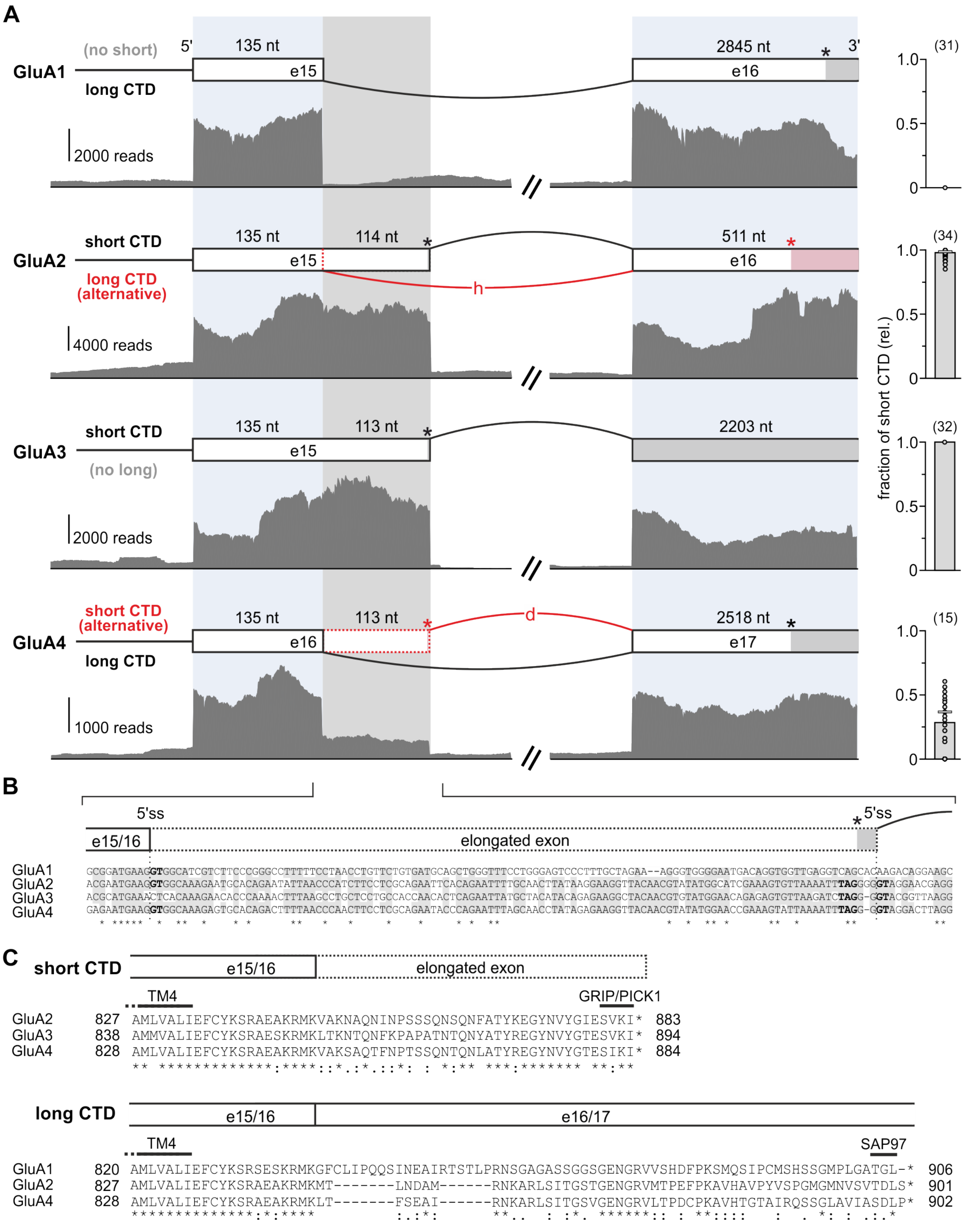

Figure S11. AMPA receptor C-terminal splicing. In AMPA receptors two different C-terminal splice variants exist, which are termed 'long' and 'short'. The short isoforms arise from prolongation of the penultimate exon (e15 in GluA2 and GluA3; e16 in GluA4), which introduces an early stop codon. **(A)** Coverage tracks showing the corresponding exonic and adjacent intronic regions (single nucleotide coverage over all 35 datasets). Canonical events are shown in

black, alternative splice events in red. Stop codon positions are indicated by asterisks. The boxes represent the global junction abundance. Single dots represent the local abundance in datasets with sufficient coverage (≥ 40 splice junction-spanning reads; number of datasets given in parentheses) and bars show the median local abundance. **(B)** Nucleotide alignment of the corresponding exon region in human GluA1-4. GluA1 has lost the second intron splice site (GT), and GluA3 the first intron splice site, which explains why GluA1 and GluA3 exist as only long and short isoforms, respectively. In GluA1, the exon extension is no longer conserved and the stop codon is lost. **(C)** Amino acid alignment of the short and long CTD sequences. The short isoforms of GluA2-4 contain type II PDZ binding motifs, which for instance mediate interactions with glutamate receptor-interacting protein (GRIP) (Tan *et al.*, 2015) and protein interacting with C kinase 1 (PICK1) (Dev *et al.*, 1999). GluA1-long contains a type I PDZ binding motif, which interacts with e.g. synapse-associated protein 97 (SAP97) (Leonard *et al.*, 1998). In GluA2 and GluA4-long this motif is masked by a C-terminal serine/proline residue (Coleman *et al.*, 2010).

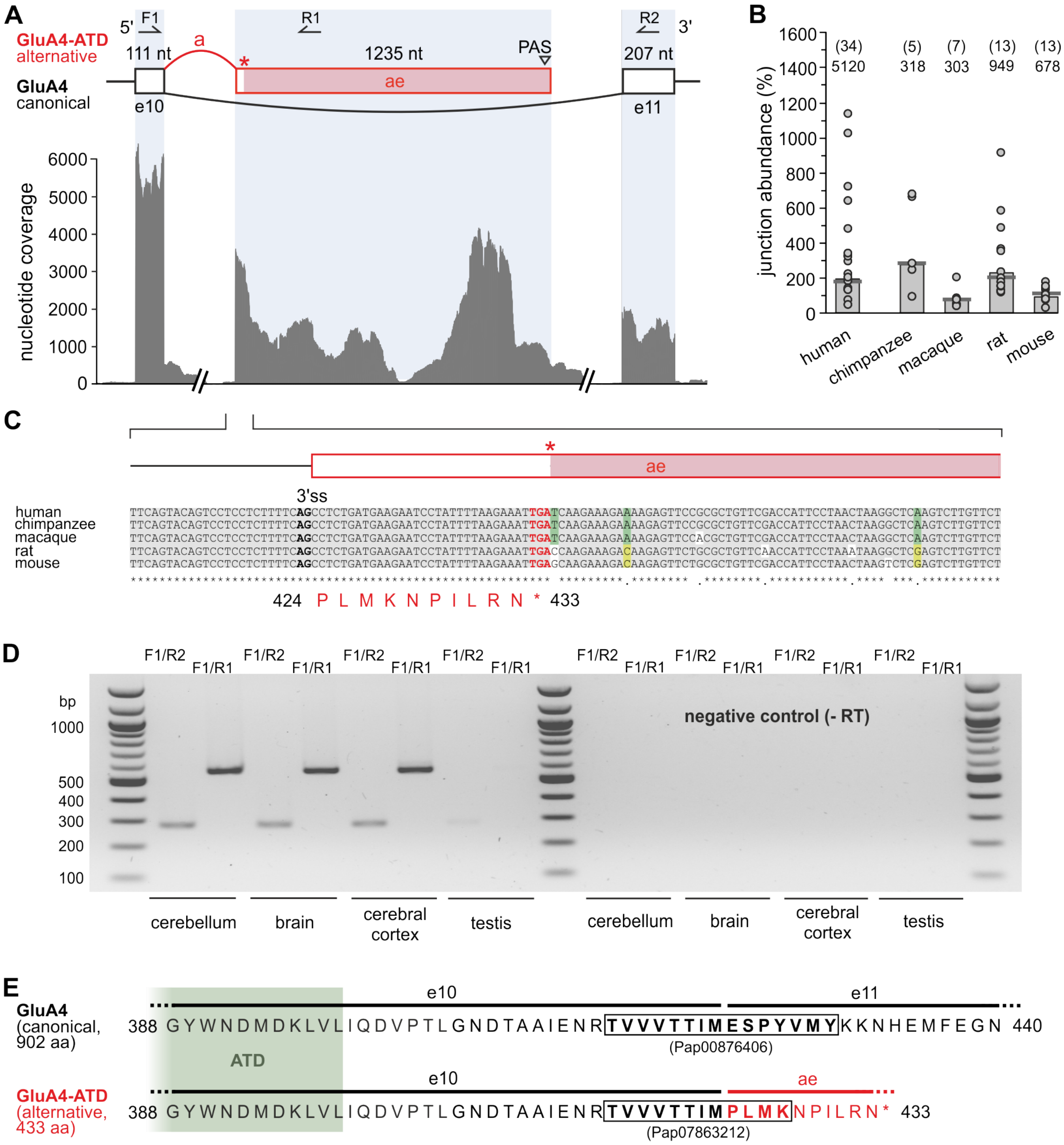

Figure S12. GluA4-ATD isoform. (A) Splicing from exon e10 to an alternative exon (ae) introduces an early stop codon (asterisk), which is followed by a 3'-UTR containing a polyadenylation signal (PAS). This event is characterized by the presence of a highly abundant splice junction, *GRIA4* (a) (**Table 1**). The alternative exon can be clearly distinguished in the coverage track (single nucleotide coverage over all 35 datasets). **(B)** Relative abundance of the alternative splice junction *GRIA4* (a) compared to the canonical junction in human datasets. The corresponding junction can also be found in chimpanzee, macaque, rat and mouse (abundance obtained by junction-specific BLAST queries; see **Supplementary Methods** and **Table S6**). The boxes represent the global junction abundance with the total number of alternative reads given on top. Single dots represent the local abundance in individual datasets with sufficient coverage (≥ 40 splice junction-spanning reads, analyzed datasets given in parentheses, means indicated by bars). **(C)** Nucleotide alignment of the alternative exon region shows high sequence

conservation across species. The splice acceptor site (AG) and stop codon are indicated in bold. **(D)** RT-PCR detection of canonical and GluA4-ATD transcripts in human RNA samples from cerebellum, brain, cerebral cortex, and testis (see **Supplementary Methods**). Exon-specific primers show the relative amounts of canonical splicing (F1/R2, expected product size: 278 bp) and GluA4-ATD (F1/R1, 565 bp). Bands are absent in negative controls without reverse transcriptase (-RT). **(E)** The splicing event results in a GluA4-ATD only protein (433 aa) with only a short amino acid stretch (35 aa) following the ATD. The last 10 aa are encoded by the alternative exon. The highlighted regions show unique peptides matching the canonical and alternative exon boundaries, which have been identified in a human proteome study (McKetney *et al.*, 2019) and thus confirm expression of this isoform on the protein level.

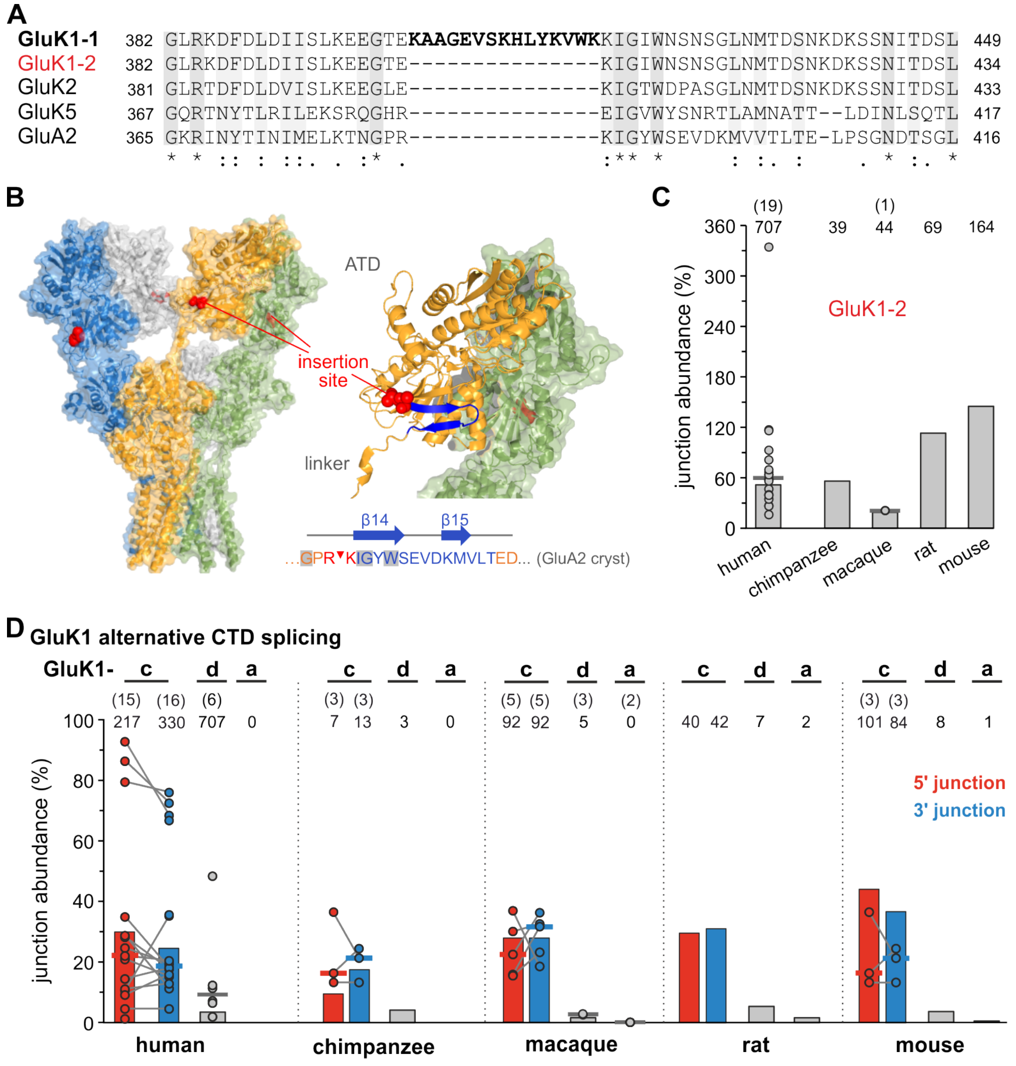

Figure S13. Alternative GluK1 isoforms. (A) The GluK1-1 isoform (canonical) carries a unique cassette exon (e9, 45 nt; **Table 2**), which results in 15 aa insertion towards the end of the ATD, after E401. **(B)** The insertion site is located at the bottom of the ATD, just before the *β*-hairpin 14/15 (R384-K385 in GluA2; figure based on PDB 3KG2 (Sobolevsky *et al.*, 2009)). The insertion is not present in other iGluR subunits. **(C)** Quantification of the alternative GluK1-2 junction (skipping of e9) shows that both isoforms occur with overall similar abundance in all investiagted species (junction-specific queries, see **Table S6**). A similar abundance of GluK1-1/2 has also been reported for cloned rat cDNAs (Bettler *et al.*, 1990). **(D)** Quantification of GluK1 C-terminal splice isoforms, GluK1-a to GluK1-d, in different species (normalized to the canonical GluK1-b event). The junctions corresponding to GluK1-c are detected frequently, GluK1-d rarely and GluK1-a is mostly absent (see also **Main Text**; for junction-specific queries, see **Table S6**). Boxes represent the global junction abundance with the total number of alternative reads given on top. Single dots represent the local abundance in individual datasets with sufficient coverage (≥ 40 splice junction-spanning reads, number of analyzed datasets given in parentheses, mean indicated as bars).

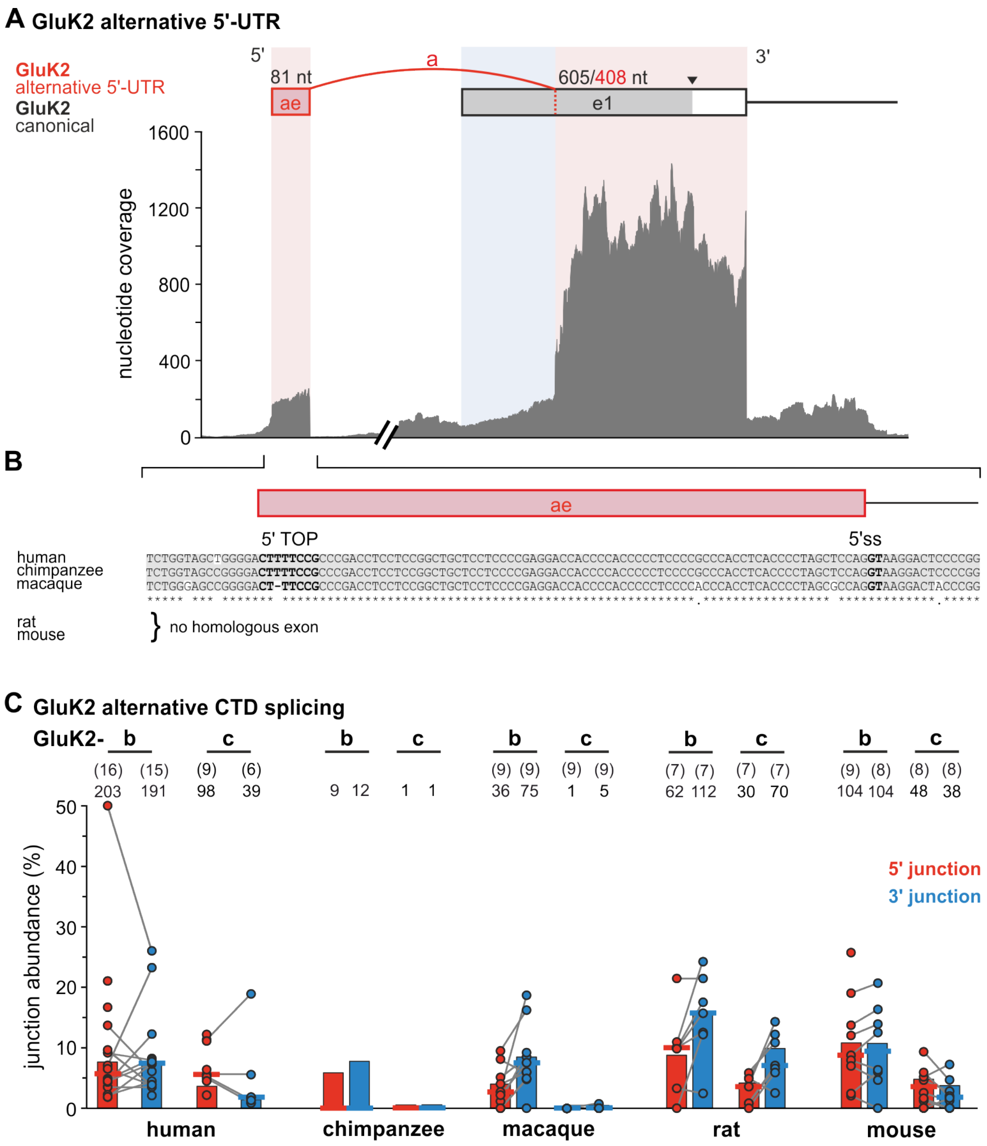

Figure S14. *GRIK2* alternative 5'-UTR and C-terminal splicing. (A) A newly identified splice junction, *GRIK2* (a), indicates the presence of a non-coding alternative exon (ae) in the 5'-UTR. This abundant junction involves an alternative splice acceptor site in canonical exon e1 (**Table 2**). The alternative exon is clearly defined in the coverage track (single nucleotide coverage over all 35 datasets) and contains a short 5'-terminal oligopyrimidine track (5'-TOP; predicted by RegRNA2.0). The corresponding splice event has been detected before (Zhawar *et al.*, 2010), but the 5'-exon appears to be shorter than reported. **(B)** The alternative exon is conserved in human, chimpanzee and macaque. The 5'-TOP track and the donor intron recognition site (GT) are indicated in bold. **(C)** Abundance of GluK2 C-terminal isoforms, GluK2-b to GluK2-c, normalized relative to the canonical GluK2-a junction (cf. **Table 2**). For junction-specific queries in other species, see **Table S6**. Boxes represent the global junction abundance with the total number of alternative reads given on top. Single dots represent the local abundance in individual datasets with sufficient coverage (≥ 40 splice junction-spanning reads, number of analyzed datasets given in parentheses, mean indicated as bars).

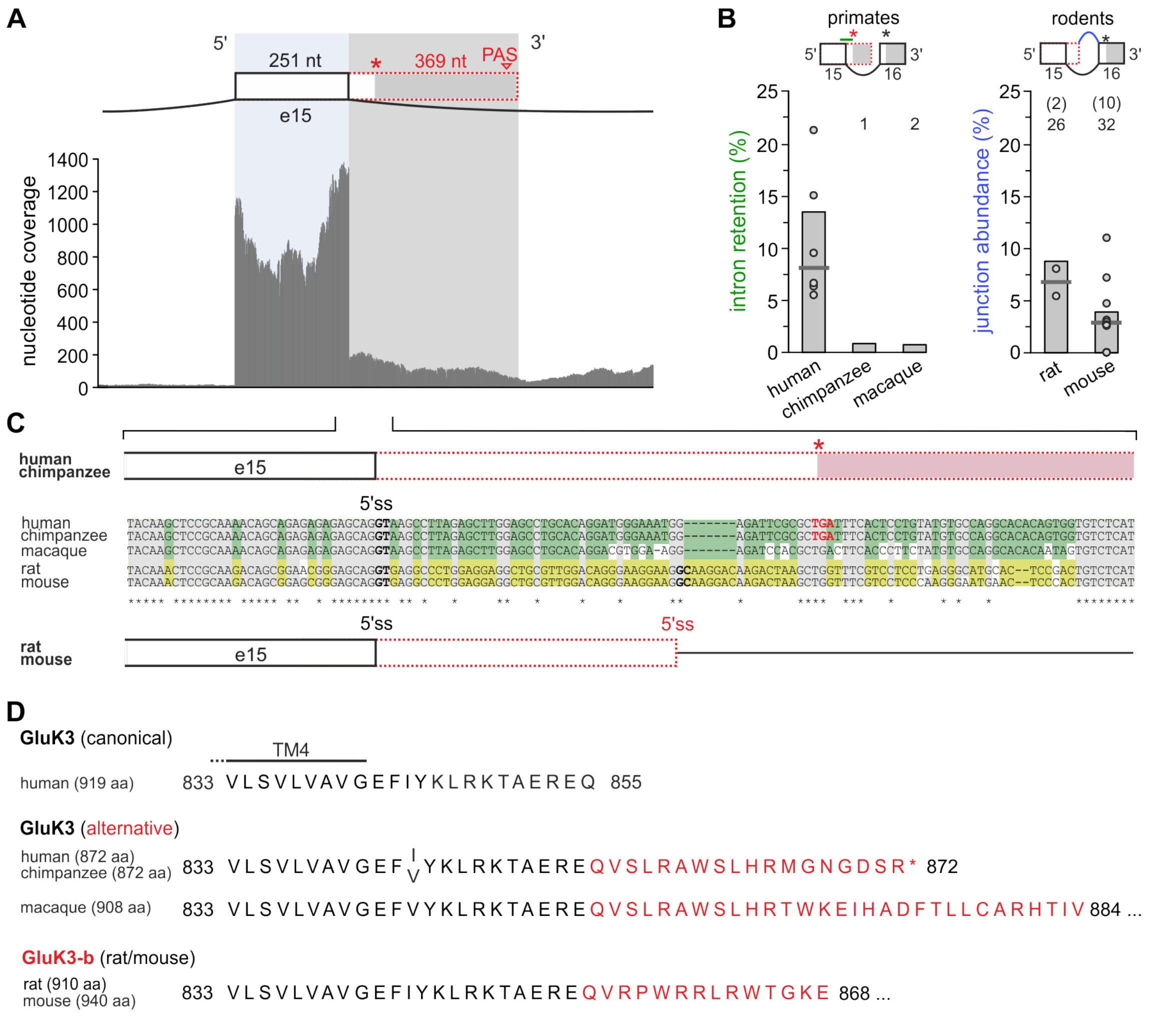

Figure S15. *GRIK3* alternative splicing at the C-terminus. For rat and mouse an alternative GluK3-b isoform has been described (Schiffer *et al.*, 1997), which results from an alternative splice donor site in the penultimate exon, e15. **(A)** A human Ensembl transcript (ENST00000373093.4) suggests prolongation of e15 (*GRIK3* (a)), but unlike in rodents, we did not identify a subsequent junction to the last canonical exon, e16. However, prolonged e15 would code for an early stop codon and also a polyadenylation site (PAS). The coverage track shows the single nucleotide coverage over all 35 datasets. **(B)** Analysis of the coverage track suggests that an extension beyond the canonical splice site may be present in ~14% of the human transcripts, but BLAST queries (**Table S6**) rarely show an extension in chimpanzee and macaque datasets. The junctions of the GluK3-b isoform in rat and mouse are detected with a global 9% and 4% abundance, respectively. The boxes represent the global junction abundance with the total number of alternative reads given on top. Single dots represent the abundance in individual datasets with sufficient coverage (≥ 40 splice junction-spanning reads, number of datasets given in parentheses) with the median shown as bar. **(C)** Nucleotide alignment of the 3'-regions of e15. The primate sequences lack the alternative splice donor site (GC) that is part of the described rodent GluK3-b isoform. **(D)** Amino acid sequences. The alternative primate sequence would not match the GluK3-b sequence in rat and mouse. In macaque the C-terminal sequence would differ from the human and chimpanzee sequence due to a frame shift.

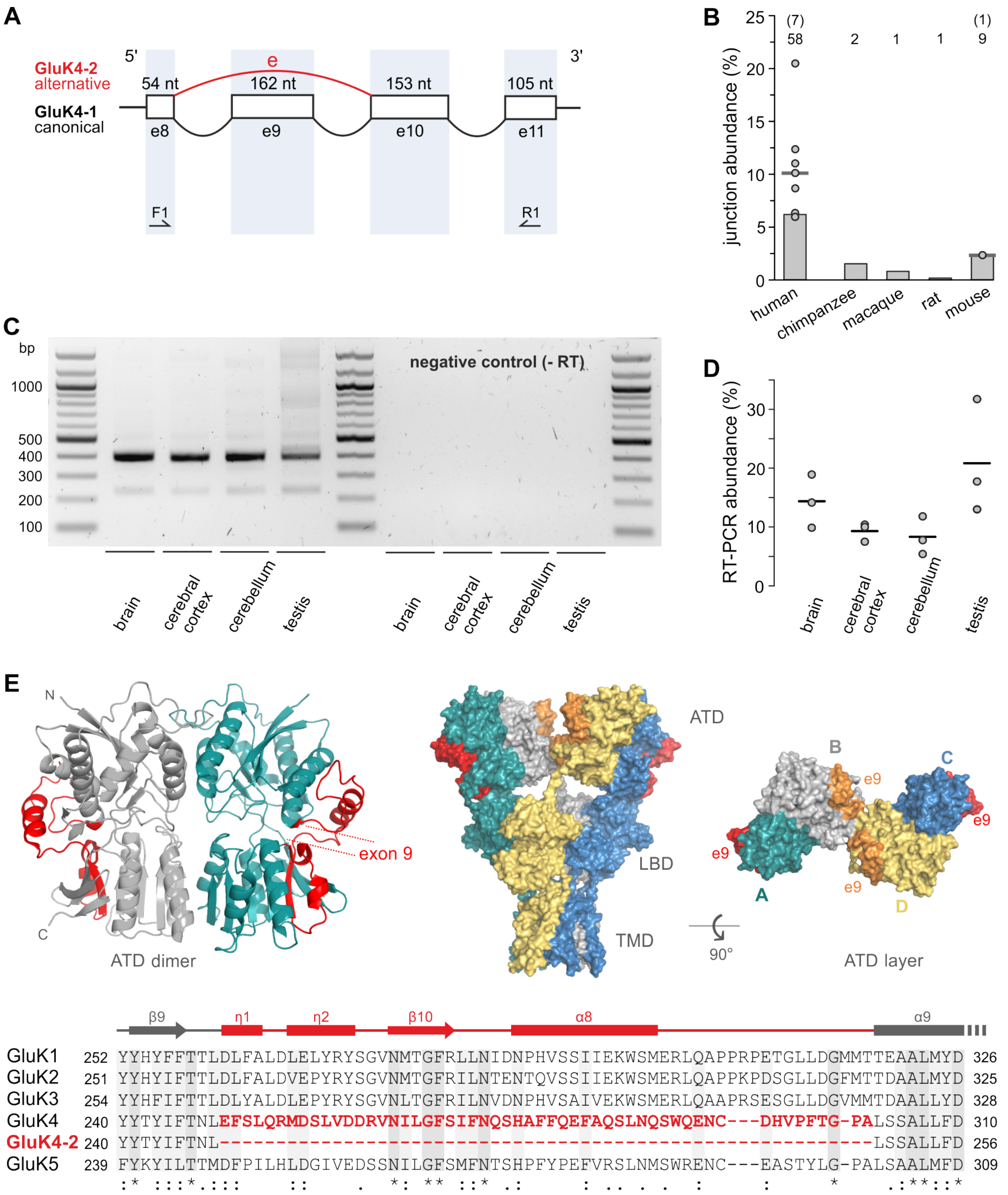

Figure S16. Potential GluK4-2 isoform. (A) Our *de novo* junction identification revealed skipping of canonical exon e9 (162 nt), i.e. an alternative combination of splice donor and acceptor sites (*GRIK4* (e), **Table 2**). **(B)** In humans this junction is detected with low-to-moderate frequency but junction-specific queries **(Table S6)** show that this event may play only a minor role in other species. Boxes represent the global junction abundance with the total number of alternative reads given on top. Single points represent the abundance in individual datasets with sufficient coverage (≥ 40 splice junction-spanning reads, number of datasets given in parentheses, median indicated as bars). **(C)** RT-PCR detection of GluK4-1/2 in human RNA samples from brain, cerebral cortex, cerebellum and testis (see **Supplementary Methods**). Exon-specific primers F1/R1 show the relative amounts of transcripts with and without exon 9

(expected product sizes: GluK4-1 396 bp; GluK4-2 234 bp). Bands are absent in negative controls without reverse transcriptase (-RT). **(D)** Relative amounts of GluK4-2 RT-PCR products in RNA from different human tissues (3 independent cDNA syntheses each). **(E)** The exon skipping results in a 54 amino acid deletion in the ATD region without causing a frame-shift (E249-A302 in GluK4). The structural model shows the potentially deleted region (red, exon 9) in the ATD dimer (left). The N- and C-termini of exon 9 are close-by, which may preserve the structural integrity of the ATD (G241-K294 in GluA2 ATD dimer; based on PDB 3H5V (Jin *et al.*, 2009)). In the full-length receptor (middle) the deleted GluK4-2 regions (red) would be expected to be facing outward (subunit A and C), while the subunits facing inward are likely occupied by GluK1-3 subunits in analogy to GluK2/GluK5 heteromers (Kumar *et al.*, 2011). The full-length structure, ATD layer structures (middle and right) and secondary structure annotations are based GluA2, PDB 3KG2 (Sobolevsky *et al.*, 2009).

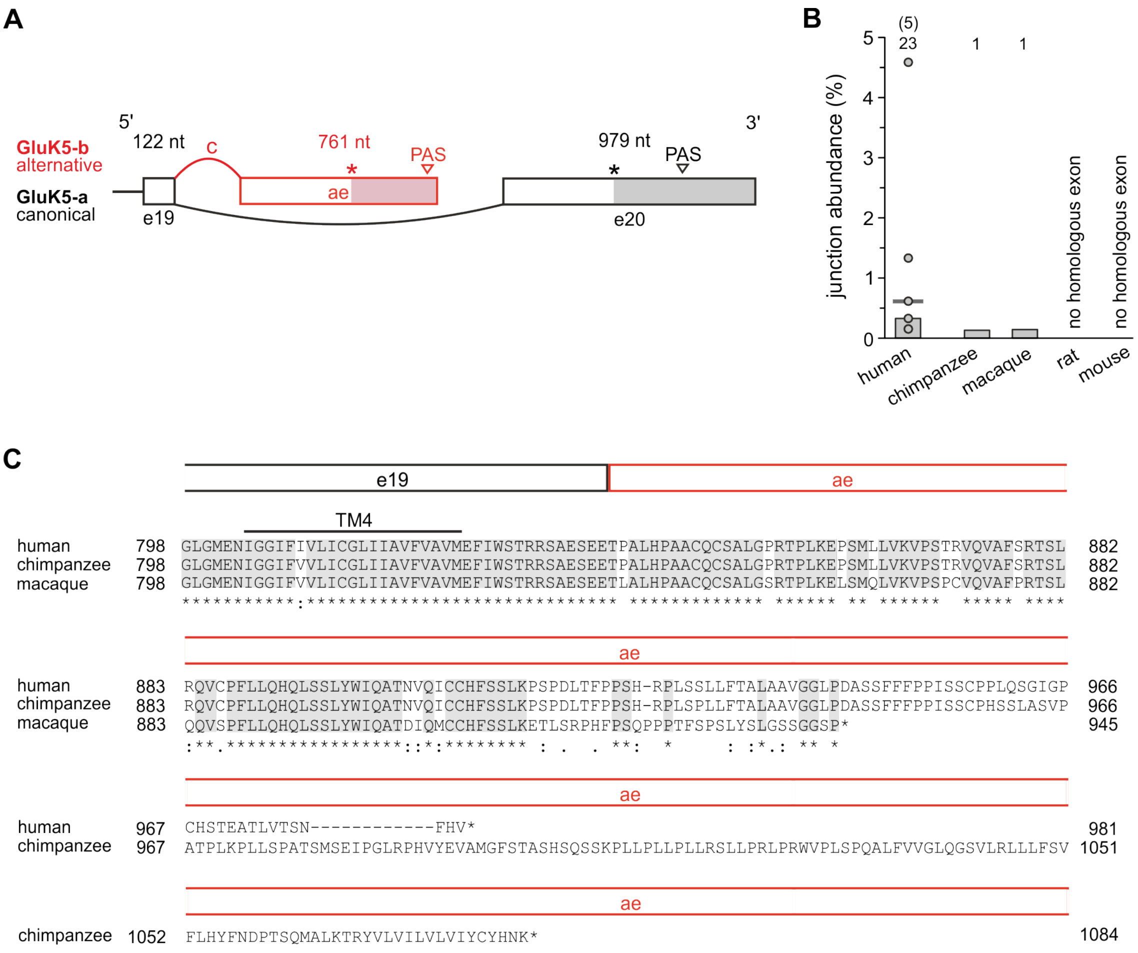

Figure S17. Potential GluK5-b isoform. (A) The Ensembl database lists a human transcript (ENST00000301218.8) with an alternative last exon (ae, 761 nt), which contains an alternative stop codon, a 3'-UTR and a polyadenylation signal (PAS). The resulting isoform, GluK5-b (981 aa) has also been mentioned in review articles (Traynelis *et al.*, 2010). **(B)** We detected the GluK5-b splice junction (*GRIK5* (c)) in humans, albeit with very low abundance, i.e. below our relevance criteria (see Main Text). Specific BLAST queries (**Table S6**) show that the junction is even less abundant in chimpanzee and macaque, whereas the corresponding exon seems to be absent in rat and mouse. Boxes show the global junction abundance with the total number of alternative reads given on top. Single points show the abundance in individual datasets with sufficient coverage (≥ 40 splice junction-spanning reads, human n = 5 datasets**,** median shown as bar). **(C)** Amino acid sequence alignment. The alternative C-terminal sequences from human, chimpanzee and macaque differ in the occurrence of their stop codon, which indicates a low level of conservation.

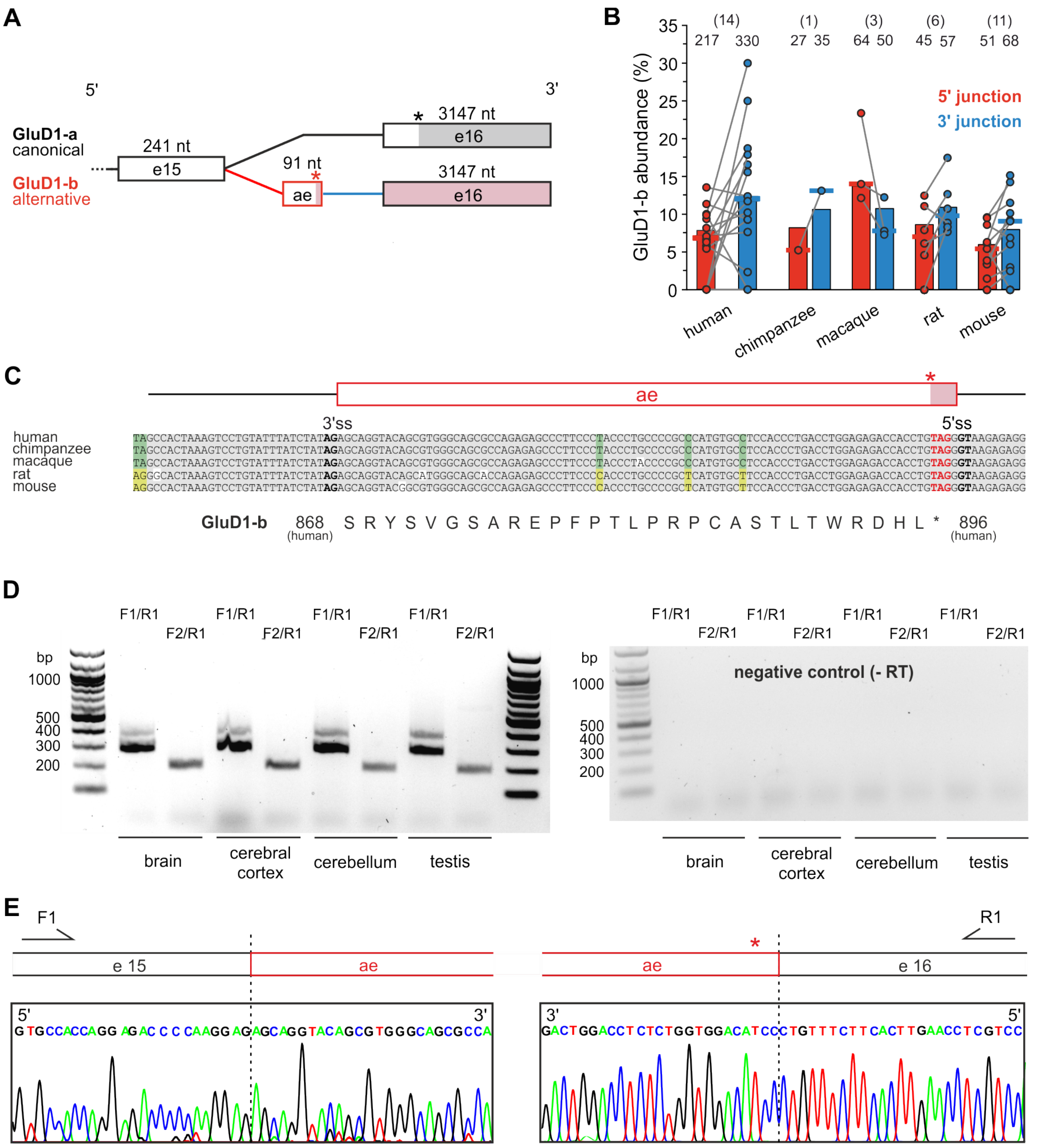

Figure S18. Additional characterization of the GluD1-b isoform. (A) Our analysis reveals the existence of two new junctions, *GRID1* (b-a), which indicate incorporation of a cassette exon (ae, 91 nt) between canonical exons e15 and e16 (see **Fig. 3**). **(B)** The splice junctions occur in human datasets and with similar abundance also in chimpanzee, macaque, rat and mouse. For human abundance see **Fig. 3B**; other abundances were obtained by junction-specific queries (**Table S6**). Boxes represent the global junction abundance with the total number of alternative reads given on top. Single dots represent the abundance in individual datasets with sufficient coverage (≥ 40 splice junction-spanning reads, number of datasets given in parentheses, median shown as bar). **(C)** Nucleotide alignment of the alternative exon region. The 3'-intron recognition site, the amino acid sequence, the stop codon and 5'-intron recognition sites are fully conserved across species. **(D)** RT-PCR detection of GluD1-b in human RNA samples from brain, cerebral cortex, cerebellum and testis (see **Fig. 3D/E**). Exon-specific primers F1/R1 show the relative

amounts of transcripts with and without alternative exon (expected product sizes: GluD1-a 285 bp; GluD1-b 376 bp). Primers F2/R1 are specific for the presence of the alternative exon (expected product size: 198 bp; see **Fig. 3D**). Bands are absent in negative controls without reverse transcriptase (-RT). **(E)** Sequence verification of the alternative exon boundaries after gel extraction of the PCR product using F1 and R1 as sequencing primers.

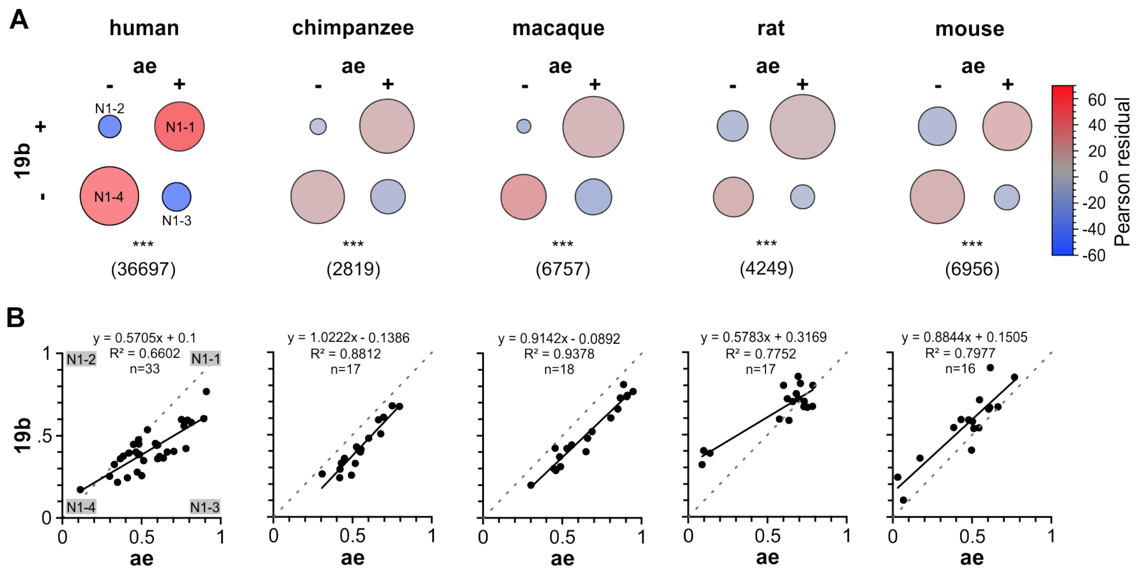

Figure S19. GluN1 splicing in the C-terminal region. Two alternative splice events produce four C-terminal isoforms of GluN1, GluN1-1 to GluN1-4 (see **Figure 4**). **(A)** Relative abundances of the four isoforms in different species. The circle sizes represent the relative read numbers of isoform-specific junctions, which were detected by alignment to the udrg (human, **Table S4**) or by using isoform-specific queries for chimpanzee, macaque, rat and mouse (**Table S6**). Statistical testing shows a strong interdependence of the two splice events, favoring GluN1-4 and GluN1-1 isoforms in all species (Pearson's chi-squared test of independence, *** $p \leq 0.0005$, number of total reads in parentheses). **(B)** Correlation between alternative acceptor site usage (19b) and cassette exon inclusion (ae) in individual datasets (solid lines: Pearson correlation analyses).

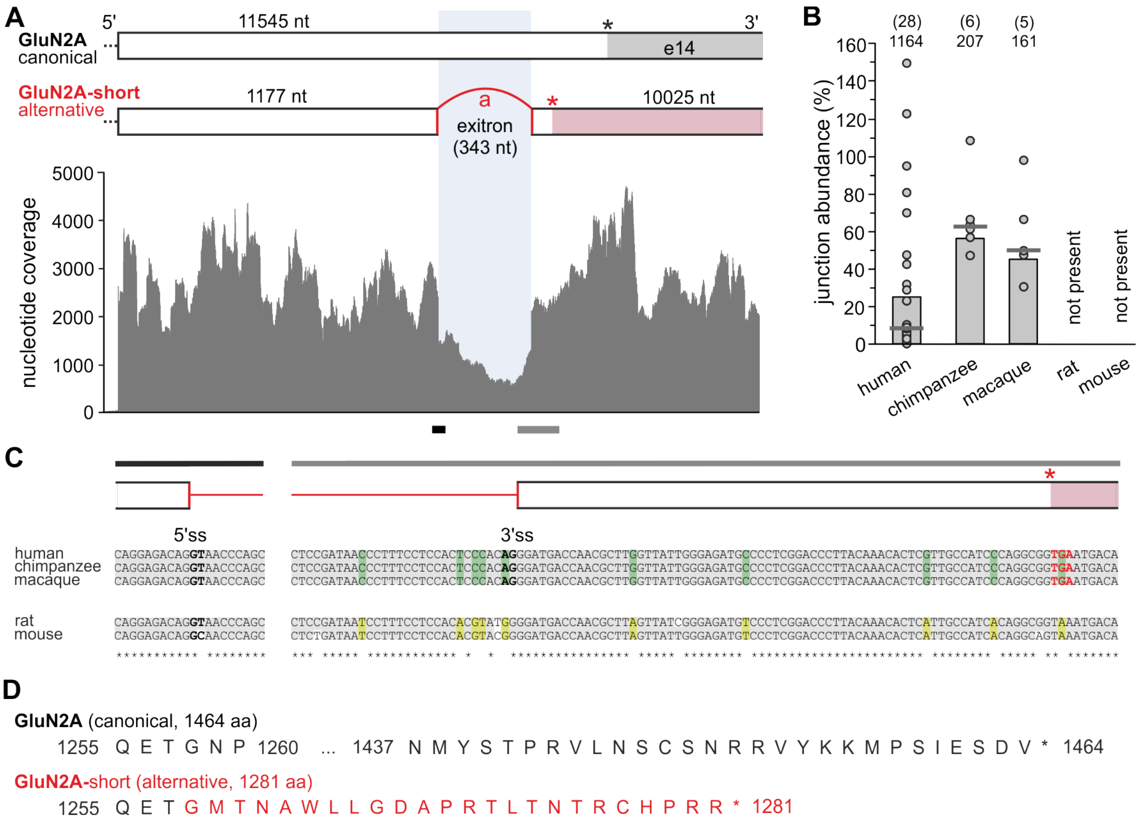

Figure S20. Primate-specific GluN2A-short isoform. (A) Our analysis identified an intra-exon junction, *GRIN2A* (a), which causes a 343 nt deletion (exitron) in the last canonical exon, e14. The same event has recently been characterized as primate-specific GluN2A-short isoform (Warming *et al.*, 2019). **(B)** The GluN2A-short junction is frequently detected in humans (**Fig. S9**), as well as in chimpanzee and macaque (**Table S6**). Single points represent the abundance in individual datasets with sufficient coverage (≥ 40 splice junction-spanning reads, number of datasets given in parentheses, median indicated as bar). **(C)** A nucleotide alignment shows that the exitron donor- and acceptor recognition sites (indicated in bold) are absent in rat and mouse. Further analysis shows that the recognition sites are present in all 44 primate species included in the Zoonomia Project alignment (with the exception of *Carlito syrichta*), whereas this combination of donor and acceptor recognition sites is absent in 196/197 of all non-primate species (Zoonomia Consortium, 2020); data not shown). **(D)** Amino acid sequence of the canonical and alternative GluN2A-short C-terminus in humans.

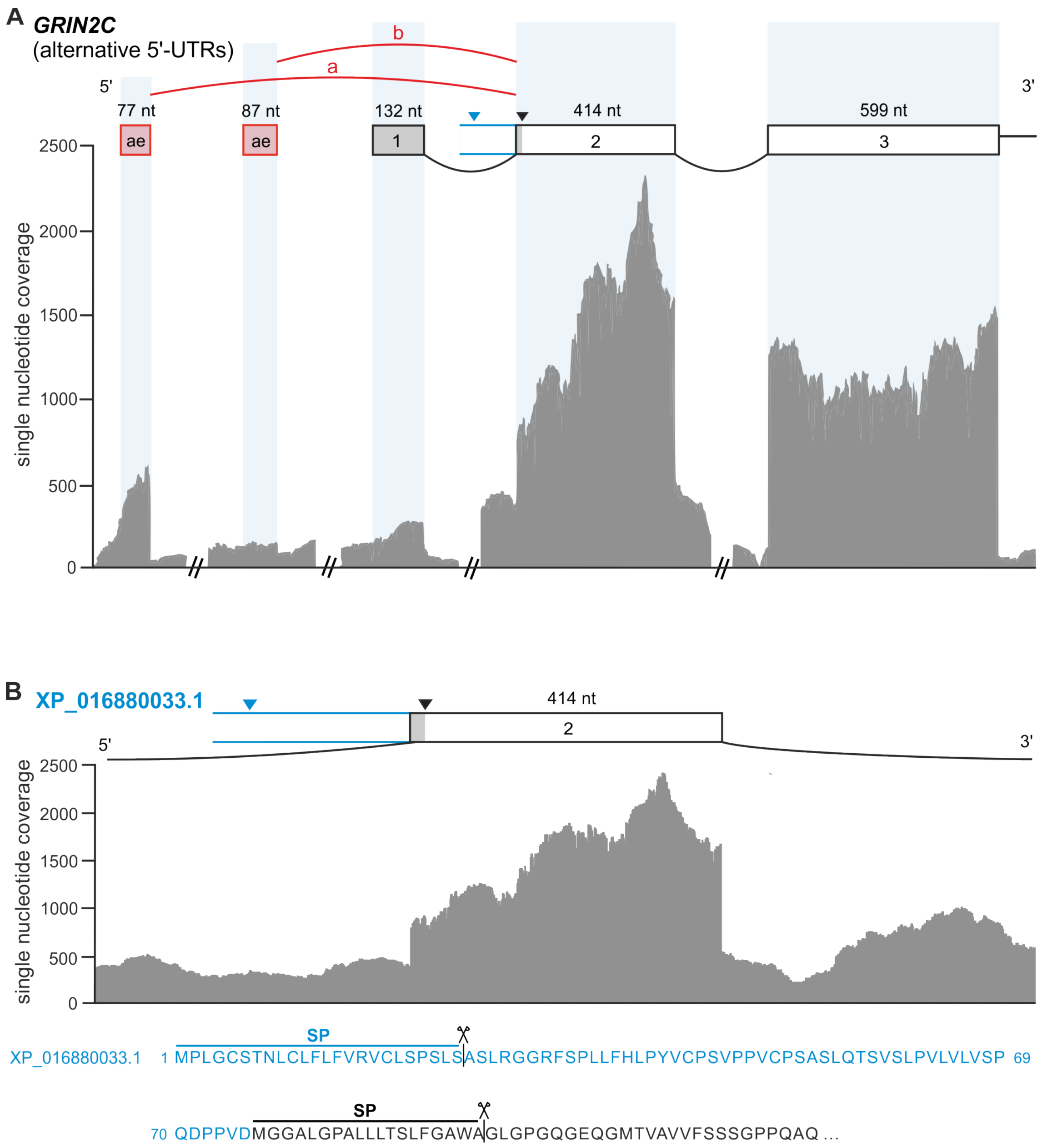

Figure S21. *GRIN2C* alternative 5'-UTRs and alternative start codon in humans. (A) Our *de novo* analysis identified two alternative junctions, *GRIN2C* (a) and *GRIN2C* (b), pointing to two alternative 5'-UTR exons (ae 77 nt and ae 87 nt; red). The junction abundance (**Table 4**) and coverage track indicate that ae 77 nt is used more frequently than the annotated canonical exon 1. **(B)** In addition, a human transcript with a 5'-elongated exon 2 has been reported (XP_016880033.1; blue), which contains an upstream start codon and signal peptide (SP) cleavage site. It may thus result in a N-terminal elongated isoform.

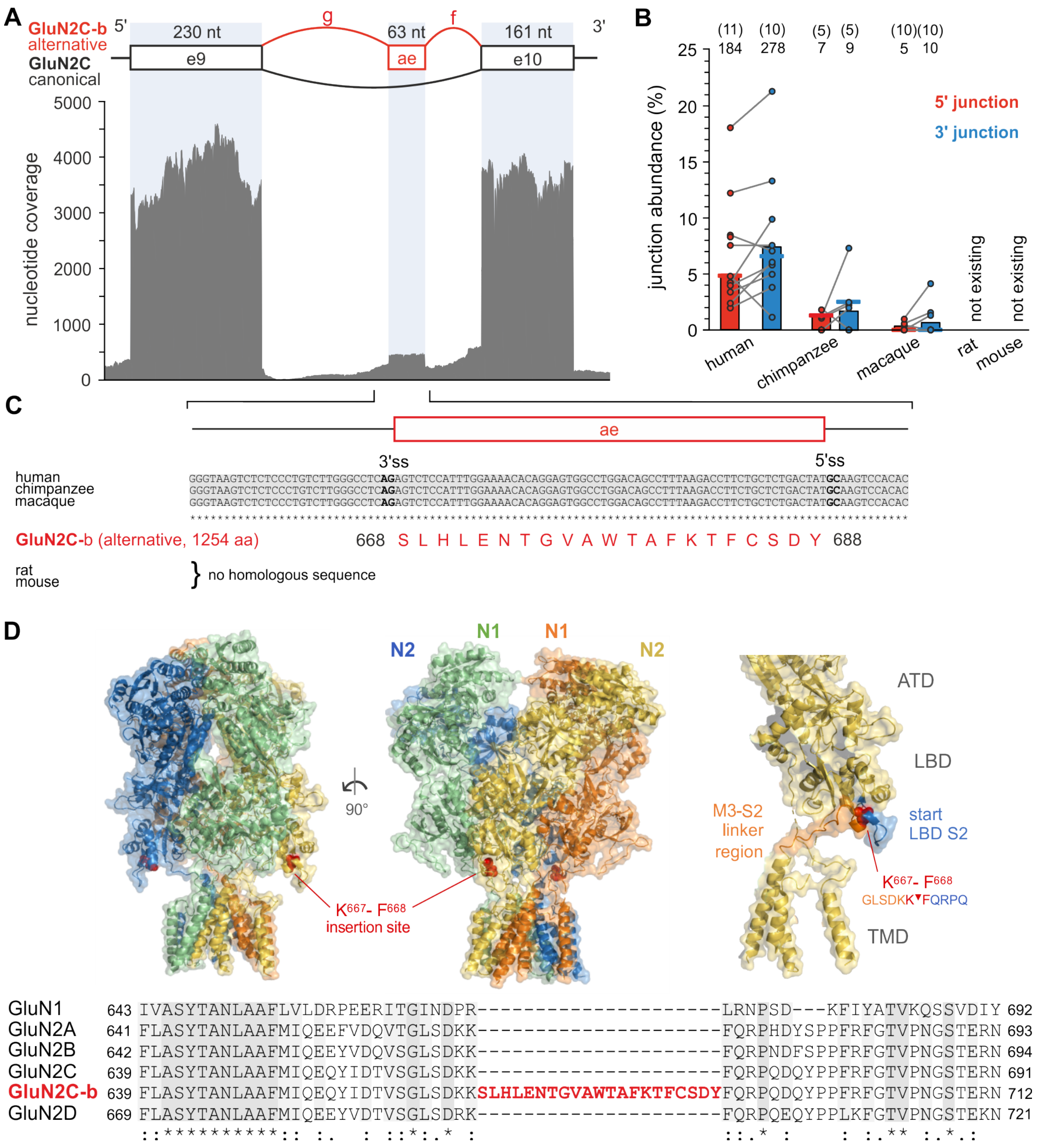

Figure S22. Potential primate-specific GluN2C-b isoform. (A) Our *de novo* analysis identified two alternative junctions, *GRIN2C* (g-f), which point to an alternative exon (ae; 63 nt) located between canonical exons e9 and e10. The coverage track shows a well-defined exon. **(B)** The corresponding junctions are observed with low to moderate abundance in humans, but more rarely in chimpanzee and macaque. They do not exist in rat or mouse. For human abundance see **Fig. S9**; other abundances were obtained by junction-specific queries (**Table S6**). Single dots represent the abundance in individual datasets with sufficient coverage (≥ 40 splice junction-spanning reads, number of datasets given in parentheses, median indicated as bar). **(C)** Nucleotide alignment. The alternative exon ae and the intron splice sites (3'- /5'-ss; indicated in bold) are fully conserved in human, chimpanzee and macaque, but they are not conserved across all primates (data not shown; Zoonomia Consortium, 2020). **(D)** The alternative exon may cause an 21 aa insertion after the M3-S2 linker region at the bottom of the LBD between K667 and F668. Figure based on GluN1/GluN2A, PDB 4PE5 (Karakas and Furukawa, 2014).

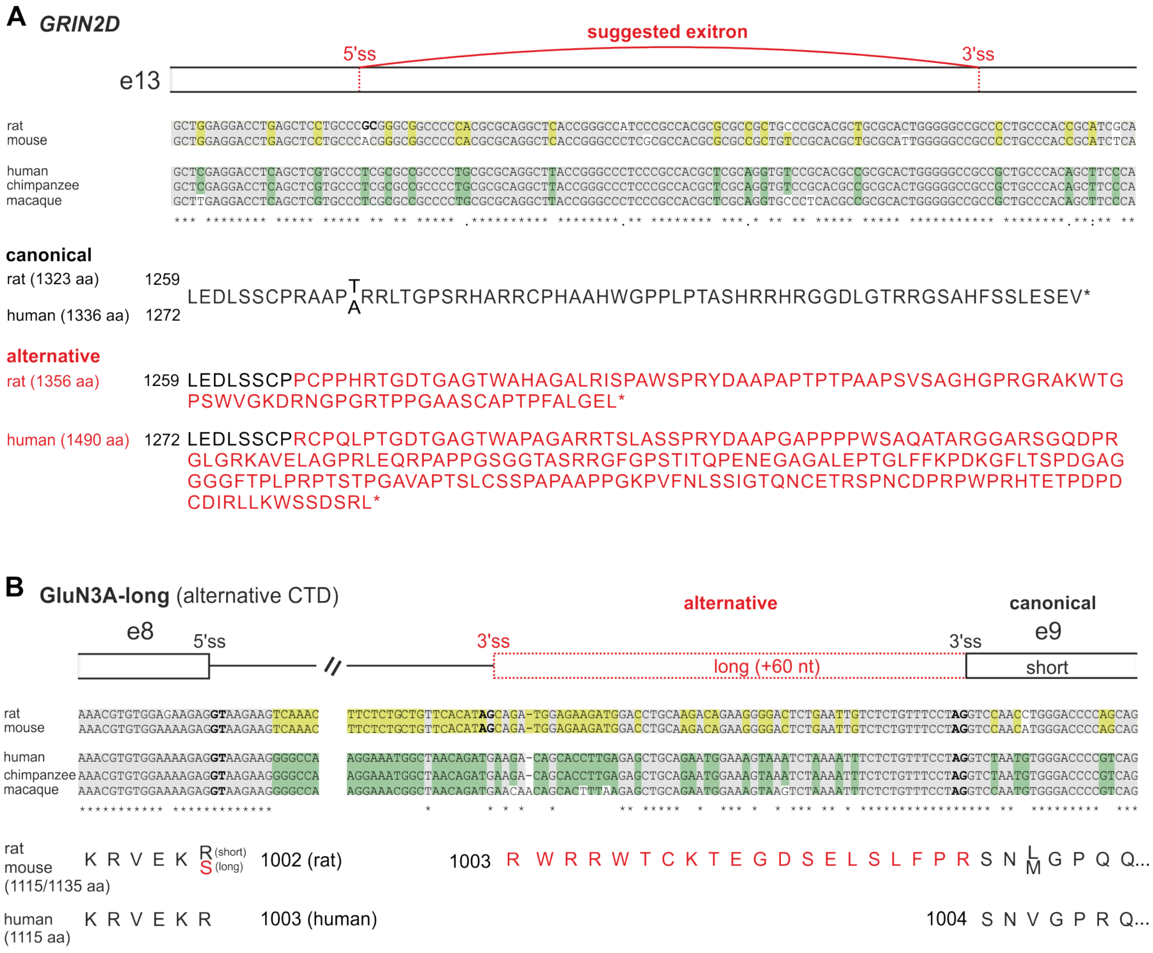

Figure S23. Non-conserved splice events in GluN2D and GluN3A. (A) For rat *GRIN2D* an intra-exon junction (exitron, -79 nt, ENSRNOT00000084869.1; (Ishii *et al.*, 1993) -82 nt) has been reported in the last canonical exon, but splice recognition sites are absent. **(B)** The GluN3A-long isoform does not occur in humans: In rat, an alternative splice acceptor site has been described, which causes a 60 nt 5'-extension of the last canonical exon and thus a 20 amino acid insertion in the CTD (Sun *et al.*, 1998). However, we did not detect the corresponding junction in human RNA-Seq datasets. The nucleotide alignment shows a 3'-intron recognition site (AG) in rat and mouse, but not in human, chimpanzee and macaque.

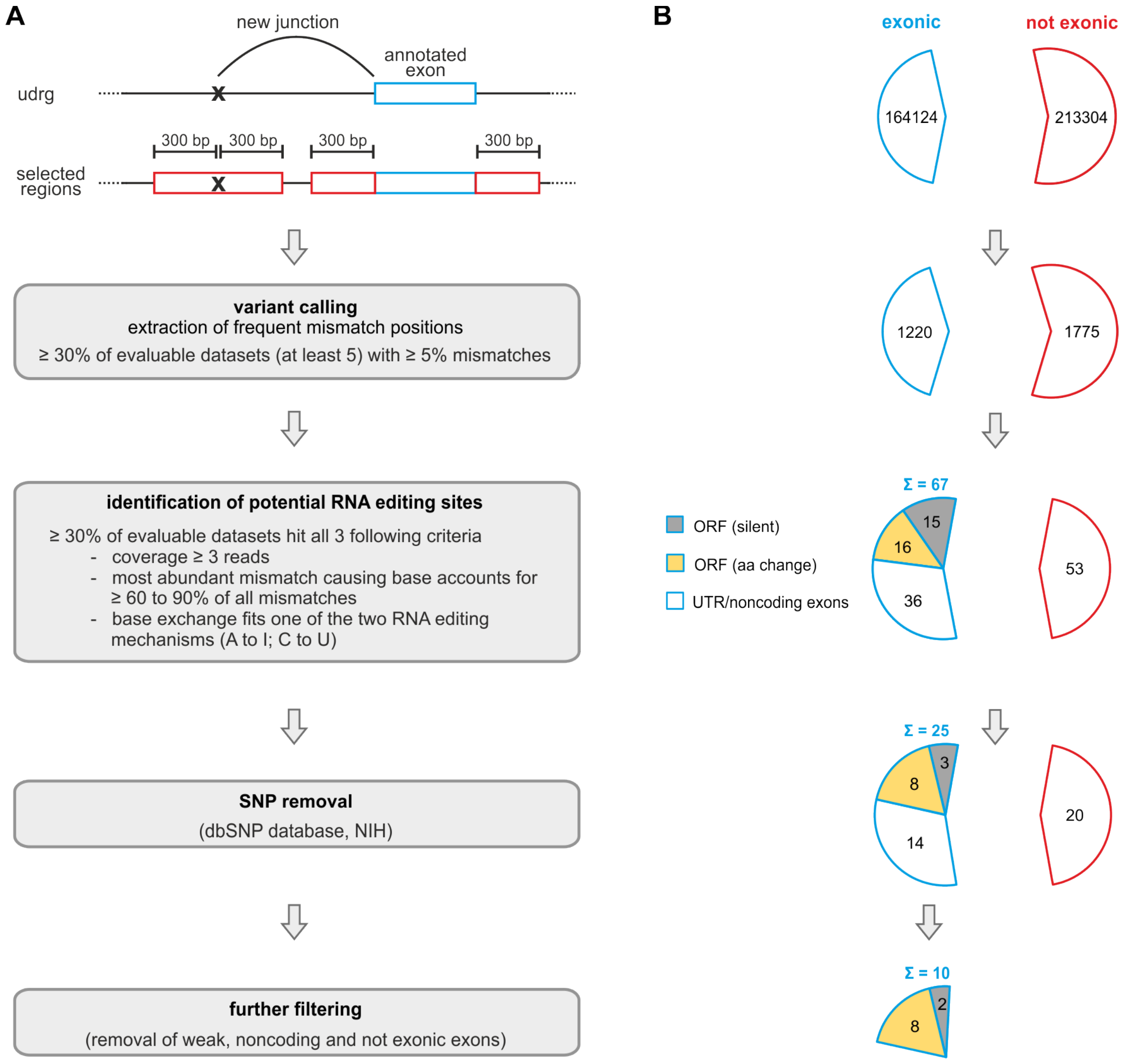

Figure S24. *De novo* identification of potential RNA editing sites. (A) *De novo* identification of potential RNA editing sites started with variant calling (SAMtools mpileup) of known exons and newly identified splice sites (new junction coverage ≥ 10 in at least two datasets or coverage ≥ 100) with ± 300 bp flanking regions. For details see SI Methods. Only frequently occurring mismatches were extracted and analyzed with criteria to identify potential RNA editing, followed by removal of known SNPs (**Fig. 5A** and **Fig. S25**). **(B)** Number of nucleotide positions after each analysis step, separated for exonic (blue) and non-exonic (red) positions.

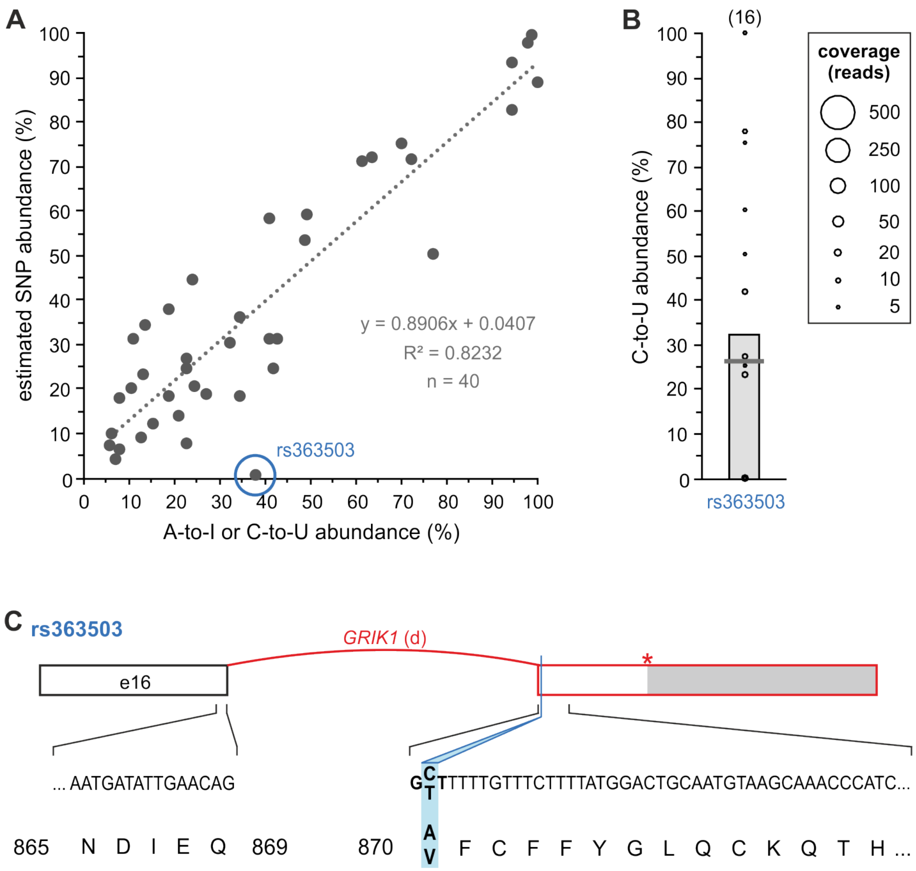

Figure S25. Comparison of frequent nucleotide mismatches to known SNPs. (A) A large fraction of potential editing sites in exonic regions (40 mismatches) matched SNPs reported in the dbSNP database (NIH). In most cases, the abundance of the observed A-to-I or C-to-U exchanges corresponded well to the SNP abundance estimated based on the reported minor allele frequencies (dotted line: Pearson correlation analysis). These mismatches were excluded from further analysis. Only in one case the mismatch frequency did not match the estimated SNP abundance, rs363503 (blue circle). **(B)** Abundance of rs363503 in individual datasets. This position was covered very poorly, as indicated by the low read numbers (datasets with ≥ 4 reads covering the position were included, *n* = 16 datasets). **(C)** rs363503 is located in an alternative last exon (after canonical exon 16) of *GRIK1*, which encodes for the rare GluK1-d isoform (see **Table 2** and **Fig. S13D**). It is located at the exon boundary (+2 nt) and results in the exchange A870V.

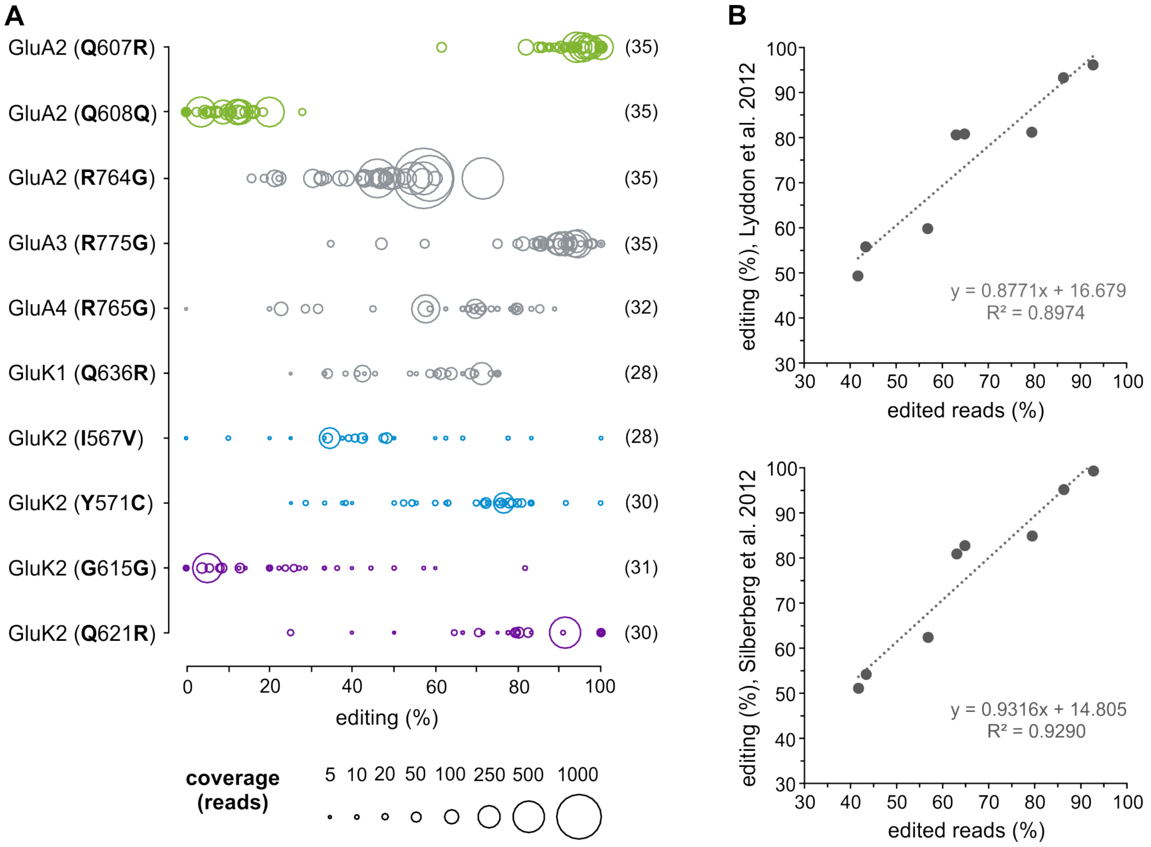

Figure S26. Frequency of the main iGluR editing events in coding regions. (A) Editing ratios within different datasets (cf. **Fig. 5B**). The circle size corresponds to the read coverage (only datasets with ≥ 4 reads covering the position were included; the number of datasets is given in parentheses). **(B)** Comparison of the mean editing ratios of the eight non-silent substitutions to previously reported editing ratios obtained with primer-based approaches (Lyddon *et al.*, 2012; Silberberg *et al.*, 2012). Pearson correlation analyses (dotted lines and indicated parameters) confirm good quantitative agreement. For minor editing events described in literature see **Table S7C**.

Supplementary Tables Overview

S1 Analyzed human RNA-Seq datasets p. 34

S2 Canonical iGluR transcripts p. 35

S3 Single nucleotide coverage of canonical iGluR transcripts .xlsx

A. Single nucleotide coverage of individual exons
B. Single nucleotide coverage in individual datasets and normalization

S4 Human iGluR splice junctions .xlsx

A. Identification of all iGluR junctions
B. Local abundance of relevant events

S5 Analysis of sex-specific splice junction differences .xlsx

A. Comparison of junction counts
B. Comparison of local abundances

S6 Analysis of splice junctions in other species .xlsx

A. RNA-Seq datasets
B. BLAST queries and counts

S7 Analysis of potential editing sites in human iGluR transcripts .xlsx

A. Identified mismatch positions
B. Potential editing sites
C. Other described editing events

S8 Co-occurrence of editing and splicing .xlsx

A. Close-by editing-editing events
B. Close-by editing-splicing events

S9 Deposited sequences (GenBank, NIH) p. 36

Supplementary Tables S1-2, S9

Table S1. Analyzed human RNA-Seq datasets

experiment	run	sample ID	tissue	age (years)	sex	ID[1]	size (Gbp)
			**Wu *et al.*, 2015**				
SRX512961[2]	SRR1222585	SAMN02721527	brain	adult	/	/	36.5[2]
SRX512962	SRR1222586	SAMN02721528	cerebellum	adult	/	/	27.8
SRX512963	SRR1222587	SAMN02721529	cerebral cortex	adult	/	/	27.3
SRX512964	SRR1222588	SAMN02721537	oblongata	adult	/	/	40.5
SRX512965	SRR1222589	SAMN02721533	frontal lobe	adult	/	/	47.0
SRX512966	SRR1222590	SAMN02721530	corpus callosum	adult	/	/	46.4
SRX512967	SRR1222592	SAMN02721535	insula	adult	/	/	45.7
SRX512969	SRR1222594	SAMN02721536	nucleus accumbens	adult	/	/	38.5
SRX512970	SRR1222599	SAMN02721538	parietal lobe	adult	/	/	44.7
SRX512971	SRR1222601	SAMN02721531	dura mater	adult	/	/	30.6
SRX513007	SRR1222677	SAMN02721539	pons	adult	/	/	46.9
SRX513008	SRR1222678	SAMN02721540	poster. central gyrus	adult	/	/	43.5
SRX513009	SRR1222680	SAMN02721541	precentral gyrus	adult	/	/	40.7
SRX513010	SRR1222682	SAMN02721542	putamen	adult	/	/	39.7
SRX513011	SRR1222684	SAMN02721543	substantia nigra	adult	/	/	26.6
SRX513012	SRR1222685	SAMN02721532	fetal brain	fetal	/	/	25.2
SRX513013	SRR1222686	SAMN02721534	hippocampus	adult	/	/	22.6
			**Labonté *et al.*, 2017**				
SRX3119920	SRR5961826	SAMN07499831	orbitofrontal cortex	52	m	1	4.4
SRX3119921	SRR5961827	SAMN07499830	orbitofrontal cortex	55	f	2	4.0
SRX3119922	SRR5961828	SAMN07499829	orbitofrontal cortex	79	f	3	5.2
SRX3119936	SRR5961842	SAMN07500103	orbitofrontal cortex	59	m	4	4.3
SRX3119937	SRR5961843	SAMN07500102	orbitofrontal cortex	51	f	5	5.0
SRX3119938	SRR5961844	SAMN07500105	prefrontal cortex[3]	47	m	6	3.3
SRX3119939	SRR5961845	SAMN07500101	prefrontal cortex[3]	41	m	7	5.2
SRX3119962	SRR5961868	SAMN07499938	prefrontal cortex[3]	25	f	8	2.7
SRX3119968	SRR5961874	SAMN07499932	prefrontal cortex[3]	52	m	1	11.4
SRX3120018	SRR5961924	SAMN07500047	cingulate gyrus 25	72	f	9	4.3
SRX3120019	SRR5961925	SAMN07500046	cingulate gyrus 25	41	f	10	4.0
SRX3120031	SRR5961937	SAMN07500034	cingulate gyrus 25	51	f	5	5.0
SRX3120032	SRR5961938	SAMN07500033	anterior insula	47	m	6	4.0
SRX3120065	SRR5961971	SAMN07499631	anterior insula	64	m	11	5.4
SRX3120066	SRR5961972	SAMN07499630	anterior insula	72	f	9	5.0
SRX3120112	SRR5962018	SAMN07499879	nucleus accumbens	79	f	3	7.3
SRX3120113	SRR5962019	SAMN07499878	nucleus accumbens	64	m	11	7.8
SRX3120155	SRR5962061	SAMN07499970	subiculum	52	m	1	5.4
SRX3120156	SRR5962062	SAMN07499968	subiculum	55	f	2	6.1

[1] donor ID; [2] excluded from analysis due to over-amplification; [3] dorsolateral prefrontal cortex;
All datasets are available via the Sequence Read Archive (SRA, NIH).

Table S2. Canonical iGluR transcripts

gene	Ensembl transcript ID	# exons	length (nt)	ORF (aa)	isoform synonym
GRIA1	ENST00000285900.9	16	5708	906	GluA1-flop
GRIA2	ENST00000264426.13	16	3445	883	GluA2-flop, short
GRIA3	ENST00000620443.1	16	4943	894	GluA3-flip
GRIA4	ENST00000282499.9	17	5508	902	GluA4-flip, long
GRIK1	ENST00000399913.5	17	3234	920	GluK1-1b
GRIK2	ENST00000421544.5	16	4789	908	GluK2-a
GRIK3	ENST00000373091.7	16	9101	919	GluK3
GRIK4	ENST00000527524.7	21	5861	956	GluK4
GRIK5	ENST00000593562.5	20	3308	980	GluK5-a
GRID1	ENST00000327946.11	16	5834	1009	GluD1
GRID2	ENST00000282020.8	16	6117	1007	GluD2
GRIN1	ENST00000371559.8	19	3577	885	GluN1-4a
GRIN2A	ENST00000396573.6	14	14450	1464	GluN2A
GRIN2B	ENST00000609686.3	13	30355	1484	GluN2B
GRIN2C	ENST00000293190.9	13	4261	1233	GluN2C
GRIN2D	ENST00000263269.3	13	5093	1336	GluN2D
GRIN3A	ENST00000361820.3	9	7770	1115	GluN3A
GRIN3B	ENST00000234389.3	9	3281	1043	GluN3B

Table S9. Deposited sequences (GenBank, NIH)		
**gene**	**event/junctions**	**accession**
GRIA4	isoform GluA4-ATD; (Table 1: junction a)	BK014574
GRIK1	predicted isoform GluK1(del_e2); (Table 2: junction e)	BK014575
GRIK2	transcript GluK2(alt_5'UTR); (Table 2: junction a)	BK014576
GRIK2	NMD transcript (cassette_171nt); (Table 2: junction e,f)	BK014577
GRIK4	transcript GluK4(alt_5'UTR); (Table 2: junction a,b)	BK014578
GRIK4	NMD transcript (altAcc_e11); (Table 2: junction d)	BK014579
GRIK4	isoform GluK4-2; (Table 2: junction e)	BK014580
GRIK5	NMD transcript (altDon_e2); (Table 2: junction a)	BK014581
GRIK5	NMD transcript (del_e13-e15); (Table 2: junction b)	BK014582
GRID1	isoform GluD1-b; (Table 3: junction a,b)	BK014583
GRIN1	NMD transcript (altAcc_e2); (Table 4: junction g)	BK014584
GRIN2B	transcript GluN2B (alt_5'UTR); (Table 4: junction a,b)	BK014585
GRIN2C	transcript GluN2C (alt_5'UTR_77nt); (Table 4: junction a)	BK014586
GRIN2C	transcript GluN2C(alt_5'UTR_87nt); (Table 4: junction b)	BK014587
GRIN2C	NMD transcript (altAcc_e11); (Table 4: junction d)	BK014588
GRIN2C	NMD transcript (altDon_e4); (Table 4: junction e)	BK014589
GRIN2C	isoform GluN2C-b; (Table 4: junction f,g)	BK014367
GRIN2C	predicted isoform GluN2C(del_2); (Table 4: junction h)	BK014590
GRIN2C	NMD transcript (del_e4); (Table 4: junction i)	BK014591

Supplementary Methods

The overall workflow is summarized in **Supplementary Figure S1 (Fig. S1)**. Further technical information on the *de novo* identification of splice junctions is provided in **Fig. S4**, information on the identification of potential editing sites in **Fig. S24**.

Alignment and initial analysis with NGS standard tools was performed on an unixoid system (Ubuntu 14.04.6 LTS) with an Intel Core i7-2600 processor, 8 GB RAM and Unix shell (version 4.3.11(1) as user interface. Subsequent analyses were performed with Matlab R2015a (Mathworks) scripts and Excel (Microsoft Office Pro+16) on a Windows-based PC.

Reference genomes

For our analysis we relied on the human genome assembly hg38 (GRCh38.p10; Genome Reference Consortium). Quality control of data sets was performed after alignment to the full hg38 using UCSC (UC Santa Cruz) GTF and BED annotation files. To reduce the amount of data in subsequent analyses, we constructed a user-defined reference genome (udrg) encompassing the 18 iGluR genes with 1 Mbp up- and downstream flanking regions. The corresponding genomic sequences were retrieved from the UCSC Genome Browser using the following URL queries: http://genome.ucsc.edu/cgi-bin/das/hg38/dna?segment=*chromosome:start position,end position* and stored in a multi-FASTA udrg.fa file. The corresponding annotations were downloaded using the UCSC Table Browser (Karolchik *et al.*, 2004) and stored in a *udrgannotation*.gtf file.

Human RNA-Seq datasets

RNA-Seq datasets from human brain tissues were prefetched from the Sequence Read Archive (SRA, NIH) with the SRA Toolkit 2.8.2 (NIH) using the './prefetch *RunID*' command and converted using './fastq-dump -I --split-files *RunID*.sra'.

For our analysis we relied on 35 paired-end datasets (**Supplementary Table S1**). 16 datasets (593.7 Gb) originated from the study of Wu *et al.* (Wu *et al.*, 2015), which was based on commercially-obtained poly(A)-selected RNAs (Clontech). In this study, cDNA libraries were generated by a standard Illumina protocol with subsequent Illumina HiSeq 2000 sequencing (100 nt read length). One other dataset from this study was excluded, since it showed clear signs of over-amplification (see '**Quality control'** and **Fig. S2**). Another 19 datasets (99.8 Gb) were taken from the study of Labonté *et al.* (Labonté *et al.*, 2017). In this study, total RNA extraction from 11 different individuals (Douglas-Bell Canada Brain Bank, Québec) was followed by DNaseI treatment and rRNA depletion using the RNeasy Micro Kit (Qiagen). The cDNA library was generated with a ScriptSeq Complete Gold Kit (Epicentre, Illumina) and sequenced on the Illumina HiSeq 2500 platform (50 nt read length).

Alignment

TopHat 2.1.0, which is a spliced read mapper (Kim *et al.*, 2013), was used to align the reads of individual datasets to the reference genomes. Alignments to hg38 were performed using the command 'tophat -p 4 -F 0.03 -G *annotation.gtf* hg38.fa *RunID*_1.fastq *RunID*_2.fastq'. By using option -G, reads were first aligned to the transcriptome, followed by an alignment of the

remaining reads to the complete reference genome. The minor isoform fraction parameter (-F) was reduced to 0.03, other parameters were default. Alignments to the udrg were performed using 'tophat -p 4 -splice-mismatches 1 -a 9 -F 0 -G *udrgannotation.gtf* udrg.fa *RunID_1*.fastq *RunID_2*.fastq'. We allowed single mismatches in the splice site anchor regions (option -splice-mismatches) to avoid a lack of junction detection because of single nucleotide variations, and thus increased the minimum anchor length from 8 to 9 (option -a) to compensate for the loss in specificity.

The alignment files (BAM) were sorted and indexed with SAMtools 0.1.19 (Li *et al.*, 2009) using the command 'samtools sort *alignment*.bam *sortedAlignment*.bam' followed by 'samtools index *sortedAlignment*.bam'.

Quality control

General alignment parameters, including the overall read mapping rate (**Fig. S2A**), were obtained from the *align_summary*.txt output of TopHat 2.1.0. Additional quality control parameters were collected using RSeQC v2.6.4 (Wang *et al.*, 2012). The intronic, exonic and intergenic mapping rates (**Fig. S2B,C**) were determined to obtain an estimate of genomic contaminations and pre-mRNA abundance using the command 'read_distribution.py –i *sortedAlignment*.bam –r *annotation.*gtf > *outputName*.txt'. We next tested the datasets for sufficient sequencing depth for the analysis of alternative splicing (**Fig. S2D**) using the command 'junction_saturation.py -i *sortedAlignment*.bam -r *annotation*.bed -o *outputName*.txt'. This tool extracts the number of newly identified and known splice junctions (based on the annotation file) after down-sampling the sorted BAM files to different extent (5%, 10%, …, 100%). In addition, we checked for over-amplification (**Fig. S2E**) by counting the number of read duplicates (reads with identical sequence) within canonical exons (for definition of canonical exons see '**Transcript information'**). For this we used sorted and indexed BAM files from the udrg alignment. Moreover, Phred quality scores of reads aligned to canonical exons were extracted from sorted and indexed BAM files and analyzed using a custom-written Matlab script (**Fig. S2F,G**).

Analysis of iGluR coverage

The single nucleotide coverage of canonical iGluR exons (**Fig. 1B**) and other selected iGluR regions (**Fig. 3A**; **Fig. S3**; **Fig. S11A**; **Fig. S12A**; **Fig. S14A**; **Fig. S15A**; **Fig. S20A**; **Fig. S21**; **Fig. S22A**) was determined by variant calling with SAMtools 0.1.19 (Li *et al.*, 2009) using the mpileup function (for details see 'variant calling'). The raw counts over all 35 datasets are reported.

In order to compare the abundance of iGluRs across different datasets (**Fig. S3**), we calculated the mean single nucleotide coverages of the canonical transcripts using the mean single nucleotide coverages of their individual exons (**Table S3B**). We then normalized these values to the number of mapped bases (alignment to hg38; TopHat Alignment Summary; (number of mapped reads)*(read length)/10^{10}) to account for dataset size (**Table S3B**).

Analysis and *de novo* identification of human iGluR splice junctions

Transcript information and canonical transcripts. Information on annotated iGluR transcripts, their exons, and the corresponding splice junctions was collected from the transcript annotation of the Ensembl 94 release (Aken *et al.*, 2016) and stored in a Matlab-based isoform database, which included the splice donor and acceptor positions of 379 known iGluR junctions (cf. **Table S4**). For referencing purposes, canonical transcripts were defined for all 18 iGluR genes (for details see **Table S2**), which typically encompassed the most frequent splice junctions in our datasets. The canonical transcripts contain 259 canonical junctions.

Identification of splice junctions. Detected splice junctions were classified with a custom-written Matlab pipeline (**Fig. S1**). First, the entries in the TopHat2 *udrgjunctions*.bed files were corrected by their block sizes to obtain the corresponding splice donor and acceptor site positions. Then the information on all junctions from all investigated datasets was collected in a junction count matrix. Junctions with known combinations of splice donor and acceptor sites were extracted as 'known' junctions (**Fig. S4A**). Subsequently, we searched for 'primary new junctions', i.e. junctions, which contain at least one known splice site. These junctions can be either new combinations ('C') of two splice sites, or they can contain one novel donor ('D') or acceptor site ('A'). Newly identified splice sites were then used as anchor points for subsequent junction identification rounds to obtain 'secondary new junctions' i.e. junctions with both, previously unknown donor and acceptor sites. This process was repeated 2 times until no further new splice sites were identified. Information on all known and newly identified iGluR junctions and their read counts are listed in **Table S4**.

Analysis and quantification of splice junctions. The extracted junction information was used for further analysis. Canonical splice junctions were normalized to the mean of the canonical junctions of the corresponding gene (**Fig. 2A**). For absolute numbers of canonical junction coverage see **Fig. S7**. The read distributions of all junctions are shown in **Fig. S5**.

Alternative junctions were normalized with regard to the closest canonical splice junction (the canonical junction, the mean of the canonical junctions with identical splice sites, or the closest canonical junction if there was no common splice site with the canonical transcript, see **Table S4**). The global junction abundance is based on the read counts across all 35 data sets (summed read counts). The local junction abundance based on the read counts in individual sets (**Table S4B**, **Fig. S9**), but was only calculated, if the alternative junction and the corresponding canonical junction in sum were covered ≥ 40 times.

All junctions and abundances are reported in **Table S4**. For further analysis we defined more abundant junctions as 'relevant junctions', if they were covered by at least 35 reads and if they had either a global abundance ≥ 5%, or, a local abundance ≥ 15% in at least one dataset (maximal local abundance). The local abundance criterion allowed for the detection of potentially tissue-enriched events. The relevant alternative splice junctions are listed in **Main Tables 1-4**.

In addition, **Main Tables 1-4** also contain some junctions, which were non-relevant according to our criteria. These were included, if they appeared to be directly related to a relevant junction (i.e. junctions defining an exon), or, if the corresponding isoform had been described in literature.

The amount of intron retention (prolonged e15 of the human *GRIK3*; **Fig. S15B**) was calculated by using the mean coverage of e15 and the mean coverage of the 369 nt prolongation. The global and local abundance was calculated as described for splice junctions.

Analysis of sex-specific differences in splicing. The datasets from Labonté *et al.* (Labonté *et al.*, 2017) were analyzed for sex-specific differences in splicing. Ten of the investigated datasets originated from female donors, nine from male donors (**Table S1**). First, the raw counts of junction-spanning reads of all iGluR junctions that were detected in at least four male and four female datasets ($n = 278$ junctions) were tested for sex-specific differences using a Mann-Whitney *U* test (Matlab ranksum function; **Table S5A**). No junction was detected at significantly different levels after Bonferroni correction for multiple comparisons (significance level $p < 0.05$), which suggests similar expression levels. Second, alternative splice events were analyzed based on local abundances. Events for which local abundances were available in at least four male and four female datasets ($n = 27$ events) no significant differences were found using a Mann-Whitney *U* test and Bonferroni correction (**Table S5B**).

Analysis of sequence motifs, alignments and structural inspections

For the analysis of splice site consensus sequences genomic sequences of the splice site and 150 bp flanking regions were extracted from the *udrg*.fa file taking the gene orientation into account. Sequence logos (**Fig. S6**) were generated for canonical, known alternative and new alternative splice sites using WebLogo 2.8.2 (Crooks *et al.*, 2004). Polyadenylation siganls (PAS) depicted in cartoons (**Fig. S12A, S15A, S17A**) were predicted using RegRNA2.0 (Chang *et al.*, 2013). Transcripts with a premature stop codon (>50 bp upstream of the last splice junction; (Kurosaki *et al.*, 2019) were classified as potential NMD transcripts. Nucleotide or amino acid alignments (**Fig. S10-18, S20, S22, S23**) were performed with ClustalOmega (Sievers *et al.*, 2011), and manually adjusted (**Fig. S11, S23B**). Pymol (DeLano, 2002) was used for structural inspections and cartoon representations (**Fig. S13, S16, S22**).

Quantification of selected splice junctions in other species

To evaluate evolutionary conservation of selected splice events, we quantified homologous events in chimpanzee, macaque, rat and mouse. For that purpose, we performed MegaBLAST (Morgulis *et al.*, 2008) searches with splice junction-specific queries in RNA-Seq datasets of these species (**Supplementary Table S6**; (Avrampou *et al.*, 2019; Cortez *et al.*, 2014; Darbellay and Necsulea, 2020; Mohn *et al.*, 2017; Soula *et al.*, 2018; Sousa *et al.*, 2017)). The query sequences were 40 bp long and centered on the splice junction; query specificity was checked beforehand with BLAST searches. Only MegaBLAST hits with a minimum query coverage of 95% and sequence identity of 95% were counted.

The abundance of alternative junction/s was calculated with regard to the canonical junction/s as described for human datasets. Global isoform abundances were calculated with the total read counts in all datasets. Isoform abundances in individual datasets were only calculated, if canonical and alternative junctions in sum were covered with ≥ 40 reads (**Fig. S12B, S13C, S14C, S15B, S16B, S17B, S18B, S20B, S22B**).

Analysis of alternative splicing events in the C-terminal region of GluN1. The combination of two alternative splice events creates four different C-terminal GluN1 sequences (**Fig. 4A**). We used our junction counts for the four isoform-specific splice junctions to analyze the co-occurrence of these events within individual human datasets (**Fig. 4B-D**). Only datasets with a cumulative junction count of all four splice junctions ≥ 40 were considered ($n = 33$). Pearson correlation analyses were performed using Excel. Mean probabilities of predictive splicing events are reported in **Fig. 4D**.

The distribution of the four C-terminal isoforms in other species (**Fig. S19**) was investigated by splice junction-specific MegaBLAST queries (compare 'quantification of selected splice junctions in other species') and analyzed for interdependence as described in 'analysis of editing-editing relationships'.

Analysis and *de novo* identification of potential RNA editing sites

Variant calling. All positions of annotated iGluR exons as well as newly identified splice sites (cumulative junction count ≥ 10 and detection in at least 2 datasets, or, junction count ≥ 100 in one dataset) were listed in a *positions*.bed file (**Fig. S1**), in both cases with 300 bp flanking regions (**Fig. S24A**). Variant calling was performed with SAMtools 0.1.19 (Li *et al.*, 2009) using the following command: 'samtools mpileup -Q 30 -d 20000 -aa -l *positions*.bed -f *udrg*.fa *sortedAlignment*.bam > *mpileupOutput*.txt'. The minimal Phred score (option -Q) was set to 30 to minimize sequencing error generated false positive results. The calling information was extracted from the *mpileupOutput*.txt files and collected in chromosome-separated matrices (.mat files) for fast retrieval and visualization using Matlab.

Extraction of frequent mismatch positions. To focus on potential RNA editing events, only mismatches, which occurred throughout a larger number of datasets were considered (**Fig. S24A** and **Table S7**): Only positions, where mismatches occurred in at least 5 different datasets and with at least 5% mismatch frequency in ≥ 30% of the datasets that covered the corresponding site, were extracted and analyzed further.

Identification of potential editing sites. Frequent mismatch positions were filtered further with criteria that prefer RNA editing (**Fig. S24A** and **Table S7**). Positions were considered, if in ≥ 30% of the datasets in which the position was covered by at least one read, i) the single nucleotide coverage of the position was ≥ 3, ii) the most common mismatch accounted for at least 60% (for coverage 3-5 reads), 75% (for coverage 6-17 reads) or 90% (for coverage ≥ 18 reads) of all mismatches, and iii) the base exchange was consistent with one of the two known RNA editing mechanisms (A-to-I or C-to-U editing). For these positions the frequency of reads representing the genomic base, the edited base and other mismatches were calculated (**Table S7A**).

Evaluation of potential editing events. Two potential editing sites being close to *GRIN3B* but belonging to gene *TMEM259* were discarded. The remaining 120 sites underwent manual evaluation (**Table S7B**). Due to the overall low intron coverage we further focused our subsequent analysis on exonic positions (**Fig. 5A**).

To exclude remaining SNPs, mismatches were next compared to known, genome-based SNPs (dbSNP; (Sherry *et al.*, 2001); NIH 1/24/2020). The A-to-I and C-to-U mismatch frequencies were estimated based on the reported minor allele frequency. In most cases, the obtained mismatch frequency was consistent with the reported SNP frequency and we thus discarded these events. Only a single event (rs363503) was detected more frequently than expected (**Fig. S25**), but also this potential C-to-U editing event was ultimately excluded from our analysis due to very low overall abundance. Mismatch positions in non-coding exons or weak resilience (different mismatches, low coverage, or differences between studies) are included in **Table S7B**, but are not reported in **Fig. 5B**.

Editing frequencies of common editing events (**Fig. 5B**) were calculated, if the coverage of the position was ≥ 4 within the respective dataset. The individual datasets frequencies were also used to calculate the mean frequencies (**Fig. S26B**). In addition, we determined the editing frequencies of other rare iGluR editing sites described in literature (**Table S7C**; (Herb *et al.*, 1996; Higuchi *et al.*, 2000; Silberberg *et al.*, 2012; Venø *et al.*, 2012; Wahlstedt *et al.*, 2009)).

Analysis of editing-editing and editing-splice relationships

Data collection. We evaluated the co-occurrence of close-by editing events (**Fig. 5C**) or close-by editing and splice events (**Fig. 5D**) in individual transcripts by performing MegaBLAST (Morgulis *et al.*, 2008) searches with event combination-specific queries (**Table S8**) in all 35 human datasets (**Table S1**) using the Sequence Read Archive Run Browser (NIH). The query length was 40 nt and gene specificity was checked beforehand using BLAST searches. To ensure detection of specific event combinations, only hits with 100% sequence identity and complete query coverage were counted.

Analysis of editing-editing relationships. The queries contained all four possible combinations of two adjacent editing events and were positioned within exons to avoid splicing-dependent effects. The absolute MegaBLAST hit counts (total sums) were used for further analysis (**Table S8A**). A Pearson's chi-squared test of independence (chisq.test function, R 3.5.0, The R Project) was applied to test for independence of the editing events, which was rejected at significance levels $p \leq 0.0005$ (***). For visualization (**Fig. 5C**), different circle sizes were chosen to reflect the relative combination abundance, whereas the color depicts the corresponding Pearson residuals $r = (o - i) / i^{0.5}$ with o denoting 'observed hits', and i denoting 'expected hits in case of independence'.

Analysis of editing-splicing relationships. For the analysis of editing-splicing relationships, editing sites in close proximity to splice junctions were investigated (distance to splice junction ≤ 10 nt; **Table S8B**). The queries (40 nt) were designed to have the splice junction close to the center of the query, and alternative splicing as well as intron retention was considered. Alternative cassette exons in *GRIA2* and *GRIA4* (**Fig. S10B**) were queried, but not included for further analysis due to low abundance (**Table S8B**). Pearson's chi-squared test of independence was used as described above.

RT-PCR validation of alternative splicing events

Several alternative splicing events were verified by reverse transcriptase-PCR. RNA samples from different human tissues were purchased from BioCat (BioChain, total RNA, DNase-treated; brain: male, 29 years (R1234035/C210018); cerebral cortex: male, 27 years (R1234042/B810027); cerebellum: male, 29 years (R1234039/B910013); testis: male, 24 years (R1234260/B703033). cDNA synthesis was performed using the Maxima H Minus First Strand cDNA Synthesis Kit (Thermo Scientific) with oligo(dT)$_{18}$ primers. PCRs were performed with the following exon-spanning primer pairs: *GRIA4* (**Fig. S12D**, F1: 5'-GTTGGTTACTGGAATGATATGG, R1: 5'-CACACAGAACAGGCAAAATTCC, R2: 5'-GTGTCTGCATCCCTTGCTC, annealing 58°C), *GRIK4* (**Fig. S16C,** F1: 5'-CCGAACTTGGGATGGTGTCAG, R1: 5'-CAATGTGGCCGGTAAGACCTTC, annealing 66°C) and *GRID1* (**Fig. 3D** and **Fig. S18D**, F1: 5'-GCTTCGCTGGGGTCTTCTG, F2: 5'-CCTGACCTGGAGAGACCACC, R1: 5'-GCTGGGTGTTCTGGTACTCC, annealing 58°C). Primers were obtained from Sigma-Aldrich and specificity was checked beforehand using Primer-Blast (NIH). PCRs were performed using Q5 High-Fidelity DNA polymerase and GC-Enhancer (New England Biolabs) on RoboCycler 96 (Stratagene) thermocyclers (denaturation 98°C, annealing as indicated, elongation 72°C, 30 - 40 cycles). Identically processed samples without addition of reverse transcriptase (-RT) served as negative control. PCR products were separated by agarose gel electrophoresis, stained with ethidium bromide, and semi-quantitative analysis of gel images was performed with ImageJ 1.51f (Schneider *et al.*, 2012) according to the recommendations of the NIH. A 100 bp DNA ladder (New England Biolabs) served as standard. Data are based on two to three independent cDNA syntheses and three to four independent PCR reactions. **Fig. 3D** and **Fig. S18D** show different examples, **Fig. 12D** and **Fig. 16C** show representative examples. For junction verification, bands or PCR products were extracted, purified (NucleoSpin Gel and PCR Clean-Up-Kit, Macherey-Nagel) and sequenced with forward and reverse primers.

Supplementary References

Aken, B.L., Ayling, S., Barrell, D., Clarke, L., Curwen, V., Fairley, S., Fernandez Banet, J., Billis, K., García Girón, C., Hourlier, T., Howe, K., Kähäri, A., Kokocinski, F., Martin, F.J., Murphy, D.N., Nag, R., Ruffier, M., Schuster, M., Tang, Y.A., Vogel, J.H., White, S., Zadissa, A., Flicek, P., Searle, S.M., 2016. The Ensembl gene annotation system. *Database 2016, baw093* 2016.

Avrampou, K., Pryce, K.D., Ramakrishnan, A., Sakloth, F., Gaspari, S., Serafini, R.A., Mitsi, V., Polizu, C., Swartz, C., Ligas, B., Richards, A., Shen, L., Carr, F.B., Zachariou, V., 2019. RGS4 maintains chronic pain symptoms in rodent models. *J Neurosci* 39, 8291-8304.

Bettler, B., Boulter, J., Hermans-Borgmeyer, I., O'Shea-Greenfield, A., Deneris, E.S., Moll, C., Borgmeyer, U., Hollmann, M., Heinemann, S., 1990. Cloning of a novel glutamate receptor subunit, GluR5: expression in the nervous system during development. *Neuron* 5, 583-595.

Chang, T.H., Huang, H.Y., Hsu, J.B., Weng, S.L., Horng, J.T., Huang, H.D., 2013. An enhanced computational platform for investigating the roles of regulatory RNA and for identifying functional RNA motifs. *BMC Bioinformatics* 14 Suppl 2, S4.

Coleman, S.K., Cai, C., Kalkkinen, N., Korpi, E.R., Keinänen, K., 2010. Analysis of the potential role of GluA4 carboxyl-terminus in PDZ interactions. *PloS One* 5, e8715.

Cortez, D., Marin, R., Toledo-Flores, D., Froidevaux, L., Liechti, A., Waters, P.D., Grützner, F., Kaessmann, H., 2014. Origins and functional evolution of Y chromosomes across mammals. *Nature* 508, 488-493.

Crooks, G.E., Hon, G., Chandonia, J.M., Brenner, S.E., 2004. WebLogo: a sequence logo generator. *Genome Res* 14, 1188-1190.

Darbellay, F., Necsulea, A., 2020. Comparative transcriptomics analyses across species, organs, and developmental stages reveal functionally constrained lncRNAs. *Mol Biol Evol* 37, 240-259.

DeLano, W.L., 2002. Pymol: An open-source molecular graphics tool. *CCP4 Newsletter Protein Crystallography* 40, 82-92.

Dev, K.K., Nishimune, A., Henley, J.M., Nakanishi, S., 1999. The protein kinase C alpha binding protein PICK1 interacts with short but not long form alternative splice variants of AMPA receptor subunits. *Neuropharmacology* 38, 635-644.

Herb, A., Higuchi, M., Sprengel, R., Seeburg, P.H., 1996. Q/R site editing in kainate receptor GluR5 and GluR6 pre-mRNAs requires distant intronic sequences. *Proc Natl Acad Sci USA* 93, 1875-1880.

Higuchi, M., Maas, S., Single, F.N., Hartner, J., Rozov, A., Burnashev, N., Feldmeyer, D., Sprengel, R., Seeburg, P.H., 2000. Point mutation in an AMPA receptor gene rescues lethality in mice deficient in the RNA-editing enzyme ADAR2. *Nature* 406, 78-81.

Ishii, T., Moriyoshi, K., Sugihara, H., Sakurada, K., Kadotani, H., Yokoi, M., Akazawa, C., Shigemoto, R., Mizuno, N., Masu, M., 1993. Molecular characterization of the family of the N-methyl-D-aspartate receptor subunits. *J Biol Chem* 268, 2836-2843.

Jin, R., Singh, S.K., Gu, S., Furukawa, H., Sobolevsky, A.I., Zhou, J., Jin, Y., Gouaux, E., 2009. Crystal structure and association behaviour of the GluR2 amino-terminal domain. *EMBO J* 28, 1812-1823.

Karakas, E., Furukawa, H., 2014. Crystal structure of a heterotetrameric NMDA receptor ion channel. *Science* 344, 992-997.

Karolchik, D., Hinrichs, A.S., Furey, T.S., Roskin, K.M., Sugnet, C.W., Haussler, D., Kent, W.J., 2004. The UCSC Table Browser data retrieval tool. *Nucleic Acids Res* 32, D493-496.

Kim, D., Pertea, G., Trapnell, C., Pimentel, H., Kelley, R., Salzberg, S.L., 2013. TopHat2: accurate alignment of transcriptomes in the presence of insertions, deletions and gene fusions. *Genome Biol* 14, R36.

Kumar, J., Schuck, P., Mayer, M.L., 2011. Structure and assembly mechanism for heteromeric kainate receptors. *Neuron* 71, 319-331.

Kurosaki, T., Popp, M.W., Maquat, L.E., 2019. Quality and quantity control of gene expression by nonsense-mediated mRNA decay. *Nat Rev Mol Cell Biol* 20, 406-420.

Labonté, B., Engmann, O., Purushothaman, I., Menard, C., Wang, J., Tan, C., Scarpa, J.R., Moy, G., Loh, Y.E., Cahill, M., Lorsch, Z.S., Hamilton, P.J., Calipari, E.S., Hodes, G.E., Issler, O., Kronman, H., Pfau, M., Obradovic, A.L.J., Dong, Y., Neve, R.L., Russo, S., Kazarskis, A., Tamminga, C., Mechawar, N., Turecki, G., Zhang, B., Shen, L., Nestler, E.J., 2017. Sex-specific transcriptional signatures in human depression. *Nat Med* 23, 1102-1111.

Leonard, A.S., Davare, M.A., Horne, M.C., Garner, C.C., Hell, J.W., 1998. SAP97 is associated with the alpha-amino-3-hydroxy-5-methylisoxazole-4-propionic acid receptor GluR1 subunit. *J Biol Chem* 273, 19518-19524.

Li, H., Handsaker, B., Wysoker, A., Fennell, T., Ruan, J., Homer, N., Marth, G., Abecasis, G., Durbin, R., Subgroup, G.P.D.P., 2009. The sequence alignment/map format and SAMtools. *Bioinformatics* 25, 2078-2079.

Lyddon, R., Navarrett, S., Dracheva, S., 2012. Ionotropic glutamate receptor mRNA editing in the prefrontal cortex: no alterations in schizophrenia or bipolar disorder. *Psych & Neurosci* 37, 267-272.

McKetney, J., Runde, R.M., Hebert, A.S., Salamat, S., Roy, S., Coon, J.J., 2019. Proteomic atlas of the human brain in Alzheimer's disease. *J Proteome Res* 18, 1380-1391.

Mohn, E.S., Erdman, J.W., Neuringer, M., Kuchan, M.J., Johnson, E.J., 2017. Brain xanthophyll content and exploratory gene expression analysis: subspecies differences in rhesus macaque. *Genes Nutr* 12, 9.

Morgulis, A., Coulouris, G., Raytselis, Y., Madden, T.L., Agarwala, R., Schäffer, A.A., 2008. Database indexing for production MegaBLAST searches. *Bioinformatics* 24, 1757-1764.

Schiffer, H.H., Swanson, G.T., Heinemann, S.F., 1997. Rat GluR7 and a carboxy-terminal splice variant, GluR7b, are functional kainate receptor subunits with a low sensitivity to glutamate. *Neuron* 19, 1141-1146.

Schneider, C.A., Rasband, W.S., Eliceiri, K.W., 2012. NIH Image to ImageJ: 25 years of image analysis. *Nat Methods* 9, 671-675.

Sherry, S.T., Ward, M.H., Kholodov, M., Baker, J., Phan, L., Smigielski, E.M., Sirotkin, K., 2001. dbSNP: the NCBI database of genetic variation. *Nucleic Acids Res* 29, 308-311.

Sievers, F., Wilm, A., Dineen, D., Gibson, T.J., Karplus, K., Li, W., Lopez, R., McWilliam, H., Remmert, M., Söding, J., Thompson, J.D., Higgins, D.G., 2011. Fast, scalable generation of high-quality protein multiple sequence alignments using Clustal Omega. *Mol Syst Biol* 7, 539.

Silberberg, G., Lundin, D., Navon, R., Öhman, M., 2012. Deregulation of the A-to-I RNA editing mechanism in psychiatric disorders. *Hum Mol Genet* 21, 311-321.

Sobolevsky, A.I., Rosconi, M.P., Gouaux, E., 2009. X-ray structure, symmetry and mechanism of an AMPA-subtype glutamate receptor. *Nature* 462, 745-756.

Soula, A., Valere, M., López-González, M.J., Ury-Thiery, V., Groppi, A., Landry, M., Nikolski, M., Favereaux, A., 2018. Small RNA-Seq reveals novel miRNAs shaping the transcriptomic identity of rat brain structures. *Life Sci Alliance* 1, e201800018.

Sousa, A.M.M., Zhu, Y., Raghanti, M.A., Kitchen, R.R., Onorati, M., Tebbenkamp, A.T.N., Stutz, B., Meyer, K.A., Li, M., Kawasawa, Y.I., Liu, F., Perez, R.G., Mele, M., Carvalho, T., Skarica, M., Gulden, F.O., Pletikos, M., Shibata, A., Stephenson, A.R., Edler, M.K., Ely, J.J., Elsworth, J.D., Horvath, T.L., Hof, P.R., Hyde, T.M., Kleinman, J.E., Weinberger, D.R., Reimers, M., Lifton, R.P., Mane, S.M., Noonan, J.P., State, M.W., Lein, E.S., Knowles, J.A., Marques-Bonet, T., Sherwood, C.C., Gerstein, M.B., Sestan, N., 2017. Molecular and cellular reorganization of neural circuits in the human lineage. *Science* 358, 1027-1032.

Sun, L., Margolis, F.L., Shipley, M.T., Lidow, M.S., 1998. Identification of a long variant of mRNA encoding the NR3 subunit of the NMDA receptor: its regional distribution and developmental expression in the rat brain. *FEBS Lett* 441, 392-396.

Tan, H.L., Queenan, B.N., Huganir, R.L., 2015. GRIP1 is required for homeostatic regulation of AMPAR trafficking. *Proc Natl Acad Sci U S A* 112, 10026-10031.

Traynelis, S.F., Wollmuth, L.P., McBain, C.J., Menniti, F.S., Vance, K.M., Ogden, K.K., Hansen, K.B., Yuan, H., Myers, S.J., Dingledine, R., 2010. Glutamate receptor ion channels: structure, regulation, and function. *Pharmacol Rev* 62, 405-496.

Venø, M.T., Bramsen, J.B., Bendixen, C., Panitz, F., Holm, I.E., Öhman, M., Kjems, J., 2012. Spatio-temporal regulation of ADAR editing during development in porcine neural tissues. *RNA Biol* 9, 1054-1065.

Wahlstedt, H., Daniel, C., Ensterö, M., Ohman, M., 2009. Large-scale mRNA sequencing determines global regulation of RNA editing during brain development. *Genome Res* 19, 978-986.

Wang, L., Wang, S., Li, W., 2012. RSeQC: quality control of RNA-seq experiments. *Bioinformatics* 28, 2184-2185.

Warming, H., Pegasiou, C.M., Pitera, A.P., Kariis, H., Houghton, S.D., Kurbatskaya, K., Ahmed, A., Grundy, P., Vajramani, G., Bulters, D., Altafaj, X., Deinhardt, K., Vargas-Caballero, M., 2019. A primate-specific short GluN2A-NMDA receptor isoform is expressed in the human brain. *Mol Brain* 12, 64.

Wu, D.D., Ye, L.Q., Li, Y., Sun, Y.B., Shao, Y., Chen, C., Zhu, Z., Zhong, L., Wang, L., Irwin, D.M., Zhang, Y.E., Zhang, Y.P., 2015. Integrative analyses of RNA editing, alternative splicing, and expression of young genes in human brain transcriptome by deep RNA sequencing. *J Mol Cell Biol* 7, 314-325.

Zhawar, V.K., Kaur, G., deRiel, J.K., Kaur, G.P., Kandpal, R.P., Athwal, R.S., 2010. Novel spliced variants of ionotropic glutamate receptor GluR6 in normal human fibroblast and brain cells are transcribed by tissue specific promoters. *Gene* 459, 1-10.

Zoonomia Consortium, 2020. A comparative genomics multitool for scientific discovery and conservation. *Nature* 587, 240-245.
